# Stay-at-home and face mask policy intentions inconsistent with incidence and fatality during the US COVID-19 pandemic

**DOI:** 10.3389/fpubh.2022.990400

**Published:** 2022-10-13

**Authors:** Samuel X. Wu, Xin Wu

**Affiliations:** ^1^Department of Engineering, Rice University, Houston, TX, United States; ^2^Department of Neuroscience and Experimental Therapeutics, Texas A&M Health Science Center School of Medicine, Bryan, TX, United States

**Keywords:** COVID-19, stay-at-home, face masks, incidence, fatality

## Abstract

During the COVID-19 pandemic, many states imposed stay-at-home (SAH) and mandatory face mask (MFM) orders to supplement the United States CDC recommendations. The purpose of this study was to characterize the relationship between SAH and MFM approaches with the incidence and fatality of COVID-19 during the pandemic period until 23 August 2020 (about 171 days), the period with no vaccines or specific drugs that had passed the phase III clinical trials yet. States with SAH orders showed a potential 50–60% decrease in infection and fatality during the SAH period (about 45 days). After normalization to population density, there was a 44% significant increase in the fatality rate in no-SAH + no-MFM states when compared to SAH + MFM. However, many results in this study were inconsistent with the intent of public health strategies of SAH and MFM. There were similar incidence rates (1.41, 1.81, and 1.36%) and significant differences in fatality rates (3.40, 2.12, and 1.25%; *p* < 0.05) and mortality rates (51.43, 34.50, and 17.42 per 100,000 residents; *p* < 0.05) among SAH + MFM, SAH + no-MFM, and no-SAH + no-MFM states, respectively. There were no significant differences in total positive cases, average daily new cases, and average daily fatality when normalized with population density among the three groups. This study suggested potential decreases in infection and fatality with short-term SAH order. However, SAH and MFM orders from some states' policies probably had limited effects in lowering transmission and fatality among the general population. At the policy-making level, if contagious patients would not likely be placed in strict isolation and massive contact tracing would not be effective to implement, we presume that following the CDC's recommendations with close monitoring of healthcare capacity could be appropriate in helping mitigate the COVID-19 disaster while limiting collateral socioeconomic damages.

## Introduction

Coronavirus disease 2019 (COVID-19), now recognized as a multi-system disease, is caused by severe acute respiratory syndrome coronavirus-2 (SARS-CoV-2). In the last 2 years through August 2022, 600 million people have been infected with COVID-19, and over 6 million people have died associated directly or indirectly with COVID-19 worldwide. Before late 2020, due to the lack of prophylactic and effective therapeutic approaches, non-pharmaceutical interventions (NPIs) were the only option for potentially mitigating pandemic transmission and fatality (1). COVID-19 is believed to spread primarily through close contact from person-to-person mainly, utilizing the respiratory route (2). Community mitigation strategies following the United States Centers for Disease Control and Prevention (US, CDC) recommendations can lower the risk for disease transmission by limiting or preventing person-to-person interactions. The CDC recommended public health NPIs including patients' isolation, close contact quarantine, hand hygiene, social distancing, wearing face coverings when anywhere in public (especially when social distancing is difficult), and monitoring personal health. However, increased mask use in public during COVID-19, along with a global supply shortage, has led to the widespread use of homemade masks and face covering alternatives in 2020 (3–5). In conjunction with gowns, gloves, eye protection, and hand washing, the mask is a core component of the personal protective equipment (PPE) that clinicians use when caring for symptomatic patients and that researchers require in the infectious diseases fields (6, 7). Although, historically, wearing a face mask has been controversial among the general public during previous influenza-like illness seasons, this practice is believed to reduce the spread of COVID-19 because asymptomatic patients with COVID-19 can also spread the virus unknowingly (8–11). Because the COVID-19 pandemic is asynchronous and varies in transmission across the US, states differ in whether they require their citizens to follow lockdown or stay-at-home (SAH) orders and/or mandated face masks (MFMs) to limit the spread. Apart from the CDC recommendations, individual states began implementing various community mitigation policies including SAH and MFM orders as of March 2020.

The consequences of combating the pandemic will involve reducing both incidence rates and severity of the disease. However, direct investigations on how COVID-19 mitigation efforts at the policy-making level affected transmission (e.g., incidence rate) and severity [e.g., case-fatality ratios (CFRs)] in the entire population, under the same CDC and Food and Drug Administration (FDA) guidelines, as well as national standardized healthcare professional practice and hospital systems, are lacking. Any best modeling and randomized, double-blind sampling clinical trials finally have to be applied to the specific or entire population to verify their validity. The purpose of this study was to characterize the relationship between state-level mitigation strategies and COVID-19 consequences around the US that involved all available US pandemic data and the entire population, but by neither modeling (theoretical) nor selecting regional database (i.e., not sampling).

## Literature review

We conducted a literature search for scientific articles published using the available COVID-19 pandemic database in the US to examine the relationship between NPIs (e.g., SAH and face covering), and issues of disease transmission and severity. The overall consensus on the importance of public health NPIs for pandemic control, even only in biological vulnerabilities, is still uncertain and controversial, and the study on the effectiveness of NPIs is still limited (8, 12–14). Many articles characterizing public health strategies either focus on masking models, indirect SAH, and masking data or school closures (15–19). The results suggested that the SAH and MFM orders could help reduce activities associated with the community spread of COVID-19 by limiting or preventing non-familial person-to-person interactions outside the household (15, 20–22). These articles were reported using selected counties', cities', and schools' pandemic data with potential selection bias, lack of adjustment for confounders (e.g., vaccines and specific drugs), or lack of randomized trials (23, 24). Only limited articles directly investigated COVID-19 mitigation efforts regarding infection and/or CFR on the entire US pandemic database (25, 26). These studies indicated potential reductions in the incidence and mortality of COVID-19; however, these readings suggested many limitations, and all results should be interpreted with caution. Recent reports indicated that lockdowns during the COVID-19 pandemic have had little to no public health effects, including effects on the mortality rate (27, 28). However, it is very difficult to draw a comprehensive conclusion because various countries have differing lockdown policies, other NPIs, and disparities regarding the quality and quantity of their health professionals and hospital systems.

## Methods

### Study data

Data on COVID-19 cases were collected from CDC provisional counts of United States COVID-19 cases and deaths by states over time (Supplementary eTables 1–4). On 23 August 2020, there were more than five million positive cases and close to 170,000 fatalities among 330 million vulnerable populations from the 50 states and the District of Columbia (DC) in the USA. Other data sources, such as state health departments and the Johns Hopkins Coronavirus Resource Center, were examined as references and listed in Supplementary eTable 1. The hospital capacity and health professional data were collected from the American Hospital Association and the Association of American Medical Colleges (15, 29). Data on state and territorial mandatory SAH and MFM orders for the general public were obtained from government websites containing executive or administrative orders or press releases (Supplementary eTable 1) (15). Between 21 January 2020 (first positive case in the US) and 23 August 2020, governors of 35 states and the mayor of Washington DC signed orders mandating all individuals who can medically tolerate the wearing of a face mask to do so in public settings (e.g., public transportation, parks, and grocery stores). This practice applies both indoors and outdoors where maintaining six feet of social distancing might not always be possible. For states with no state-wide mandates (e.g., only for store employees, or only a couple of counties or cities in the states), we will recognize these states as no-MFM (n=16 states). New Hampshire was counted as a non-MFM state because face coverings are only required for all persons who attend scheduled gatherings for social, spiritual, and recreational activities of 100 people or more. Between 21 January 2020 and 23 August 2020, 43 states and Washington DC signed orders mandating citizens to stay-at-home or shelter-in-place (see Supplementary eTables 1, 2 for references on MFM and SAH state list) (15, 16).

### Experimental approach and experimental outcomes

**Figure 1:**
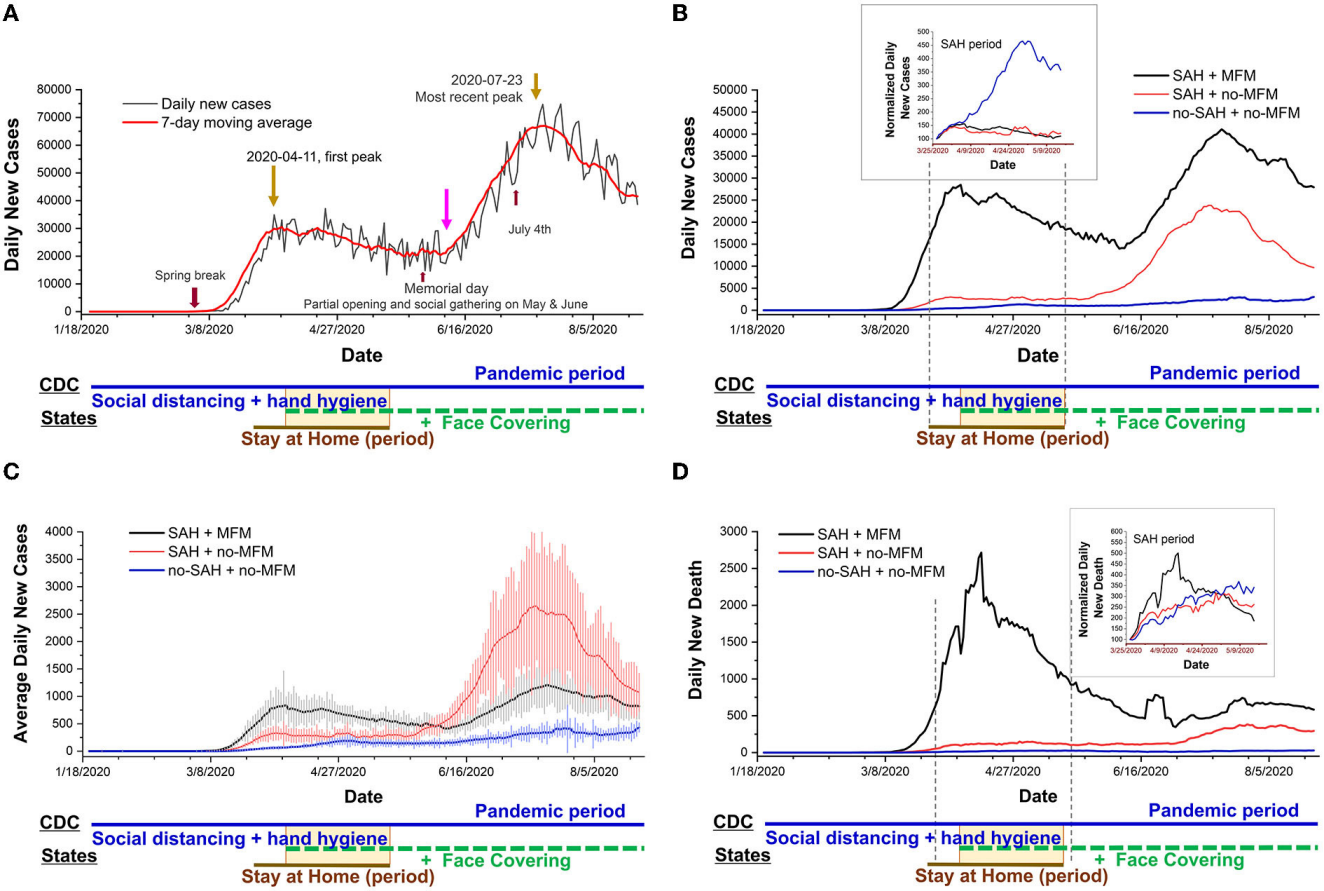
Daily trends in number of COVID-19 new cases in the US reported to CDC. Date is the time of the COVID-19 pandemic period (averaged at 171 days): from case 1 in the USA to 23 August 2020. Daily cases collected from 50 states and Washington DC. **(A)** Daily new cases raw data and 7-day averaged data. All holidays or events are indicated in the figure. The overlapping between states mandating stay-at-home and mandating face masks is emphasized with a yellow rectangular box. **(B)** The daily new case curves of the 7-day averaged are shown for mandating the stay-at-home (SAH) + mandating face mask (MFM) states (*n* = 34), the SAH + no-MFM states (*n* = 9), and the no-SAH + no-MFM states (*n* = 7) during the pandemic period. The curves are bending downward in the group of the MFM + SAH states and are flattened in the group of the MFM + no-MFM states during SAH periods (averaged at 45 days). The upper inset indicated normalized daily case data to the case of day 1 of SAH, which showed a significant increasing trend of new daily cases in the no-SAH + no-MFM states during the SAH period. **(C)** The average daily new case curves are seen for the three groups of the states. Each data represents the mean ± SE. **(D)** The daily new death curves of the 7-day averaged are seen for the three groups of the states. The upper inset indicated normalized daily new death data to the death number on day 1 of SAH which shows curve-bending downward in the SAH + MFM states during the SAH period.

The research design is a US population-based cross-sectional study. This study was designed to evaluate the efficacy of SAH and MFM at the policy-making level during the pandemic. The overall experimental protocol is shown in Figure 1. Separated four-state groups were based on their own policies as SAH + MFM states (*n* = 34), SAH + no-MFM states (*n* = 9), no-SAH + no-MFM states (*n* = 7), and no-SAH + MFM states (*n* = 1). The two primary outcomes examined are incidence and fatality. To examine the incidence, the incidence rate, positivity rate, and average daily cases were investigated with or without normalization to population density. To examine fatality, the mortality rate (per 100,000 residents), CFR, and average daily death were investigated with or without normalization to population density. Because COVID-19 is recognized as an acute disease, incidence rate or infection rate refers to the occurrence of new cases of COVID-19 in a state's vulnerable population over a specified pandemic period. *The pandemic period* is from the date of the first positive case in each state to the date of 23 August 2020, because there were no FDA-approved specific drugs or vaccines available yet. To examine the severity of disease, CFR was investigated as the proportion of people who have died associated directly or indirectly with COVID-19 among all infected over the *fatality periods* during the pandemic periods from the date of the first death in each state to 23 August 2020. The state-level COVID-19 testing rates and SARS-CoV-2 polymerase chain reaction (PCR) positivity rates were also examined.

### Statistical analysis

We employed an event study, which is similar to a difference-in-differences design, to examine whether state-wide mandated SAH and/or the use of face masks in public affect the spread of COVID-19 based on the state variations noted earlier. This design allowed us to estimate NPIs' effects in the context of a natural experiment, comparing the changes in COVID-19 spread among the states with mandates to changes in the states that did not pass these mandates, over a period of time. Each state's population density is defined as the number of people per square mile of state land area (Table 1).

**Table 1:** Characteristics of COVID-19 infection and population information.

**Groups^*$*^**	**SAH + MFM**	**SAH + no-MFM**	**No-SAH + no-MFM**
# of States and DC (populations)	34 (249,376,318)	9 (61,367,514)	7 (14,477,887)
Averaged incidence rate (%)	1.58	2.32	1.48
Averaged fatality rate (%)	3.50	1.86	1.31*
States with second peak	13	0 *	2
			
Days to first peak (or most recent peak if only 1 peak of that state)	97.82 ± 9.20	122.78 ± 9.66	121.85 ± 17.64
			
How many states with peak before the first peak on the date of 20200411 in Fig. 1	6	0	0
			
Days to most recent peak in Fig. 1	133.06 ± 7.93	122.78 ± 9.66	150.15 ± 5.67 ^&^
			
Averaged population density (residents/mile^2^)	561.09 ± 308.13 (257.24 ± 52.78 if excluded DC)	129.93 ± 36.24 *	28.43 ± 7.96*^, &^

MFM, mandating face mask; SAH, stay-at-home; #: numbers; DC, District of Columbia; $: no-SAH + MFM state: Arkansas (populations: 3,017,804). *p < 0.05 vs. SAH + MFM; ^&^p < 0.05 vs SAH + no-MFM. Each number is represented as the mean ± SE. Population density data see Supplementary eTables 1, 2.

Statistical tests were performed using SAS software (SAS Institute Inc., Cary, NC) and Origin 2020b (OriginLab Corporation, Northampton, MA). Categorical variables, such as two-peak occurrence (with peak fitting), were compared between groups using the Chi-square test (or Fisher exact test). Statistical comparisons of incidence rate and CFR were performed with a one-way or two-way analysis of variance (ANOVA) as appropriate. *Post-hoc* analyses were carried out to identify specific differences using Tukey's HSD (honestly significant difference) for multiple comparisons. In all statistical tests, differences were considered statistically significant at two-sided *p*-values < 0.05. Data were expressed as the mean ± standard error (SE).

## Results

### Trends in daily new COVID-19 cases in the US decreased due to stay-at-home orders and increased following social gathering events

The incubation period was defined as the interval between the potential earliest date of contact with the transmission source, and the potential earliest date of symptom onset or the earliest time a test could give a positive result. The incubation period for COVID-19 was reported to be anywhere from 5 days to 23 days for the original SARS-CoV-2 strain (30, 31). There were some cause-and-effect phenomena in the curve of Figure 1. In the 7-day averaged curve, there was a detectable increase following spring break (late February to early April) and a peak on 11 April 2020 (Figure 1A). In Figure 1A, the daily new cases curve of the nation was flattened about 16 days after SAH (*n* = 43 states) with the first peak date of 11 April 2020. Six out of 43 SAH states had the peak (or first peak Table 1 and Figure 1) before the date of 11 April 2020. The second rise of the curve followed 23 days after ending the SAH order during a phased society reopening and 8 days post Memorial Day. The trends increased following nation-wide gathering events during the months of May and June and the July 4th holiday. The most recent peak date, or the second peak date, if a state had already peaked around 11 April 2020, was 19 days post-July 4th, that is, on 23 July 2020, The overall averaged incidence rate was higher in the SAH + no-MFM states, while overall averaged CFR was significantly higher in the SAH + MFM states (Table 1).

The daily new total case curves (Figure 1B), daily average case curve (Figure 1C), and daily death curve (Figure 1D) have been shown to bend downward in the group of SAH + MFM states (*n* = 34) and were flattened in the group of SAH + no-MFM states (n = 9) during SAH periods. Normalized daily case data represented as daily new cases to the case number on day 1 of SAH (i.e., using the same dates as in the SAH groups) in the upper inset of Figure 1B, revealed a significant increase in the trend for new daily cases in the no-SAH + no-MFM states (*n* = 7). The SAH + MFM and SAH + no-MFM states showed 56.5% and 57.7% decreases, respectively, in daily infection trends when compared to no-SAH + no-MFM. There were similar daily infection trends between SAH + MFM and SAH + no-MFM states during the SAH period. Normalized daily death represented as daily new deaths to the death in day 1 of SAH in the inset of Figure 1D, showed a significant curve bending downward in the SAH + MFM states. This downward trend could indicate a 61.1% decrease in death numbers in the SAH + MFM states during SAH periods alone.

Thirteen out of 34 SAH + MFM states and two out of seven no-SAH + no-MFM states had two peaks. Zero out of nine SAH + no-MFM states displayed a second peak (*p* < 0.05 vs. SAH + MFM. Table 1) during the pandemic period. There was a significant difference in the number of days from the date of the first case in each state to the most recent peak between SAH + no-MFM states and no-SAH + no-MFM states (Table 1). Thus, these results indicated that SAH helped flatten the transmission and save lives.

### States with stay-at-home orders and mandatory face masks policies have mixed results regarding daily positive cases and fatality during the pandemic periods

**Figure 2:**
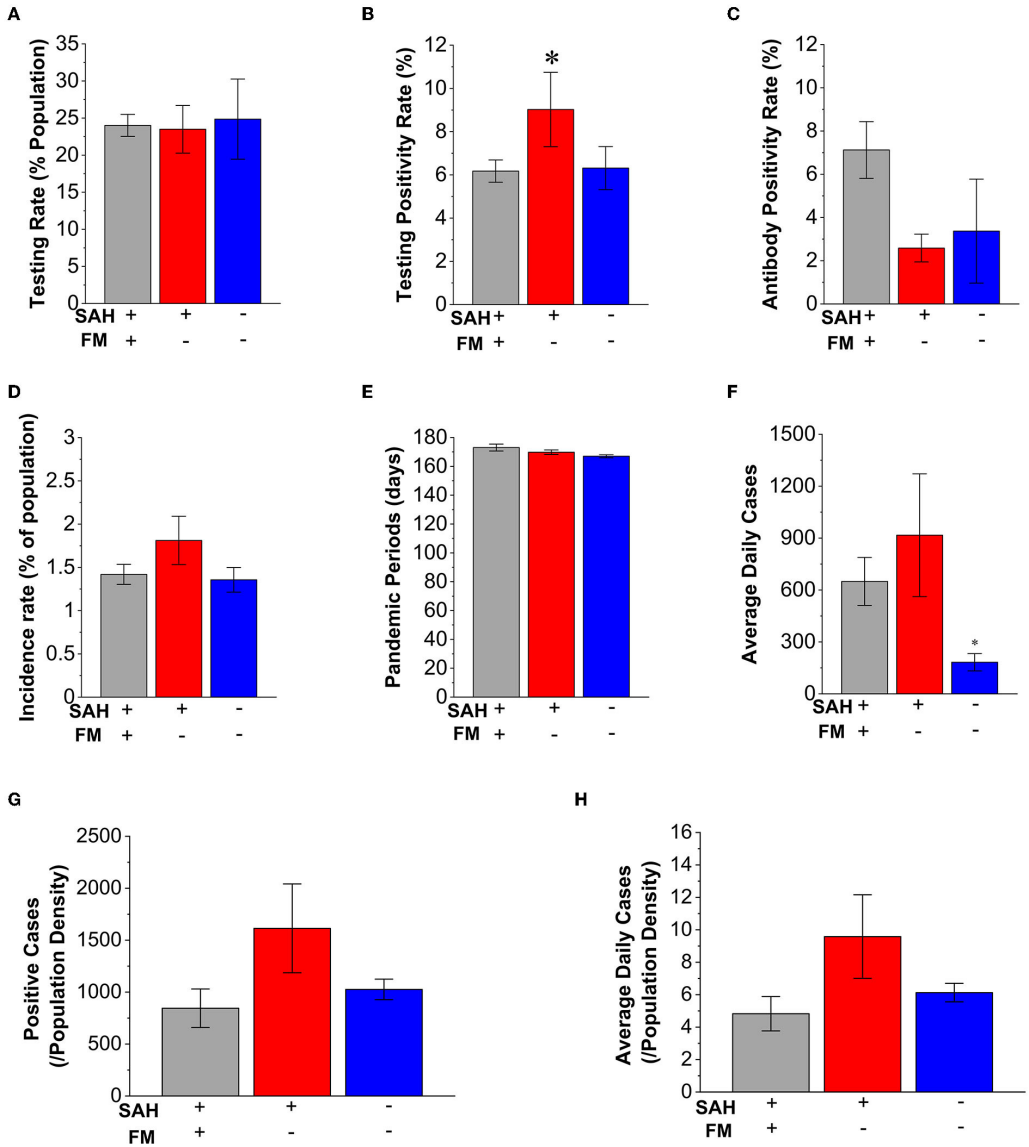
Characteristics of mandating face masks (MFMs) and stay-at-home (SAH) strategies in average daily cases and incidence rate of COVID-19. **(A)** There is no significant difference in the testing percentage of the state population among the SAH + MFM (*n* = 34), SAH + no-MFM (*n* = 9), and no-SAH and no-MFM (*n* = 7) states. **(B)** There is a significantly higher testing positivity rate in the SAH + no-MFM state when compared to SAH + MFM and no-SAH + no-MFM states. When compared to SAH + MFM, SAH + no-MFM is about 46% higher in positivity rate. There are no significant differences in antibody positivity rates **(C)**, incidence rate **(D)**, and periods of pandemic **(E)** among the three groups. **(F)** The average daily positive cases are significantly higher in the SAH + MFM and SAH + no-MFM states when compared to the no-SAH + no-MFM states. There is no significant difference in total positive cases reported **(G)** and in the average daily positive cases **(H)** per state population density during the pandemic among the SAH + MFM, SAH + no-MFM, and no-SAH + no-MFM states. FM: face mask. Each bar represents the mean ± SE. *p < 0.05 vs. SAH + MFM states.

To evaluate whether SAH and MFM are associated with the prevention of infection and severity of disease, we investigated the testing positivity rate, incidence rate, mortality rate, and CFR among each state group during the pandemic period (about 171 days until 23 August 2020). There was no significant difference in test percentage in the population of the states (24.01 ± 1.48% in SAH + MFM states, 23.49 ± 3.21% in SAH + no-MFM states, and 24.85 ± 5.40% in no-SAH + no-MFM states (Figure 2A). The SARS-CoV-2 PCR antigen testing positivity rate in SAH + no-MFM states was significantly higher compared to the positivity rate in the SAH + MFM states (Figure 2B) but was not significantly different compared to the positivity rate in the no-SAH + no-MFM states. To compare the COVID-19 infected level, the SARS-CoV-2 antibody positivity rate from available studies was also examined (32). The results indicated that there were no significant differences in antibody levels in the sampled population among the SAH + MFM (*n* = 31), SAH + no-MFM (*n* = 9), and no-SAH + no-MFM states (*n* = 7 Figure 2C), respectively.

There were no significant differences in the results of incidence rate among the SAH + MFM (1.42%), SAH + no-MFM states (1.81%), and no-SAH + no-MFM states (1.35%) and no significant difference in the dates of pandemic periods (Figures 2D,E). However, the SAH + MFM state has a significantly higher average of daily new cases compared to the no-SAH + no-MFM state (Figure 2F).

To examine whether total positive cases and average daily new cases were impacted by population density, we investigated total positive cases and the average daily positive cases when normalized with population density. A geographic analysis of population density during the pandemic indicated that population density could be a factor affecting transmission and fatality (33, 34). The information regarding state population density (residents/mile^2^) has also been considered during the analysis (Table 1). There was a significant difference in the population density in the groups of the SAH + MFM, SAH + no-MFM, and no-SAH + no-MFM states (Table 1). The SAH + MFM states have a much higher population density when including Washington DC. There were no differences between total positive cases and the average daily positive cases when normalized with population density among the three groups (Figures 2G,H). These results suggested that SAH and MFM/no-MFM orders provided mixed effects on the prevention of infection.

**Figure 3:**
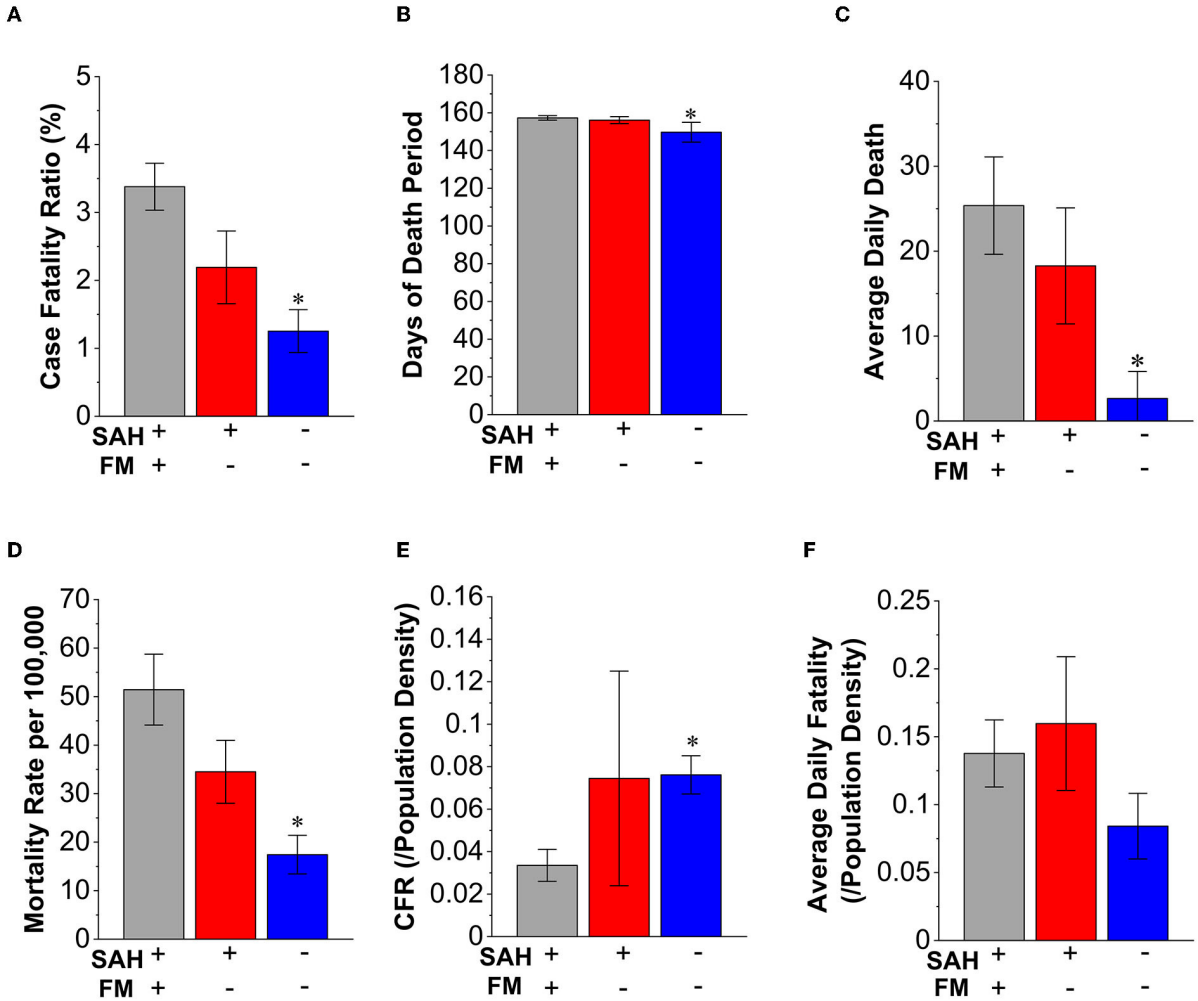
Characteristics of mandating face masks and stay-at-home strategies in the fatality of COVID-19. **(A)** The case-fatality ratio (CFR) is higher in the SAH + MFM (2.7-fold, *p* < 0.05, *n* = 34) and SAH + no-MFM (1.7-fold, *n* = 9) states when compared to no-SAH + no-MFM (n = 7) states. **(B)** There are about 5% fewer days when counting fatality in the no-SAH + no-MFM than in the SAH + MFM states. There are no significant differences in days of fatality periods between the SAH + no-MFM and no-SAH + no-MFM states. **(C)** The average daily death number in the no-SAH + no-MFM states has significantly fewer death than the SAH + MFM and SAH + no-MFM states. **(D)** The mortality rates per 100,000 population of the SAH + MFM and SAH + no-MFM states are higher than mortality in the no-SAH + no-MFM states. **(E)** The CFR per state population density is significantly higher in the no-SAH + no-MFM when compared to the SAH + MFM states. No significant difference in CFR per population density between the SAH + no-MFM states and the SAH + MFM states. **(F)** There is no significant difference in the average daily death cases reported per state population density during pandemic among the SAH + MFM, SAH + no-MFM, and no-SAH + no-MFM states. Each bar represents the mean ± SE. *p < 0.05 vs. SAH + MFM states.

To examine whether fatality is impacted by population density, we investigated CFR and the average daily death cases when normalized with population density. When comparing case severity levels without normalizing to the population density, states with SAH + MFM had a 2.7-fold higher CFR (3.39%) compared to no-SAH + no-MFM states (1.25%. *p* = 0.009 Figure 3A). The fatality periods in states with no-SAH + no-MFM have 4.8% fewer days compared to SAH + MFM and SAH + no-MFM (*p* = 0.017. Figure 3B). Because longer fatality periods could result in greater death numbers, we also compared daily deaths among three groups. The states with SAH + MFM or SAH + no-MFM had 9.53-fold (*p* < 0.05) or 6.94-fold higher averages of daily death than no-SAH + no-MFM states, respectively. When total deaths were normalized with the population (per 100,000 residents) as mortality rates, the SAH + MFM (3.0-fold, *p* < 0.05) and SAH + no-MFM (2.0-fold) states had higher mortality rates than the no-SAH + no-MFM states (Figure 3D). When CFR was normalized by population density, the no-SAH + no-MFM states had a significantly higher CFR when compared to SAH + MFM states (p = 0.033. Figure 3E). However, there was no significant difference in average daily fatality per population density among the three groups (Figure 3F). These results suggested that SAH and MFM/no-MFM orders provided mixed outcomes for protection on the severity of the disease.

The state of Arkansas (22.2% testing percentage) issued a MFM order without a SAH order (as the fourth group, only one state) and was excluded from the three state groups above. Varied results indicated an 8.4% antigen testing positivity rate and a 1.87% incidence rate, which were similar to SAH + no-MFM states, and a 1.20% CFR that was similar to no-SAH + no-MFM states. In addition, when comparing MFM states (*n* = 34) with all non-MFM (*n* = 16) without considering SAH, there were no significant differences in the incidence rate and CFR (data not shown).

### States with MFM policies have not shown preventive effects on the incidence or average daily cases during mandatory SAH order periods

**Figure 4:**
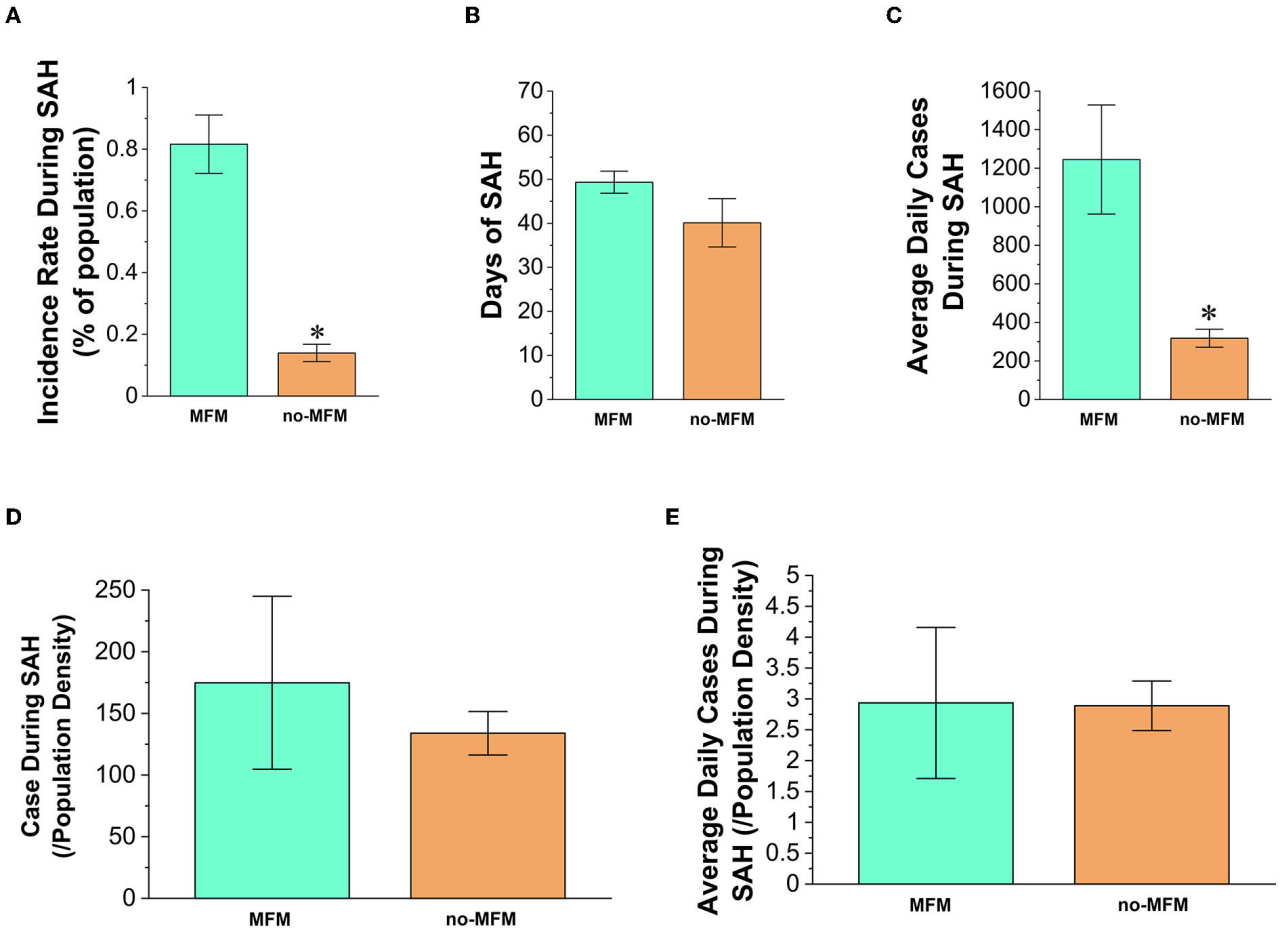
Characteristics of states overlapping with mandating masks in the transmission of COVID-19 during the periods of a mandatory stay-at-home (SAH) order. **(A)** The infected cases in no-MFM states (*n* = 31) have a significantly lower incidence rate than the overlapping MFM states (*n* = 12) with SAH order. **(B)** There is no significant difference in the SAH period between the no-MFM and MFM states. **(C)** The average daily positive cases in no-MFM states showed significantly fewer numbers than the MFM states. There are no significant differences in total positive cases **(D)** and average daily cases **(E)** per state population density between the MFM and no-MFM states. Each bar represents the mean ± SE. *p < 0.05 vs. MFM states.

To examine whether SAH overlapping with MFM order (yellow rectangle box in the lower part of Figure 1A) demonstrated improved preventive effects, we examined the incidence rate along with daily new cases during the SAH window (averaged about 45 days. *n* = 43 states). The average overlapping period for SAH and MFM was 28 ± 3 days, which was about 50% of overall SAH days. The incidence rate in MFM states (*n* = 12) was significantly higher than that in no-MFM states (*n* = 31. *p* = 0.003. Figure 4A) during SAH periods. Because the number of SAH days in MFM states was 23% longer than in no-MFM states (Figure 4B), which could increase the positive cases data, we examined the average daily new cases between the two groups during the SAH periods. The average daily new positive cases in MFM states was 4-fold higher than the no-MFM states (*p* = 0.004 Figure 4C).

We examined total positive cases and average daily new cases in relation to population density. There were no significant differences in the number of total positive cases (Figure 4D) and average daily cases (Figure 4E) impacted by population density between MFM and no-MFM states. Thus, these results suggested that the MFM policy during the SAH period may not have had an advantage over the no-MFM policy.

## Discussion

This study is the first report to evaluate all available COVID-19 positive cases and fatality data in the US, without utilizing theoretical modeling or selected regional databases, from the policy-making levels during the pandemic periods until 23 August 2020. The key strengths of this article include that there were no specific drugs or vaccines that were participated or finished in large scale phase III clinical trials prior to August 2020 with the original SARS-CoV-2 strain (reference in vaccine and treatment information section in Supplementary eTable 1). According to the health department reporting system, patients were only counted one time for incidence during the early phase of the pandemic. This pandemic marks the first time in history that diagnostic testing is provided for free to the general public without a physician's order. The free test was included in all hospital admission and death coding procedures. Our separated four-state groups were based on their own policies under CDC and FDA guidelines (no researcher selection bias) and were unique, that is, one country including four possible combinations of SAH and MFM policies. Thus, the analysis of SAH and MFM effectiveness in each individual group will have fewer confounding variables than when comparing different countries, as these four groups are a part of the same nation with national standardized healthcare professional practice and hospital systems.

Our results were inconsistent with the intent of state-level public health strategies in lowering overall infection rates and fatality. This study provided direct evidence of potential decreased infection and fatality in SAH + MFM and SAH + no-MFM states during SAH periods (Figure 1). There were also potential decreases in testing the positivity rate when comparing the SAH + MFM to SAH + no-MFM groups and a decrease of fatality rate to potentially save lives when normalized by population density through strategies of the SAH + MFM orders during the COVID-19 pandemic (Figure 3). However, the no-SAH + no-MFM group had a lower incidence rate, average daily news cases, CFR, and mortality rates (Figures 2, 3 and Table 1). There is no significant difference in SARS-CoV-2 antigen and antibody positivity rates among the three groups. When normalized to population density, there were no significant differences in total positive cases, averaged daily new cases, and average daily fatalities among the SAH + MFM, SAH + no-MFM, and no-SAH + no-MFM state groups during pandemic periods. In addition, when normalized to population density, there were no differences in total positive cases and average daily new cases between the MFM and no-MFM groups during the SAH period.

Pathogenesis is the process by which an infection leads to either asymptomatic or symptomatic signs of that disease. Theoretically, this process is always a balance between viral invasion (i.e., enough quality-virulence factors, quantity-virus load, and contact time with contagious source-15 min as the CDC estimated) and human defense (i.e., physical and mental health). Before vaccines and specific drugs are available, what we can do from a prevention standpoint is to hinder the pathogen from reaching the sufficient virus load by using non-pharmaceutical physical barriers such as face coverings and hand hygiene, and to reduce contact time through the isolation of patients, SAH, social distancing and decreasing exposure to unknown crowds (6, 7, 35–37). Prolonged time in crowded indoor spaces (e.g., bars and restaurants) will increase your contact time with a possible contagious source. Human defense is the selection of a healthy lifestyle to boost an individual's immune system to fight virulent factors, while receiving medical care in time if deemed appropriate. Available COVID-19 vaccines with FDA Emergency Use Authorization (EUA) or approval contain viral DNA, mRNA, and proteins, and can help boost the human immune defense system (preventive and protective efforts) by the end of the year 2020 (38). However, people who are vaccinated may still contract COVID-19 from immune escape, especially with coronavirus mutations/variants that are referred to as breakthrough infection (CDC4 and 5 in Supplementary eTable 1) (39, 40). It is recognized that breakthrough cases who are vaccinated are much less likely to experience severe illness and hospitalization than unvaccinated people. Around the US, federal and state governments have been fighting the COVID-19 pandemic through a variety of strategies with the assistance of *predictive* modeling. These strategies include testing to find patients, isolating patients (*preemptive*), controlling transmission (*preventive*), and finally providing necessary clinical care when needed (*protective* and *personal care*).

The first step is to find the contagious source—the patients. Free testing is critical in the public health field's mitigation efforts because it helps investigators to characterize the incidence rate, spread, and contagiousness of the disease. Early testing helps identify anyone who encounters an infected person so they too can be quickly quarantined, isolated, and then treated if needed. The sooner patients receive their test results, the sooner infected individuals can be isolated before transmitting their infection to others. Among all three with or without SAH and MFM states, the testing percentages, SARS-CoV-2 antigen, and antibody positivity rates, and total COVID-19 positive cases per population density had no significant differences during the pandemic period. However, the disadvantages of unjustified testing include potentially wasting limited amounts of resources and delaying in the lab results for actual patients.

The second step of this strategy is the mandatory isolation of patients and voluntary quarantine of close contactors followed by contact tracing and free society support services, etc. Health care, including public health and medicine, is both a science and an art. For communicable diseases, no contact will mean no contagion (Science aspect). This is the most important preemptive step because better control of a contagious source will greatly reduce transmission in the community, thereby reducing the positivity rate, incidence rate, and fatality in the community (41, 42). It has been reported that about 30–62% of patients were only partially isolated or not isolated at all (43). If self-isolating or self-quarantining (Science aspect) at home is not possible, local governments or communities could provide designated places with free living support and rewards for close contacts and positive cases whose vital signs are stable (Art aspect). In addition, one big shortcoming of the US' response to the coronavirus pandemic, at least in some parts of the country, involves a shortage of contact tracers and effectiveness of contact tracing (44–47). When new daily cases reach a certain level (such as 200/day in Houston Area, TX), it is very difficult to effectively trace and help isolate the further spread of the virus (TMC in Supplementary eTable 1).

The third step of this strategy is preventive transmission control through the CDC recommendations of hand washing often, maintaining good social distancing, wearing a face covering when around others if not vaccinated, and monitoring your health daily (self-awareness). These NPIs were very important preventive parts in the early pandemic when no vaccines and drugs were available. During influenza-like-illness season, including the COVID-19 outbreak before April 2020, the CDC and World Health Organization (WHO) did not recommend face masks to the general public because there was no evidence from clinical trials of their effectiveness in reducing transmission (8, 13). However, the CDC recommends using face masks in public if you are sick, taking care of a sick patient, have underlying conditions such as immunocompromised persons or have to be in a crowded environment. As COVID-19 continues its global spread, the WHO and CDC recommended that it was possible these strategies in pandemic control, such as face masks, might at least help reduce the severity of the disease and ensure that a greater proportion of new positive cases are asymptomatic infections (37, 48, 49). Furthermore, rejecting a low-cost intervention such as mass masking as ineffective, because there is no evidence of efficacy in clinical trials, is potentially detrimental (50). Our data indicated that SAH and MFM in the general public could help with mitigation, and even save lives during the COVID-19 pandemic as shown in the part of our data in Figures 1–3 with population density, and in the balance of pathogenesis as mentioned above (22, 51). Several studies found a reduction in the spread of COVID-19 after stay-at-home, school closure, and social distancing mandates were enacted in most cities and states (15–19, 52, 53). The national overall daily new cases curve was consistent with these reports. There were several significant cause-and-effect relationships in the daily case trend curve (Figure 1). The daily new case and death curve trends were bent downward, indicating decreased transmission and fatality, following a mandated short-term SAH order (weeks. Average 45 days). However, it is possible that the economic and non-economic consequences of the long-term SAH orders outweigh the need to protect public health (27, 54–57). When US states began phased reopening of their economies, and communities started gathering, the daily new cases observed an upward surge. This suggested the efficacy of short-term SAH and social distancing measures which could influence future public health policy-making (18, 52). However, the pandemic results indicated that there was no significant difference among the three groups in positivity rates and incidence rates during the pandemic periods.

The impact of population density on emerging highly contagious infectious diseases has rarely been studied. In theory, dense areas over a certain threshold level could lead to more close interactions among residents, which makes them potential hotspots for the rapid spread of diseases. Moreover, dense cities may have better access to healthcare systems (33, 34). Our research design for the current COVID-19 pandemic is an apt population-based cross-sectional study to investigate these relationships. Our data indicated that the top eight states in population densities are in the SAH + MFM group with high average daily new cases, mortality, CFR, and average daily fatality. However, after being normalized with population density, there were no significant differences in daily new cases and average daily fatality among the three groups during the pandemic periods (Figures 2, 3), as well as between MFM and no-MFM groups during SAH periods (Figure 4). Many results in this study were inconsistent with the goals of preventive protocol, including SAH and MFM, to combat the pandemic and to reduce both infection rate and severity of the disease. A limitation of this study was its focus on the difference in state-level policies. To find out why SAH and MFM orders did not show significant changes in infection and fatality, we may need analysis of individual county or city socioeconomic data, patient isolating data, contact tracing data, healthcare systems data, and law enforcement efforts during these mandating requirements (see limitations section).

Recent studies indicated that not all face masks have equal efficacy in reducing the transmission of particles or droplets, those most likely involved in COVID-19 people-to-people transmission (3, 5). Among all laboratory-tested masks, fitted N95 with other necessary PPE, used by health professionals who take care of patients with COVID-19, performed the best (Science aspect). Three-layer surgical masks, used by professionals in the hospital and clinical settings, also showed preferable results. Some mask alternatives, such as neck fleeces or bandanas, offer very little protection (3, 58). From a public policy perspective, during a pandemic like the COVID-19 pandemic, shortages in the supply of surgical face masks and N95 respirators, as well as concerns about side effects and the discomfort of prolonged use of the masks, have led to the public use of a variety of solutions which are generally less restrictive and usually of unknown efficacy (Supplementary eTable 5). When healthcare workers encounter patients with COVID-19, based on CDC recommendations, they normally need PPEs including N95 after fit-test, gloves, room with negative pressure, and even head-to-toe isolation gowns. It has been reported that because of the shortage of PPEs during the early phase of the pandemic, healthcare workers had a higher risk of infection of COIVD-19 before September 2020 (59, 60). In our opinion, if contagious patients do not strictly isolate themselves in the designated places and stay away from the rest of the community, and if the general public is not informed of the correct types of masks (e.g., N95. Science aspect) with free of charge at all entrances (Art aspect), proper methods to wear a mask, and willing to properly social distance (3, 5), the community transmission will most likely be inevitable. The similar positivity rates of SARS-CoV-2 antigen and antibody, and incidence rate among three groups indicated compromised effectiveness of MFM and/or SAH strategies during the pandemic periods (61).

**Figure 5:**
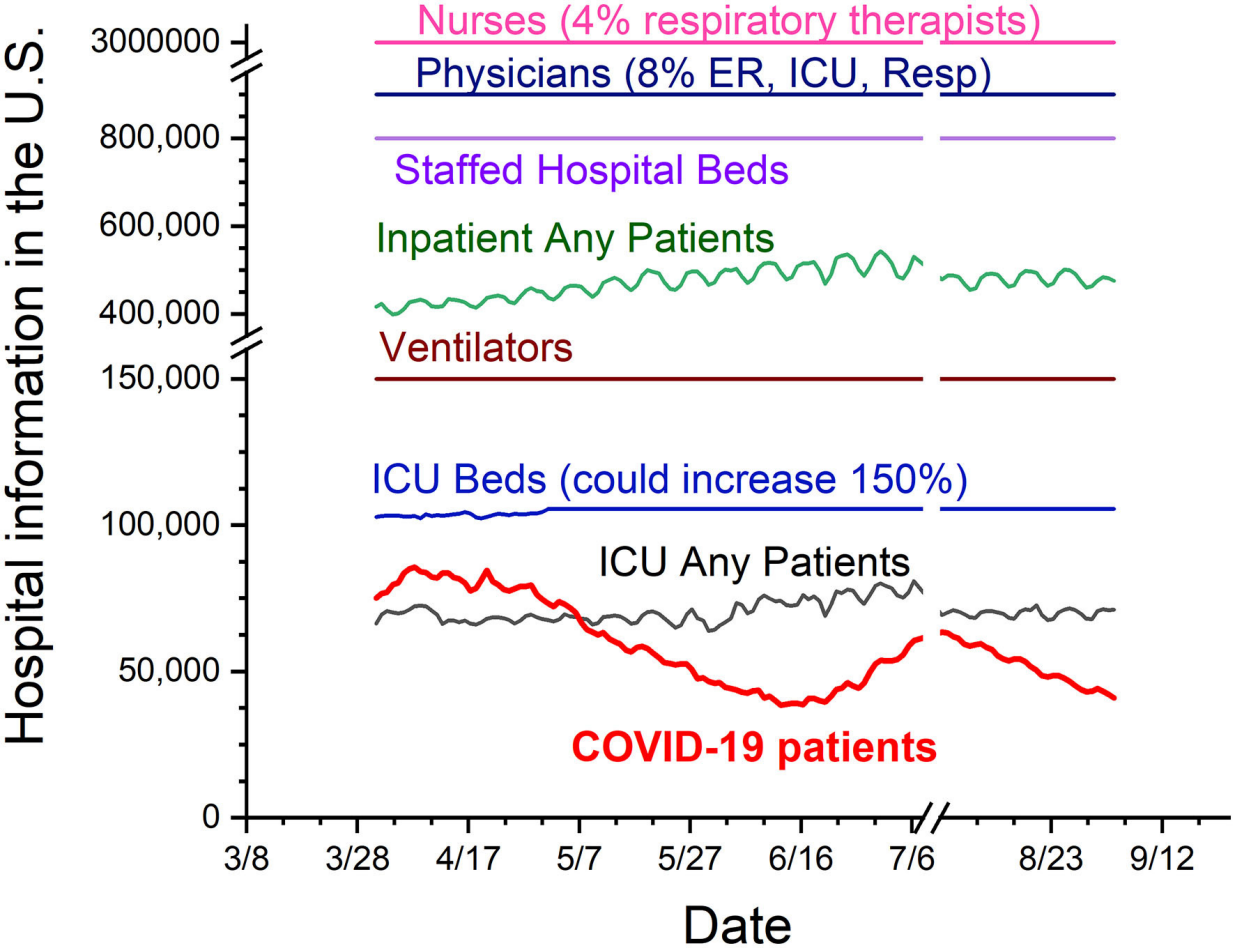
US hospital capacity during the COVID-19 pandemic. The periods are from the date 1 April 2020 to 4 September 2020. The chart includes numbers of physicians, nurses, hospital beds, all hospitalized patients, ventilators, intensive care unit (ICU) beds, all ICU patients, and all patients with COVID-19. From the numbers in this chart, it is possible that individual hospitals might have the shortage of beds or respiratory therapists. However, there are sufficient hospital capacities for patients with COVID-19 during the pandemic period before the September 2020.

The fourth and last step of this strategy is to provide protective clinical care and prevent overwhelming the healthcare system during the pandemic. Hospitals and other healthcare facilities play a critical role in national and local responses to COVID-19. Until June 2022, three vaccines and three antiviral treatments have been approved (or EUA) with debatable benefit-risk assessment by FDA (62–64). However, the lone healthcare strategies were only supportive care solutions and the ability to manage underlying conditions (protective and personalized care) before September 2020. When mitigation steps are compromised, as our data from the COVID-19 pandemic has indicated, the final step is to ensure that we have sufficient healthcare capacity, especially the capacity of the intensive care unit (ICU). Patients with COVID-19 need rooms with negative pressure (e.g., ICU) to prevent contamination to the outside and need ventilators because of the respiratory issues associated with the disease. How do we define the hospital capacity with the society safety threshold and tightening threshold needed to handle a potential outbreak demand during phased reopening (52)? The CDC does not have clear recommendations yet. In Texas, if patients with COVID-19 made up <15% of all hospitalizations, an increase in reopening capacity every 14 days was permitted (Texas reopening in Supplementary eTable 1) before the year 2021. The hospital capacity data in Figure 5 indicate that there was sufficient healthcare capacity to take care of patients with COVID-19 in most of the nation based on Texas's 15% suggestion before October 2020. In addition, there are 900,000 active licensed physicians including at least 70,000 physicians specializing in the emergency room, intensive care unit, and respiration (29, 65). Moreover, there are approximately 3 million nurses and 129,000 respiratory therapists available in the US (Figure 5). The mitigation steps might be appropriately tightened or loosened to minimize social and economic disruption following the balance among patient's privacy, vulnerable populations' safety, people's freedom, and law enforcement following hospital capacity changes.

## Limitations

This study has several limitations that other public health studies might also be faced when studies involve all populations. First, these results were limited to the state policy levels under one CDC guideline. We did not consider data on state-wide school closure, non-essential business closure, and county-level MFM orders in no-MFM states, such as those in the state of Florida. Second, we only examined confirmed COVID-19 provisional cases and reported death cases until 23 August 2020. The reported cases and fatality data can lag. There is evidence by the CDC of a higher infection rate in the community than what is reflected in the number of confirmed COVID-19 cases, especially in asymptomatic patients. In addition, we did not examine the percentage of patients who died with the do-not-resuscitate status that could be responsible for increased mortality. Third, we did not examine covariates (possible confounders), such as socioeconomic and demographic factors (e.g., age, sex, and education level), enforcement of the mandates, percentage of comorbidity population in nursing homes and assisted living facilities between states. Those with higher proportions of minority residents are more likely to have more COVID-19 cases (66, 67).

## Conclusion

This study is the fact of the COVID-19 pandemic in a real-world population that any so-called successful modeling studies and controlled clinical trials will be challenged when translating into practice. The results provided direct evidence of potential decreases in infection and fatality with short-term SAH order (Figure 1). There was a decrease in fatality rate (more saved lives) when normalized to population density through strategies of SAH + MFM orders. However, overall, many results in this study were inconsistent with the intent of state-level public health strategies of short-term SAH and long-term MFM orders in lowering transmission and fatality. As states began to reopen following October 2020, there were multiple huge upward waves of COVID-19 cases even though three more states enforced MFM policies from November 2020 to January 2021, and the vaccinated population increased and multiple anti–COVID-19 drugs were approved through August 2022 (CDC1 in Supplementary eTable 1). From the policy-making level, if contagious patients would not likely be placed in strict isolation and massive contact tracing would not be effective to implement, we presume that following the CDC recommendations with close monitoring of healthcare capacity, should no individual state-level policies be required, could be appropriate in helping mitigate the COVID-19 disaster and limiting collateral socioeconomic damage. Mass vaccination to reach herd immunity, along with other public health measures could be one of the most efficient ways to diminish the pandemic (68–72). With the world facing an unprecedented threat, we must learn the lessons of this pandemic now and ensure that our response is based on vulnerable patients, people's freedom, community risk, and hospital capacity to make the world a safer place in potential future pandemics.

## Data availability statement

The original contributions presented in the study are included in the article/Supplementary material, further inquiries can be directed to the corresponding author.

## Author contributions

SW and XW conceived the study, collected the data, searched the literature, wrote the manuscript, and analyzed the data. Both authors were involved in data interpretation and made meaningful contributions to the final submitted manuscript.

## Funding

This research was partly supported by institutional fund from the Texas A&M Health Science Center.

## Conflict of interest

The authors declare that the research was conducted in the absence of any commercial or financial relationships that could be construed as a potential conflict of interest.

## Publisher's note

All claims expressed in this article are solely those of the authors and do not necessarily represent those of their affiliated organizations, or those of the publisher, the editors and the reviewers. Any product that may be evaluated in this article, or claim that may be made by its manufacturer, is not guaranteed or endorsed by the publisher.
