# Supplementary material for: Stay-at-home and face mask policy intentions inconsistent with incidence and fatality during the US COVID-19 pandemic
Source: Front Public Health. 2022 Oct 13;10:990400. doi: 10.3389/fpubh.2022.990400 (PMC9609417; doi:10.3389/fpubh.2022.990400)
Supplement: Supplementary file 1 [file Presentation_1.pdf]

Supplementary Online Content

Stay-at-home and face mask policies intentions inconsistent with incidence and fatality during the US COVID-19 pandemic

Samuel Xi Wu and Xin Wu, MD, MS

eTable 1. Public reports reference other than papers and books Pages 2-3

eTable 2. Date of stay at home and mandatory face mask orders Page 4

eTable 3. COVID-19 cases until 2020-08-23 Pages 5-15

eTable 4. COVID-19 death until 2020-08-23 Pages 16-26

eTable 5. Facts behind face mask: cost and effectiveness Page 27

Supplemental

Supplemental eTable 1: Public reports reference other than papers and books*

**Case and fatality data:**<br>(1) CDC1: COVID-19a and COVID Data Tracker: https://www.cdc.gov/coronavirus/2019-nCoV/index.html<br>(2) CDC2. United States COVID-19 Cases and Deaths by State over Time: https://data.cdc.gov/Case-Surveillance/United-States-COVID-19-Cases-and-Deaths-by-State-o/9mfq-cb36<br>(3) CDC3: Considerations for wearing cloth face coverings : help slow the spread of COVID-19 https://stacks.cdc.gov/view/cdc/90553<br>(4) U.S. Department of Health and Human Services Office of the Chief Data Officer. COVID-19 State and County Policy Orders: https://healthdata.gov/dataset/covid-19-state-and-county-policy-orders<br>(5) Johns Hopkins University & Medicine. Coronavirus Resource Center: https://coronavirus.jhu.edu/us-map The Covid-19 Tracking Project. The Data: https://covidtracking.com/data#TX<br>(6) Our World in Data. Coronavirus Pandemic (COVID-19) : https://ourworldindata.org/coronavirus#explore-the-global-situation
**Hospital and health professional data:**<br>(7) American Hospital Association. Fast Facts on U.S. Hospitals, 2022 : https://www.aha.org/statistics/fast-facts-us-hospitals<br>(8) American Nurses Association. Nurse workforce in the US: https://www.nursingworld.org/practice-policy/workforce/<br>(9) Bureau of Labor Statistics. Respiratory Therapists: https://www.bls.gov/ooh/healthcare/respiratory-therapists.htm<br>(10) Association of American Medical Colleges. physician data: Physician Specialty Data Report: https://www.aamc.org/data-reports/workforce/interactive-data/active-physicians-us-doctor-medicine-us-md-degree-specialty-2019
**Stay at home and face masking order:**<br>(11) USA Today: Coronavirus reopening. Map of COVID-19 case trends, reopening status and mobility, 20200829: https://www.usatoday.com/storytelling/coronavirus-reopening-america-map/<br>(12) The New York Times. See Which States and Cities Have Told Residents to Stay at Home: https://www.nytimes.com/interactive/2020/us/coronavirus-stay-at-home-order.html<br>(13) AARP. Face mask: State-by-State Guide to Face Mask Requirements. https://www.aarp.org/health/healthy-living/info-2020/states-mask-mandates-coronavirus.html<br>(14) #Masks4All, a fiscally sponsored project of Community Initiatives. Face masks: What U.S. States Require Masks In Public? https://masks4all.co/what-states-require-masks/
(15) Population density:<br>**Wikipedia:** based on data from the 2013 estimate of population by the United States Census Bureau: https://simple.wikipedia.org/wiki/List_of_U.S._states_by_population_density
(16) **Texas reopening:** Gov. Greg Abbott loosens coronavirus restrictions for restaurants and other businesses in most regions of Texas: https://www.texastribune.org/2020/09/17/greg-abbott-texas-coronavirus/
(17) Three Metrics To Gauge Our Progress, Coronavirus (COVID-19) Updates. Texas Medical Center **(TMC)**. https://www.tmc.edu/coronavirus-updates/three-metrics-to-gauge-our-progress-2/

Vaccine and treatment information

(18) CDC4, COVID-19 after Vaccination: Possible Breakthrough Infection: https://www.cdc.gov/coronavirus/2019-ncov/vaccines/effectiveness/why-measure-effectiveness/breakthrough-cases.html

(19) CDC5, COVID-19 Treatments and Medications: https://www.cdc.gov/coronavirus/2019-ncov/your-health/treatments-for-severe-illness.html

(20) Pfizer and BioNTech Conclude Phase 3 Study of COVID-19 Vaccine Candidate, Meeting All Primary Efficacy Endpoints: https://www.pfizer.com/news/press-release/press-release-detail/pfizer-and-biontech-conclude-phase-3-study-covid-19-vaccine#:~:text=The%20Phase%203%20clinical%20trial,as%20of%20November%2013%2C%202020.

(21) Phase 3 clinical trial of investigational vaccine for COVID-19 begins (July 27, 2020, NIH News Releases). https://www.nih.gov/news-events/news-releases/phase-3-clinical-trial-investigational-vaccine-covid-19-begins

(22) FDA NEWS RELEASE, FDA Approves First Treatment for COVID-19 (October 22, 2020). https://www.fda.gov/news-events/press-announcements/fda-approves-first-treatment-covid-19

(23) Gundlapalli AV, Lavery AM, Boehmer TK, et al. Death Certificate–Based ICD-10 Diagnosis Codes for COVID-19 Mortality Surveillance — United States, January–December 2020. CDC Weekly, April 9, 2021, 70(14);523–527. https://www.cdc.gov/mmwr/volumes/70/wr/mm7014e2.htm

*if web link no longer working, search it with article title.

eTable 2. Data of date of stay at home and mandatory face mask orders

States	population in 2019	population density 2013 (/mile2)	current date	date of mask order	stay at home starting date	stay at home ending date	COVID-19 antigen testing number until 8/23/2020	Date of the first peak of the new daily positive (7 day average)	date of most recent peak of the new daily positive (7 day average)
.Alabama	4,903,185	95.40	8/23/2020	7/15/2020	4/4/2020	4/30/2020	916,703	7/24/2020	7/24/2020
.Alaska	731,545	1.30	8/23/2020		3/11/2020	4/21/2020	327,440	8/1/2020	8/1/2020
.Arizona	7,278,717	58.30	8/23/2020		3/30/2020	5/15/2020	1,149,287	6/1/2020	6/1/2020
.Arkansas	3,017,804	57.00	8/23/2020	7/20/2020			670,826	7/24/2020	7/24/2020
.California	39,512,223	246.10	8/23/2020	6/18/2020	3/19/2020	5/4/2020	10,541,031	7/29/2020	7/29/2020
.Colorado (2 peaks)	5,758,736	50.80	8/23/2020	7/17/2020	3/26/2020	5/8/2020	930,152	4/25/2020	7/26/2020
.Connecticut	3,565,287	742.60	8/23/2020	4/20/2020	3/23/2020	5/20/2020	1,020,328	4/21/2020	4/21/2020
.Delaware (2 peaks)	973,764	475.10	8/23/2020	4/28/2020	3/24/2020	5/15/2020	221,845	4/27/2020	8/14/2020
.District of Columbia (2 peaks)	705,749	10588.00	8/23/2020	5/16/2020	4/1/2020	5/29/2020	266,046	5/2/2020	7/22/2020
.Florida	21,477,737	364.60	8/23/2020		3/20/2020	4/30/2020	6,016,043	7/12/2020	7/12/2020
.Georgia	10,617,423	173.70	8/23/2020		4/3/2020	4/30/2020	2,171,884	7/24/2020	7/24/2020
.Hawaii	1,415,872	218.60	8/23/2020	4/20/2020	3/25/2020	5/31/2020	228,381	8/14/2020	8/14/2020
.Idaho	1,787,065	19.50	8/23/2020		3/25/2020	4/30/2020	235,255	7/16/2020	7/16/2020
.Illinois (2 peaks)	12,671,821	232.00	8/23/2020	5/1/2020	3/21/2020	5/30/2020	3,704,036	5/13/2020	8/21/2020
.Indiana (2 peaks)	6,732,219	183.40	8/23/2020	7/27/2020	3/25/2020	5/1/2020	1,273,212	4/26/2020	8/23/2020
.Iowa	3,155,070	55.30	8/23/2020				595,765	8/23/2020	8/23/2020
.Kansas	2,913,314	35.40	8/23/2020	7/3/2020	3/30/2020	5/3/2020	366,315	8/23/2020	8/23/2020
.Kentucky	4,467,673	111.30	8/23/2020	5/11/2020	3/26/2020	5/11/2020	1,763,838	8/13/2020	8/13/2020
.Louisiana (2 peaks)	4,648,794	107.10	8/23/2020	7/13/2020	3/23/2020	5/14/2020	1,763,838	4/3/2020	8/3/2020
.Maine	1,344,212	43.10	8/23/2020	5/1/2020	4/2/2020	5/31/2020	231,256	5/21/2020	5/21/2020
.Maryland (2 peaks)	6,045,680	610.80	8/23/2020	4/18/2020	3/30/2020	5/15/2020	1,772,656	5/20/2020	8/1/2020
.Massachusetts	6,892,503	858.00	8/23/2020	5/6/2020	4/24/2020	5/18/2020	2,064,529	4/24/2020	4/24/2020
.Michigan (2 peaks)	9,986,857	175.00	8/23/2020	7/10/2020	3/24/2020	6/5/2020	2,564,334	4/2/2020	8/1/2020
.Minnesota (2 peaks)	5,639,632	68.10	8/23/2020	7/25/2020	3/27/2020	5/4/2020	1,374,536	5/23/2020	7/26/2020
.Mississippi	2,976,149	63.70	8/23/2020	8/4/2020	4/3/2020	5/11/2020	530,974	7/29/2020	7/29/2020
.Missouri	6,137,428	87.90	8/23/2020		4/6/2020	5/3/2020	1,218,097	7/29/2020	7/29/2020
.Montana	1,068,778	7.00	8/23/2020	7/16/2020	3/26/2020	4/24/2020	210,749	7/30/2020	7/30/2020
.Nebraska (2 peaks)	1,934,408	24.30	8/23/2020				338,836	4/30/2020	7/31/2020
.Nevada	3,080,156	25.40	8/23/2020	6/24/2020	3/31/2020	5/15/2020	809,683	7/17/2020	7/17/2020
.New Hampshire	1,359,711	147.80	8/23/2020		3/27/2020	6/15/2020	301,316	5/2/2020	5/2/2020
.New Jersey	8,882,190	1210.10	8/23/2020	4/8/2020	3/21/2020	6/15/2020	2,648,740	4/4/2020	4/4/2020
.New Mexico	2,096,829	17.20	8/23/2020	5/16/2020	3/24/2020	5/15/2020	704,955	7/28/2020	7/28/2020
.New York	19,453,561	417.00	8/23/2020	4/17/2020	3/22/2020	5/15/2020	7,621,159	4/9/2020	4/9/2020
.North Carolina (2 peaks)	10,488,084	202.60	8/23/2020	6/26/2020	3/30/2020	5/8/2020	2,078,472	7/23/2020	8/21/2020
.North Dakota	762,062	10.50	8/23/2020				432,725	8/23/2020	8/23/2020
.Ohio	11,689,100	283.20	8/23/2020	7/23/2020	3/23/2020	5/30/2020	1,977,822	7/30/2020	7/30/2020
.Oklahoma	3,956,971	56.10	8/23/2020				821,289	7/27/2020	7/27/2020
.Oregon	4,217,737	40.90	8/23/2020	7/1/2020	3/23/2020	5/15/2020	510,056	7/24/2020	7/24/2020
.Pennsylvania (2 peaks)	12,801,989	285.50	8/23/2020	4/19/2020	3/30/2020	5/15/2020	1,549,379	4/9/2020	7/28/2020
.Rhode Island (2 peaks)	1,059,361	1017.10	8/23/2020	5/8/2020	3/28/2020	5/8/2020	470,162	4/24/2020	8/6/2020
.South Carolina	5,148,714	158.80	8/23/2020		4/7/2020	5/12/2020	891,006	7/11/2020	7/11/2020
.South Dakota (2 peaks)	884,659	11.50	8/23/2020				176,843	5/9/2020	8/23/2020
.Tennessee	6,829,174	157.50	8/23/2020		4/2/2020	4/30/2020	2,042,256	7/27/2020	7/27/2020
.Texas	28,995,881	101.20	8/23/2020	7/3/2020	4/2/2020	4/30/2020	4,668,028	7/16/2020	7/16/2020
.Utah	3,205,958	35.30	8/23/2020				766,985	7/17/2020	7/17/2020
.Vermont	623,989	68.00	8/23/2020	8/1/2020	3/24/2020	5/15/2020	120,043	4/4/2020	4/4/2020
.Virginia (2 peaks)	8,535,519	209.20	8/23/2020	5/29/2020	3/30/2020	6/10/2020	1,466,033	5/26/2020	8/8/2020
.Washington	7,614,893	104.90	8/23/2020	6/26/2020	3/23/2020	5/4/2020	1,379,036	7/13/2020	7/13/2020
.West Virginia	1,792,147	77.10	8/23/2020	6/6/2020	3/24/2020	5/4/2020	396,018	7/24/2020	7/24/2020
.Wisconsin	5,822,434	106.00	8/23/2020	8/1/2020	3/25/2020	5/26/2020	1,185,611	7/24/2020	7/24/2020
.Wyoming	578,759	6.00	8/23/2020				93,231	7/23/2020	7/23/2020

eTable 3. COVID-19 cases

Date/total cases	1/21/2020	1/22/2020	1/23/2020	1/24/2020	1/25/2020	1/26/2020	1/27/2020	1/28/2020	1/29/2020	1/30/2020	1/31/2020	2/1/2020	2/2/2020	2/3/2020	2/4/2020	2/5/2020	2/6/2020	2/7/2020
AK	0	0	0	0	0	0	0	0	0	0	0	0	0	0	0	0	0	0
AL	0	0	0	0	0	0	0	0	0	0	0	0	0	0	0	0	0	0
AR	0	0	0	0	0	0	0	0	0	0	0	0	0	0	0	0	0	0
AZ	0	0	0	0	0	1	1	1	1	1	1	1	1	1	1	1	1	1
CA	0	0	0	0	0	2	2	2	2	2	3	3	3	6	6	6	6	6
CO	0	0	0	0	0	0	0	0	0	0	0	0	0	0	0	0	0	0
CT	0	0	0	0	0	0	0	0	0	0	0	0	0	0	0	0	0	0
DC	0	0	0	0	0	0	0	0	0	0	0	0	0	0	0	0	0	0
DE	0	0	0	0	0	0	0	0	0	0	0	0	0	0	0	0	0	0
FL	0	0	0	0	0	0	0	0	0	0	0	0	0	0	0	0	0	0
GA	0	0	0	0	0	0	0	0	0	0	0	0	0	0	0	0	0	0
HI	0	0	0	0	0	0	0	0	0	0	0	0	0	0	0	0	0	0
IA	0	0	0	0	0	0	0	0	0	0	0	0	0	0	0	0	0	0
ID	0	0	0	0	0	0	0	0	0	0	0	0	0	0	0	0	0	0
IL	0	0	0	1	1	1	1	1	1	1	2	2	2	2	2	2	2	2
IN	0	0	0	0	0	0	0	0	0	0	0	0	0	0	0	0	0	0
KS	0	0	0	0	0	0	0	0	0	0	0	0	0	0	0	0	0	0
KY	0	0	0	0	0	0	0	0	0	0	0	0	0	0	0	0	0	0
LA	0	0	0	0	0	0	0	0	0	0	0	0	0	0	0	0	0	0
MA	0	0	0	0	0	0	0	0	0	0	0	1	1	1	1	1	1	1
MD	0	0	0	0	0	0	0	0	0	0	0	0	0	0	0	0	0	0
ME	0	0	0	0	0	0	0	0	0	0	0	0	0	0	0	0	0	0
MI	0	0	0	0	0	0	0	0	0	0	0	0	0	0	0	0	0	0
MN	0	0	0	0	0	0	0	0	0	0	0	0	0	0	0	0	0	0
MO	0	0	0	0	0	0	0	0	0	0	0	0	0	0	0	0	0	0
MS	0	0	0	0	0	0	0	0	0	0	0	0	0	0	0	0	0	0
MT	0	0	0	0	0	0	0	0	0	0	0	0	0	0	0	0	0	0
NC	0	0	0	0	0	0	0	0	0	0	0	0	0	0	0	0	0	0
ND	0	0	0	0	0	0	0	0	0	0	0	0	0	0	0	0	0	0
NE	0	0	0	0	0	0	0	0	0	0	0	0	0	0	0	0	0	0
NH	0	0	0	0	0	0	0	0	0	0	0	0	0	0	0	0	0	0
NJ	0	0	0	0	0	0	0	0	0	0	0	0	0	0	0	0	0	0
NM	0	0	0	0	0	0	0	0	0	0	0	0	0	0	0	0	0	0
NV	0	0	0	0	0	0	0	0	0	0	0	0	0	0	0	0	0	0
NY	0	0	0	0	0	0	0	0	0	0	0	0	0	0	0	0	0	0
OH	0	0	0	0	0	0	0	0	0	0	0	0	0	0	0	0	0	0
OK	0	0	0	0	0	0	0	0	0	0	0	0	0	0	0	0	0	0
OR	0	0	0	0	0	0	0	0	0	0	0	0	0	0	0	0	0	0
PA	0	0	0	0	0	0	0	0	0	0	0	0	0	0	0	0	0	0
RI	0	0	0	0	0	0	0	0	0	0	0	0	0	0	0	0	0	0
SC	0	0	0	0	0	0	0	0	0	0	0	0	0	0	0	0	0	0
SD	0	0	0	0	0	0	0	0	0	0	0	0	0	0	0	0	0	0
TN	0	0	0	0	0	0	0	0	0	0	0	0	0	0	0	0	0	0
TX	0	0	0	0	0	0	0	0	0	0	0	0	0	0	0	0	0	0
UT	0	0	0	0	0	0	0	0	0	0	0	0	0	0	0	0	0	0
VA	0	0	0	0	0	0	0	0	0	0	0	0	0	0	0	0	0	0
VT	0	0	0	0	0	0	0	0	0	0	0	0	0	0	0	0	0	0
WA	1	1	1	1	1	1	1	1	1	1	1	1	1	1	1	1	1	1
WI	0	0	0	0	0	0	0	0	0	0	0	0	0	0	0	0	0	0
WV	0	0	0	0	0	0	0	0	0	0	0	0	0	0	0	0	0	0
WY	0	0	0	0	0	0	0	0	0	0	0	0	0	0	0	0	0	0
total	1	1	1	2	2	5	5	5	5	5	7	8	8	11	11	11	11	11

2/8/2020	2/9/2020	2/10/2020	2/11/2020	2/12/2020	2/13/2020	2/14/2020	2/15/2020	2/16/2020	2/17/2020	2/18/2020	2/19/2020	2/20/2020	2/21/2020	2/22/2020	2/23/2020	2/24/2020	2/25/2020	2/26/2020	2/27/2020
0	0	0	0	0	0	0	0	0	0	0	0	0	0	0	0	0	0	0	0
0	0	0	0	0	0	0	0	0	0	0	0	0	0	0	0	0	0	0	0
0	0	0	0	0	0	0	0	0	0	0	0	0	0	0	0	0	0	0	0
1	1	1	1	1	1	1	1	1	1	1	1	1	1	1	1	1	1	1	1
6	6	6	7	7	8	8	8	8	8	8	8	8	10	10	10	10	10	10	11
0	0	0	0	0	0	0	0	0	0	0	0	0	0	0	0	0	0	0	0
0	0	0	0	0	0	0	0	0	0	0	0	0	0	0	0	0	0	0	0
0	0	0	0	0	0	0	0	0	0	0	0	0	0	0	0	0	0	0	0
0	0	0	0	0	0	0	0	0	0	0	0	0	0	0	0	0	0	0	0
0	0	0	0	0	0	0	0	0	0	0	0	0	0	0	0	0	0	0	0
0	0	0	0	0	0	0	0	0	0	0	0	0	0	0	0	0	0	0	0
0	0	0	0	0	0	0	0	0	0	0	0	0	0	0	0	0	0	0	0
0	0	0	0	0	0	0	0	0	0	0	0	0	0	0	0	0	0	0	0
0	0	0	0	0	0	0	0	0	0	0	0	0	0	0	0	0	0	0	0
2	2	2	2	2	2	2	2	2	2	2	2	2	2	2	2	2	2	2	2
0	0	0	0	0	0	0	0	0	0	0	0	0	0	0	0	0	0	0	0
0	0	0	0	0	0	0	0	0	0	0	0	0	0	0	0	0	0	0	0
0	0	0	0	0	0	0	0	0	0	0	0	0	0	0	0	0	0	0	0
0	0	0	0	0	0	0	0	0	0	0	0	0	0	0	0	0	0	0	0
1	1	1	1	1	1	1	1	1	1	1	1	1	1	1	1	1	1	1	1
0	0	0	0	0	0	0	0	0	0	0	0	0	0	0	0	0	0	0	0
0	0	0	0	0	0	0	0	0	0	0	0	0	0	0	0	0	0	0	0
0	0	0	0	0	0	0	0	0	0	0	0	0	0	0	0	0	0	0	0
0	0	0	0	0	0	0	0	0	0	0	0	0	0	0	0	0	0	0	0
0	0	0	0	0	0	0	0	0	0	0	0	0	0	0	0	0	0	0	0
0	0	0	0	0	0	0	0	0	0	0	0	0	0	0	0	0	0	0	0
0	0	0	0	0	0	0	0	0	0	0	0	0	0	0	0	0	0	0	0
0	0	0	0	0	0	0	0	0	0	0	0	0	0	0	0	0	0	0	0
0	0	0	0	0	0	0	0	0	0	0	0	0	0	0	0	0	0	0	0
0	0	0	0	0	0	0	0	0	0	0	0	0	0	0	0	0	0	0	0
0	0	0	0	0	0	0	0	0	0	0	0	0	0	0	0	0	0	0	0
0	0	0	0	0	0	0	0	0	0	0	0	0	0	0	0	0	0	0	0
0	0	0	0	0	0	0	0	0	0	0	0	0	0	0	0	0	0	0	0
0	0	0	0	0	0	0	0	0	0	0	0	0	0	0	0	0	0	0	0
0	0	0	0	0	0	0	0	0	0	0	0	0	0	0	0	0	0	0	0
0	0	0	0	0	0	0	0	0	0	0	0	0	0	0	0	0	0	0	0
0	0	0	0	0	0	0	0	0	0	0	0	0	0	0	0	0	0	0	0
0	0	0	0	0	0	0	0	0	0	0	0	0	0	0	0	0	0	0	0
0	0	0	0	0	0	0	0	0	0	0	0	0	0	0	0	0	0	0	0
0	0	0	0	0	0	0	0	0	0	0	0	0	0	0	0	0	0	0	0
0	0	0	0	0	0	0	0	0	0	0	0	0	0	0	0	0	0	0	0
0	0	0	0	0	0	0	0	0	0	0	0	0	0	0	0	0	0	0	0
0	0	0	0	0	0	0	0	0	0	0	0	0	0	0	0	0	0	0	0
0	0	0	0	0	0	0	0	0	0	0	0	0	0	0	0	0	0	0	0
0	0	0	0	0	0	0	0	0	0	0	0	0	0	0	0	0	0	0	0
0	0	0	0	0	0	0	0	0	0	0	0	0	0	0	0	0	0	0	0
0	0	0	0	0	0	0	0	0	0	0	0	0	0	0	0	0	0	0	0
1	1	1	1	1	1	1	1	1	1	1	1	1	1	1	1	1	1	1	1
0	0	0	0	0	0	0	0	0	0	0	0	0	0	0	0	0	0	0	0
0	0	0	0	0	0	0	0	0	0	0	0	0	0	0	0	0	0	0	0
0	0	0	0	0	0	0	0	0	0	0	0	0	0	0	0	0	0	0	0
11	11	11	12	12	13	13	13	13	13	13	13	13	15	15	15	15	15	15	16

2/28/2020	2/29/2020	3/1/2020	3/2/2020	3/3/2020	3/4/2020	3/5/2020	3/6/2020	3/7/2020	3/8/2020	3/9/2020	3/10/2020	3/11/2020	3/12/2020	3/13/2020	3/14/2020	3/15/2020	3/16/2020	3/17/2020	3/18/2020	3/19/2020
0	0	0	0	0	0	0	0	0	0	0	0	0	0	1	1	1	1	3	6	9
0	0	0	0	0	0	0	0	0	0	0	0	0	0	1	1	22	29	39	51	78
0	0	0	0	0	0	0	0	0	0	0	0	1	6	9	12	16	17	24	37	64
1	1	1	1	2	2	3	3	5	5	6	8	9	9	10	12	13	18	18	27	44
11	12	12	21	29	36	45	56	56	110	135	152	175	224	264	311	369	447	596	652	982
0	0	0	0	0	0	1	2	8	8	12	17	34	49	49	49	144	160	183	216	277
0	0	0	0	0	0	0	0	0	1	1	2	3	7	7	7	26	41	68	96	159
0	0	0	0	0	0	0	0	1	1	2	2	2	2	3	3	17	22	31	39	71
0	0	0	0	0	0	0	0	0	0	0	0	1	4	5	8	8	8	16	26	30
0	0	0	1	2	2	3	3	6	6	19	19	26	31	31	55	136	142	195	299	393
0	0	0	0	2	2	2	3	6	7	18	23	31	45	45	45	119	143	178	262	397
0	0	0	0	0	0	0	0	1	1	1	1	1	1	2	2	7	10	10	10	20
0	0	0	0	0	0	0	0	0	3	8	13	14	16	16	16	22	23	29	38	44
0	0	0	0	0	0	0	0	0	0	0	0	0	0	0	1	5	5	5	10	23
2	2	3	4	4	4	5	6	6	7	11	19	27	32	32	32	93	105	160	288	422
0	0	0	0	0	0	0	1	1	2	3	5	9	12	12	12	19	24	30	39	56
0	0	0	0	0	0	0	0	0	1	1	1	1	4	6	7	8	11	16	21	35
0	0	0	0	0	0	0	0	1	1	1	4	4	11	11	15	20	22	26	35	43
0	0	0	0	0	0	0	0	0	0	1	1	8	14	33	33	101	137	190	261	392
1	1	1	1	2	2	2	7	9	28	28	91	95	108	109	109	164	197	218	256	328
0	0	0	0	0	0	3	3	3	3	9	9	12	12	12	12	37	57	85	107	149
0	0	0	0	0	0	0	0	0	0	0	0	0	1	1	5	14	19	32	44	52
0	0	0	0	0	0	0	0	0	0	0	2	2	3	13	25	53	54	65	80	334
0	0	0	0	0	0	0	1	1	2	2	4	5	10	14	14	54	60	77	89	115
0	0	0	0	0	0	0	0	1	1	1	1	1	1	2	2	5	8	13	24	28
0	0	0	0	0	0	0	0	0	0	0	0	0	1	1	6	10	12	34	50	80
0	0	0	0	0	0	0	0	0	0	0	0	1	1	1	1	7	7	11	12	15
0	0	0	0	1	1	1	2	2	2	7	7	8	15	15	17	33	40	62	97	137
0	0	0	0	0	0	0	0	0	0	0	0	0	1	1	1	1	1	5	7	19
0	0	0	0	0	0	0	1	2	2	4	4	5	10	10	10	18	18	21	27	27
0	0	0	1	2	2	2	2	2	4	4	5	5	6	6	6	13	17	26	39	44
0	0	0	0	0	0	2	4	6	6	8	15	23	29	30	44	98	178	267	427	742
0	0	0	0	0	0	0	0	0	0	0	0	3	5	5	7	17	21	23	28	35
0	0	0	0	0	0	1	1	2	3	4	5	7	11	11	11	16	45	55	63	95
0	0	0	1	1	2	14	23	30	40	142	173	217	325	362	461	669	831	2601	4597	6834
0	0	0	0	0	0	0	0	0	0	0	3	3	5	5	5	37	50	67	88	119
0	0	0	0	0	0	0	0	1	1	1	2	2	3	5	5	9	10	19	31	49
0	1	1	3	3	3	3	3	7	14	14	15	19	24	24	24	39	47	65	75	88
0	0	0	0	0	0	0	2	4	6	10	12	16	23	29	47	63	76	96	133	185
0	0	1	2	2	2	2	2	3	3	3	5	5	5	5	9	21	23	23	33	44
0	0	0	0	0	0	0	0	2	6	7	9	10	12	12	12	28	33	47	60	81
0	0	0	0	0	0	0	0	0	0	0	5	8	8	9	9	9	10	11	11	14
0	0	0	0	0	0	1	1	1	1	1	7	9	18	26	26	39	52	73	98	154
0	0	0	0	0	0	3	5	5	6	13	15	23	24	36	43	51	57	64	83	143
0	0	0	0	0	0	0	0	0	1	1	2	3	4	6	6	21	29	29	40	68
0	0	0	0	0	0	0	0	0	2	5	8	15	29	29	29	51	67	68	94	114
0	0	0	0	0	0	0	0	0	1	1	1	1	2	2	3	4	8	10	12	16
1	7	11	18	27	39	70	79	102	136	162	267	366	457	568	642	708	769	930	1,187	1,376
0	0	0	0	1	1	1	1	1	1	1	3	5	8	9	19	27	53	88	106	155
0	0	0	0	0	0	0	0	0	0	0	0	0	0	0	0	0	0	1	2	5
0	0	0	0	0	0	0	0	0	0	0	0	0	1	1	2	3	3	11	16	18
16	24	30	53	78	98	164	211	275	422	647	937	1215	1629	1896	2234	3465	4217	7014	10429	15202

3/20/2020	3/21/2020	3/22/2020	3/23/2020	3/24/2020	3/25/2020	3/26/2020	3/27/2020	3/28/2020	3/29/2020	3/30/2020	3/31/2020	4/1/2020	4/2/2020	4/3/2020	4/4/2020	4/5/2020	4/6/2020	4/7/2020	4/8/2020	4/9/2020
12	14	22	36	42	59	69	85	102	114	119	133	143	147	157	171	185	191	213	226	235
106	131	157	196	242	386	531	639	720	830	947	999	1,106	1,270	1,535	1,633	1,841	2,006	2,197	2,499	2,838
100	118	164	197	230	306	349	386	409	443	504	560	614	679	738	743	853	915	993	1,071	1,127
63	104	152	253	326	401	508	665	773	919	1,157	1,289	1,413	1,598	1,769	2,019	2,269	2,456	2,575	2,726	3,018
1,304	1,534	1,709	1,931	2,511	2,982	3,777	4,730	5,259	5,739	6,909	8,131	8,155	9,191	12,024	12,026	13,438	14,336	15,865	16,957	18,309
363	475	591	720	921	1,086	1,430	1,734	2,061	2,307	2,627	2,966	3,342	3,728	4,173	4,565	4,950	5,172	5,429	5,655	6,202
194	223	327	415	618	875	1,012	1,291	1,524	1,993	2,571	3,128	3,557	3,824	4,915	5,276	5,675	6,906	7,781	8,781	9,784
77	98	116	137	183	231	267	304	342	401	495	495	586	653	757	902	998	1,097	1,211	1,440	1,523
39	45	56	87	104	119	143	165	214	232	264	319	368	393	450	593	673	783	928	1,116	1,209
510	706	937	1,141	1,373	1,861	2,352	3,054	3,877	4,768	5,489	6,490	7,495	8,694	9,925	11,173	11,961	13,214	14,302	15,234	16,323
485	555	757	1,015	1,119	1,441	1,714	2,198	2,446	2,703	3,612	4,585	4,748	5,486	5,967	6,383	6,752	8,634	9,713	10,446	11,318
37	48	48	66	70	76	86	98	126	141	163	185	205	225	270	310	324	332	362	377	386
45	68	90	105	124	145	179	235	298	336	424	497	549	614	699	786	868	946	1,048	1,145	1,270
31	42	47	51	71	123	189	230	261	310	415	525	669	891	1,013	1,077	1,101	1,170	1,210	1,232	1,353
585	753	1,049	1,285	1,535	1,865	2,538	3,026	3,491	4,596	5,057	5,994	6,980	7,695	8,904	10,357	11,256	12,262	13,549	15,078	16,422
79	126	201	259	365	477	645	981	1,232	1,514	1,786	2,159	2,565	3,039	3,457	3,953	4,411	4,944	5,507	5,943	6,351
44	55	64	82	98	98	168	202	261	319	368	428	482	552	620	698	813	845	900	1,076	1,106
48	87	103	124	163	198	248	302	394	439	480	591	680	770	831	917	955	1,008	1,149	1,346	1,452
537	763	1,035	1,172	1,388	1,946	2,305	2,746	3,315	3,540	4,025	5,237	6,424	9,150	10,297	12,496	13,010	14,867	16,284	17,030	18,283
413	525	646	777	1,159	1,838	2,417	3,240	4,257	4,955	5,752	6,620	7,738	8,966	10,402	11,736	12,500	13,837	15,202	16,567	18,941
149	190	288	288	349	580	774	774	992	1,239	1,660	1,660	1,985	2,331	2,758	3,125	4,045	4,371	5,529	6,185	6,968
56	70	93	109	118	147	155	168	211	253	303	303	344	376	432	456	470	499	519	537	560
549	787	1,035	1,328	1,791	2,294	2,856	3,657	4,650	5,486	6,498	7,615	9,334	10,791	12,670	14,225	15,718	17,221	18,970	20,346	21,504
115	137	236	262	287	346	399	399	441	576	576	689	742	789	789	865	986	1,069	1,154	1,242	1,336
73	90	106	183	255	356	502	670	838	903	1,031	1,327	1,581	1,834	2,113	2,291	2,367	2,722	3,037	3,327	3,539
80	140	249	320	377	485	579	579	663	847	937	1,073	1,177	1,358	1,358	1,455	1,738	1,915	2,003	2,260	2,469
21	27	34	37	48	65	90	121	147	173	184	203	228	244	262	281	300	320	332	352	365
164	227	255	398	504	636	764	764	935	1,307	1,498	1,584	1,857	2,093	2,093	2,402	2,870	2,870	3,221	3,651	3,651
26	28	30	32	37	45	58	68	94	98	109	126	147	159	173	186	207	225	237	251	269
38	42	50	52	53	64	73	89	108	120	145	177	214	255	285	323	367	412	478	523	577
55	65	78	101	108	137	158	187	214	258	314	367	415	479	540	621	669	715	747	788	819
890	1,327	1,914	2,844	3,675	4,402	6,876	8,825	11,124	13,386	16,636	18,696	22,255	25,590	29,895	34,124	37,505	41,090	44,416	47,437	51,027
43	57	65	83	100	112	136	191	208	237	281	315	363	403	495	543	624	686	794	865	989
109	154	190	245	278	321	535	621	738	920	1,044	1,113	1,279	1,458	1,514	1,742	1,836	1,953	2,087	2,318	2,456
7845	10356	15168	21689	26358	32966	38977	45934	53308	59219	67131	74427	84735	90279	101993	111248	119435	141100	138773	149316	157073
169	247	351	442	564	704	867	1,137	1,406	1,653	1,933	2,199	2,547	2,902	3,312	3,739	4,043	4,450	4,782	5,148	5,512
49	53	67	81	106	223	248	322	377	481	481	565	719	879	988	1,159	1,250	1,327	1,472	1,524	1,684
114	137	161	191	209	266	316	414	479	548	606	690	736	826	899	999	1,068	1,132	1,181	1,239	1,321
269	371	479	644	851	1,128	1,688	2,219	2,752	3,394	4,087	4,843	5,805	7,016	8,421	10,017	11,510	12,980	14,559	16,239	18,228
54	66	97	106	124	132	188	203	239	294	479	520	566	681	711	806	922	1,170	1,414	1,450	1,939
125	173	195	298	342	424	456	539	660	774	925	1,083	1,293	1,554	1,700	1,917	2,049	2,232	2,417	2,552	2,792
14	14	21	28	30	41	46	58	68	90	101	108	129	165	187	212	240	288	320	393	447
228	371	505	615	667	784	957	1,203	1,373	1,537	1,834	2,239	2,240	2,845	2,880	3,103	3,633	3,803	4,139	4,362	4,635
401	507	507	507	715	974	1,396	1,731	2,052	2,552	2,877	3,266	3,997	4,669	5,330	6,110	6,812	7,276	8,262	9,353	10,230
102	125	169	280	298	346	402	466	588	789	806	934	1,012	1,165	1,246	1,428	1,605	1,716	1,804	1,916	2,061
114	152	254	290	391	460	604	604	739	1,020	1,020	1,484	1,706	2,012	2,012	2,407	2,878	3,333	3,645	4,042	4,509
29	49	52	75	95	123	156	184	211	235	256	293	321	338	389	461	512	543	575	605	628
1,524	1,793	1,996	2,221	2,469	2,580	3,207	3,723	4,310	4,506	4,896	4,896	5,319	5,683	6,966	7,591	6,973	7,318	8,682	9,097	9,608
206	281	381	416	457	585	707	842	989	989	1,221	1,351	1,550	1,730	1,916	2,112	2,267	2,440	2,578	2,756	2,885
8	12	16	20	39	51	76	96	113	124	145	162	191	217	237	282	324	345	412	412	523
22	24	24	26	30	44	55	73	84	87	95	120	137	150	166	187	200	212	221	230	239
18713	24542	33337	44260	54367	68334	85228	103172	122473	140694	163273	185759	212743	238836	273633	304231	330256	373654	395178	426661	458293

4/10/2020	4/11/2020	4/12/2020	4/13/2020	4/14/2020	4/15/2020	4/16/2020	4/17/2020	4/18/2020	4/19/2020	4/20/2020	4/21/2020	4/22/2020	4/23/2020	4/24/2020	4/25/2020	4/26/2020	4/27/2020	4/28/2020	4/29/2020
246	257	272	277	285	293	300	309	314	319	321	329	335	337	339	339	341	345	351	355
3,008	3,262	3,583	3,803	3,953	4,241	4,404	4,572	4,723	4,946	5,424	5,668	5,831	6,071	6,026	6,213	6,627	6,539	6,947	7,105
1,202	1,234	1,307	1,410	1,498	1,583	1,635	1,702	1,777	1,781	1,964	2,262	2,281	2,465	2,810	2,909	2,941	3,055	3,121	3,192
3,112	3,393	3,539	3,702	3,806	3,962	4,234	4,507	4,719	4,929	5,064	5,251	5,459	5,769	6,045	6,280	6,526	6,716	6,948	7,202
19,472	20,615	21,794	22,348	23,338	24,424	26,182	27,528	28,963	28,963	30,978	33,261	35,396	37,369	39,254	41,137	42,164	43,464	45,031	46,500
6,365	6,893	7,303	7,691	7,941	8,280	8,582	9,047	9,433	9,634	10,019	10,368	10,825	11,182	12,184	12,868	13,350	13,798	14,238	14,675
10,131	11,510	12,035	13,381	13,989	14,755	15,884	16,809	17,550	17,962	19,815	20,360	22,469	23,100	23,921	24,582	25,269	25,997	26,312	26,767
1,660	1,778	1,875	1,955	2,058	2,197	2,350	2,476	2,666	2,793	2,927	3,098	3,206	3,361	3,528	3,699	3,841	3,892	3,994	4,106
1,326	1,479	1,625	1,761	1,926	2,014	2,075	2,323	2,538	2,745	2,745	3,200	3,308	3,442	3,442	3,576	4,162	4,575	4,655	4,734
17,448	18,445	19,337	20,394	20,984	21,865	22,674	24,066	24,797	25,598	26,329	27,127	27,791	28,843	29,707	29,996	30,680	31,290	31,986	32,318
11,318	12,261	13,012	13,621	14,766	15,409	16,451	16,658	17,014	18,781	19,630	20,607	20,769	21,681	22,183	22,225	23,410	23,801	24,609	25,324
409	452	465	504	517	517	541	553	574	580	584	547	537	540	601	604	550	607	553	557
1,388	1,510	1,587	1,710	1,899	1,995	2,141	2,332	2,513	2,902	3,159	3,641	3,748	3,924	4,445	5,092	5,475	5,868	6,376	6,843
1,396	1,407	1,458	1,486	1,486	1,587	1,609	1,655	1,668	1,672	1,736	1,766	1,802	1,836	1,870	1,887	1,897	1,917	1,952	1,984
17,887	19,180	20,852	22,025	23,247	24,593	25,733	27,575	29,160	30,357	31,508	33,059	35,108	36,934	39,658	41,777	43,903	45,883	48,102	50,355
6,907	7,435	7,928	8,236	8,527	8,955	9,542	10,154	10,641	11,210	11,686	12,097	12,438	13,039	13,680	14,395	15,012	15,961	16,588	17,182
1,241	1,279	1,377	1,376	1,477	1,494	1,588	1,760	1,811	1,849	1,986	2,025	2,211	2,482	2,777	3,056	3,174	3,328	3,491	3,738
1,693	1,840	1,963	2,048	2,210	2,291	2,429	2,522	2,707	2,960	3,050	3,192	3,373	3,481	3,779	3,905	4,074	4,146	4,375	4,539
19,253	20,014	20,595	21,016	21,518	21,951	22,532	23,118	23,580	23,928	24,523	24,854	25,317	25,798	26,140	26,512	26,832	27,111	27,329	27,703
20,845	23,918	25,475	26,867	28,163	29,918	32,181	34,402	36,372	38,077	39,643	41,199	42,944	46,023	50,969	53,348	54,938	56,462	58,302	60,265
6,968	7,694	8,936	9,472	10,032	10,784	11,572	11,572	12,308	13,684	14,193	14,775	15,737	16,616	16,616	17,766	19,487	20,113	20,849	21,742
583	616	633	698	734	770	796	827	847	867	875	888	907	937	965	990	1,023	1,040	1,056	1,095
22,562	23,853	24,638	25,635	27,001	28,059	29,263	29,952	30,717	31,424	32,000	32,967	33,966	35,291	36,627	37,184	37,778	38,210	39,262	40,399
1,336	1,514	1,621	1,695	1,809	1,862	2,013	2,174	2,213	2,470	2,567	2,721	2,721	3,185	3,185	3,446	3,602	4,181	4,645	5,136
3,799	4,024	4,160	4,388	4,686	4,895	5,111	5,283	5,517	5,667	5,807	5,941	6,137	6,321	6,625	6,826	6,997	7,171	7,303	7,425
2,469	2,642	2,942	3,087	3,360	3,624	3,793	3,793	3,974	4,512	4,716	4,716	5,153	5,434	5,434	5,718	6,094	6,342	6,569	6,569
365	377	394	398	404	415	422	422	426	433	437	439	442	444	444	445	449	451	451	451
3,908	4,312	4,520	4,816	5,024	5,123	5,465	5,859	6,140	6,493	6,764	6,951	7,220	7,608	8,052	8,623	8,830	9,142	9,568	9,948
278	293	308	331	341	365	393	439	528	585	627	644	679	709	748	803	867	942	991	1,033
648	704	814	871	901	952	1,066	1,138	1,287	1,474	1,648	1,722	1,813	2,124	2,421	2,732	3,028	3,358	3,374	3,784
885	929	985	1,020	1,091	1,139	1,211	1,287	1,342	1,392	1,447	1,491	1,588	1,670	1,720	1,787	1,864	1,938	2,010	2,054
54,588	58,151	61,850	64,584	68,824	71,030	75,317	78,467	81,436	85,301	88,806	92,387	95,865	99,989	102,196	105,523	109,038	111,188	113,856	116,264
1,091	1,174	1,245	1,345	1,407	1,484	1,597	1,711	1,798	1,845	1,971	2,072	2,210	2,379	2,521	2,660	2,726	2,823	2,974	3,213
2,584	2,700	2,836	2,971	3,088	3,211	3,321	3,524	3,626	3,728	3,830	3,937	4,081	4,208	4,398	4,539	4,602	4,690	4,812	4,898
170884	180458	188694	195081	201834	211550	216703	224438	233570	238138	245580	250800	253219	263744	267256	277606	282991	287607	290481	294715
5,878	6,250	6,518	6,975	7,280	7,791	8,414	9,107	10,222	11,602	12,919	13,725	14,117	14,694	15,169	15,587	15,963	16,325	16,769	17,303
1,794	1,868	1,970	2,069	2,209	2,263	2,359	2,465	2,570	2,599	2,680	2,807	2,894	3,017	3,121	3,193	3,253	3,280	3,410	3,473
1,371	1,447	1,527	1,584	1,633	1,663	1,736	1,785	1,844	1,910	1,956	2,002	2,059	2,127	2,177	2,253	2,311	2,354	2,385	2,446
19,980	21,656	22,833	24,199	25,345	26,490	27,735	29,442	31,070	32,284	33,232	34,528	35,684	37,053	38,652	40,049	41,165	42,050	43,264	44,366
2,015	2,349	2,665	2,976	3,369	3,529	3,969	4,177	4,491	4,706	5,090	5,716	6,012	6,256	6,699	7,129	7,439	7,708	7,926	8,247
3,065	3,207	3,319	3,439	3,553	3,656	3,931	4,086	4,246	4,377	4,439	4,608	4,761	4,917	5,070	5,253	5,490	5,613	5,735	5,881
536	626	730	868	1,168	1,168	1,311	1,411	1,542	1,635	1,685	1,754	1,858	1,956	2,040	2,147	2,212	2,245	2,313	2,373
4,863	4,863	5,308	5,610	5,823	5,836	6,262	6,330	6,763	6,796	7,239	7,394	7,572	8,281	8,742	8,882	9,698	9,952	10,087	10,132
11,671	12,561	13,484	13,906	14,624	15,492	16,455	17,371	18,260	18,923	19,458	20,196	21,069	21,944	22,806	23,773	24,631	25,297	26,171	27,054
2,102	2,206	2,338	2,392	2,453	2,637	2,760	2,805	2,946	3,151	3,259	3,369	3,540	3,722	3,782	3,948	4,214	4,307	4,423	4,605
4,509	5,077	5,747	6,171	6,500	6,889	7,491	8,053	8,053	8,990	9,630	9,630	10,998	11,594	12,366	12,366	13,535	14,339	14,961	15,846
679	711	727	748	750	759	768	779	803	812	816	818	823	825	827	843	851	855	862	862
9,887	10,224	10,411	10,538	10,694	10,783	11,152	11,445	11,802	11,790	12,085	12,282	12,494	12,753	12,977	13,319	13,521	13,686	13,842	14,070
3,068	3,221	3,341	3,428	3,555	3,721	3,875	4,045	4,199	4,346	4,499	4,620	4,845	5,052	5,356	5,687	5,911	6,081	6,289	6,520
574	591	611	626	694	718	739	775	825	863	902	914	963	981	1,010	1,025	1,053	1,077	1,095	1,109
253	261	270	373	494	393	401	412	423	426	428	441	447	453	473	491	502	520	536	544
491500	524721	553757	577905	604268	631375	660468	689442	719308	745209	774676	801166	827022	863961	893833	926993	956291	979640	1003529	1029001

4/30/2020	5/1/2020	5/2/2020	5/3/2020	5/4/2020	5/5/2020	5/6/2020	5/7/2020	5/8/2020	5/9/2020	5/10/2020	5/11/2020	5/12/2020	5/13/2020	5/14/2020	5/15/2020	5/16/2020	5/17/2020	5/18/2020	5/19/2020	5/20/2020
355	364	365	368	370	371	372	374	377	378	379	381	383	383	387	388	392	396	399	399	402
7,306	7,294	7,611	7,888	8,312	8,679	8,691	9,243	9,639	9,923	10,145	10,413	10,665	10,936	11,338	11,642	11,981	11,771	12,587	12,376	13,300
3,255	3,310	3,372	3,431	3,469	3,496	3,611	3,665	3,747	3,984	3,984	4,043	4,164	4,236	4,348	4,463	4,578	4,759	4,813	4,923	5,003
7,648	7,962	8,364	8,640	8,919	9,305	9,707	9,945	10,526	10,960	11,119	11,380	11,736	12,176	12,674	13,169	13,631	13,937	14,170	14,566	14,897
48,917	50,442	52,197	53,616	54,937	56,212	58,815	60,614	62,512	64,561	66,680	67,939	69,382	71,141	73,164	74,936	76,793	78,839	80,430	81,795	84,057
15,182	15,668	16,120	16,534	16,878	17,317	17,738	18,318	18,793	19,316	19,632	19,735	20,091	20,401	20,762	21,131	21,511	21,797	22,095	22,399	22,752
27,700	28,764	29,287	29,312	29,973	30,621	30,995	31,784	32,411	32,984	33,554	33,765	34,333	34,855	35,464	36,085	36,703	37,419	38,116	38,430	39,017
4,323	4,658	4,797	5,016	5,170	5,322	5,461	5,654	5,899	6,102	6,272	6,389	6,485	6,584	6,736	6,871	7,042	7,123	7,270	7,434	7,551
4,734	4,918	5,038	5,208	5,288	5,371	5,778	6,111	6,111	6,447	6,565	6,741	6,952	7,223	7,373	7,373	7,547	7,869	8,037	8,194	8,386
33,690	34,728	35,463	35,969	36,897	37,439	38,002	38,828	39,199	40,001	39,888	40,982	41,923	42,402	43,210	42,940	44,811	45,588	46,442	46,944	47,471
26,095	27,733	28,338	28,621	29,196	29,602	30,750	31,383	32,609	32,590	32,980	33,995	34,731	35,335	35,858	36,680	37,147	37,642	38,081	38,753	39,702
562	619	620	557	558	570	626	576	576	579	580	582	583	587	586	587	588	589	589	591	593
7,145	7,884	8,641	9,169	9,703	10,111	10,404	11,059	11,457	11,671	11,959	12,373	12,912	13,289	13,675	14,049	14,328	14,651	15,082	15,349	15,595
2,015	2,035	2,061	2,061	2,106	2,127	2,158	2,178	2,205	2,230	2,230	2,260	2,293	2,324	2,351	2,389	2,419	2,419	2,455	2,476	2,506
52,918	56,055	58,505	61,449	63,840	65,962	68,232	70,873	73,760	76,085	77,741	79,007	83,021	84,698	87,937	90,369	92,457	94,191	96,485	98,030	100,418
17,835	18,630	19,295	19,933	20,507	21,033	21,870	22,503	23,146	23,732	24,126	24,627	25,127	25,473	26,053	26,655	27,280	27,778	28,255	28,705	29,274
4,238	4,449	4,746	5,030	5,245	5,458	5,734	6,144	6,501	6,751	6,984	7,116	7,116	7,468	7,468	7,886	7,886	7,886	8,340	8,340	8,539
4,708	4,879	4,879	5,130	5,245	5,822	5,934	6,129	6,288	6,440	6,440	6,677	6,853	7,080	7,225	7,444	7,688	7,688	7,935	8,069	8,167
28,044	28,711	29,140	29,383	29,746	30,069	30,399	30,725	30,928	31,490	31,673	31,881	32,116	32,728	33,555	33,903	34,183	34,498	34,832	35,161	35,439
62,205	64,311	66,263	68,087	69,087	70,271	72,025	73,721	75,333	76,743	77,793	78,462	79,332	80,497	82,182	83,421	84,933	86,010	87,052	87,925	88,970
23,472	23,472	24,473	26,408	27,117	28,163	28,163	30,485	31,534	32,587	33,373	34,061	34,812	35,903	36,986	37,968	38,804	38,804	41,546	42,323	43,531
1,123	1,123	1,152	1,205	1,226	1,254	1,254	1,374	1,374	1,434	1,457	1,462	1,515	1,515	1,603	1,648	1,687	1,713	1,741	1,819	1,877
41,379	42,356	43,207	43,754	43,950	44,397	45,054	45,646	46,326	46,756	47,138	47,552	48,021	48,391	49,582	50,079	50,504	51,142	51,915	52,350	53,009
5,136	5,730	6,228	7,238	7,234	7,851	8,579	10,088	10,088	11,271	11,271	11,799	12,917	12,917	14,240	14,240	15,668	16,372	17,029	17,670	17,670
7,562	7,835	8,154	8,386	8,754	8,916	9,102	9,341	9,489	9,666	9,844	9,918	10,006	10,142	10,316	10,456	10,675	10,789	10,945	11,080	11,232
7,212	7,212	7,441	7,877	8,207	8,422	8,424	9,090	9,378	9,501	9,674	9,908	10,093	10,483	10,801	11,123	11,296	11,432	11,704	11,967	12,222
453	453	455	457	457	456	456	459	458	458	458	459	459	462	466	466	468	470	470	478	478
10,509	10,923	11,509	11,664	11,848	12,256	12,758	13,397	13,868	14,360	14,764	15,045	15,346	15,816	16,507	17,129	17,982	18,512	19,023	19,700	20,122
1,067	1,107	1,153	1,191	1,225	1,266	1,323	1,371	1,425	1,464	1,491	1,518	1,571	1,647	1,712	1,761	1,848	1,900	1,931	1,994	2,095
4,281	4,838	5,326	5,659	6,083	6,438	6,771	7,190	7,831	8,234	8,315	8,572	8,692	9,075	9,416	9,772	10,220	10,348	10,625	10,846	11,122
2,146	2,310	2,429	2,518	2,588	2,636	2,740	2,843	2,947	3,011	3,071	3,160	3,239	3,299	3,382	3,464	3,556	3,596	3,652	3,721	3,868
118,652	121,190	123,717	126,744	128,269	130,593	131,890	133,635	135,454	137,085	138,532	139,945	140,743	141,560	142,704	143,905	145,089	146,334	148,039	149,013	150,399
3,411	3,513	3,732	3,850	4,031	4,138	4,291	4,493	4,673	4,778	4,863	5,069	5,212	5,364	5,503	5,662	5,847	5,938	6,096	6,192	6,317
5,007	5,227	5,311	5,426	5,500	5,603	5,663	5,766	5,884	6,028	6,109	6,163	6,322	6,405	6,499	6,629	6,662	6,872	6,921	7,046	7,182
299106	303129	307506	312618	313836	316041	318915	321962	324710	328599	334640	336017	336681	338617	340799	343304	351872	347936	349214	356179	357431
18,027	18,743	19,335	19,914	20,474	20,969	21,576	22,131	23,016	23,697	24,081	24,777	25,250	25,721	26,357	26,954	27,474	27,923	28,272	28,952	29,436
3,618	3,748	3,851	3,972	4,044	4,054	4,201	4,210	4,210	4,335	4,589	4,439	4,439	4,741	4,861	4,971	4,861	5,310	5,247	5,407	5,407
2,510	2,579	2,635	2,680	2,759	2,839	2,916	2,989	3,068	3,160	3,228	3,286	3,358	3,416	3,479	3,541	3,612	3,623	3,687	3,726	3,801
45,763	46,971	48,305	49,267	50,092	50,957	51,845	52,915	54,238	55,316	56,611	57,154	57,991	58,698	59,636	60,622	61,611	62,234	63,666	63,936	64,412
8,621	8,962	9,289	9,477	9,652	9,933	10,205	10,530	10,779	10,989	11,274	11,450	11,614	11,835	12,016	12,219	12,434	12,674	12,795	12,951	13,356
6,095	6,258	6,489	6,626	6,757	6,841	6,936	7,142	7,367	7,531	7,653	7,792	7,927	8,030	8,189	8,407	8,661	8,816	8,942	9,056	9,175
2,449	2,525	2,588	2,631	2,668	2,721	2,779	2,905	3,144	3,393	3,517	3,614	3,663	3,732	3,792	3,887	3,959	3,987	4,027	4,085	4,177
10,735	11,891	12,661	13,243	13,571	13,690	13,938	14,166	14,512	14,843	15,060	15,622	16,190	16,451	16,784	17,052	17,374	17,495	18,121	18,491	18,646
28,087	29,229	30,552	31,548	32,332	33,369	34,422	35,390	36,609	37,860	38,869	39,869	41,048	42,403	43,851	45,198	46,999	47,784	48,693	49,912	51,323
4,753	4,828	4,981	5,261	5,406	5,530	5,595	5,794	6,041	6,234	6,317	6,395	6,548	6,675	6,837	7,012	7,166	7,305	7,459	7,595	7,808
16,901	16,901	17,731	19,492	19,492	20,256	20,256	22,342	22,342	23,196	25,070	25,800	26,746	27,813	28,672	29,683	30,388	31,140	32,145	32,908	34,137
866	879	886	897	902	906	908	916	918	921	927	926	927	929	933	933	932	940	940	944	945
14,327	14,637	15,003	15,185	15,462	15,594	15,905	16,231	16,388	16,674	16,891	17,122	17,330	17,512	17,773	17,951	18,287	18,433	18,611	18,811	18,971
6,854	7,314	7,660	7,964	8,236	8,566	8,901	9,215	9,590	9,939	10,219	10,418	10,611	10,902	11,275	11,685	12,187	12,543	12,687	12,885	13,431
1,125	1,151	1,184	1,195	1,224	1,242	1,276	1,297	1,323	1,347	1,362	1,369	1,378	1,404	1,434	1,447	1,470	1,490	1,502	1,514	1,567
559	566	579	586	596	604	631	635	644	653	662	669	675	688	701	716	741	754	766	776	787
1060681	1091014	1120624	1150335	1169438	1191659	1214975	1245775	1271645	1298287	1322054	1340098	1361522	1382362	1409452	1432265	1464232	1477459	1501876	1525186	1547973

5/21/2020	5/22/2020	5/23/2020	5/24/2020	5/25/2020	5/26/2020	5/27/2020	5/28/2020	5/29/2020	5/30/2020	5/31/2020	6/1/2020	6/2/2020	6/3/2020	6/4/2020	6/5/2020	6/6/2020	6/7/2020	6/8/2020	6/9/2020	6/10/2020
402	404	408	408	409	411	412	425	430	434	460	467	487	505	513	524	536	544	563	573	593
13,647	13,941	14,388	14,741	15,257	15,805	16,262	16,729	17,322	17,649	18,245	18,438	18,771	18,851	19,082	19,396	20,054	20,500	21,430	21,422	22,000
5,458	5,612	5,775	5,922	6,029	6,180	6,277	6,538	6,777	7,013	7,253	7,443	7,818	8,067	8,425	8,651	9,101	9,426	9,740	10,080	10,368
15,315	15,608	16,039	16,339	16,561	16,783	17,262	17,763	18,465	19,255	19,936	20,123	21,250	22,223	22,753	24,332	25,451	26,889	27,678	28,296	29,852
86,197	88,444	90,631	92,710	94,558	96,733	98,980	101,697	103,886	106,878	110,583	113,006	115,310	117,687	119,807	122,901	126,016	128,812	131,319	133,489	136,191
23,121	23,455	23,937	24,137	24,226	24,503	24,995	25,121	25,613	26,098	26,378	26,577	26,788	27,060	27,360	27,615	27,848	28,001	28,183	28,347	28,499
39,208	39,640	40,022	40,468	40,873	41,303	41,288	41,559	41,762	42,022	42,201	42,740	42,979	43,091	43,239	43,460	43,818	43,968	44,092	44,179	44,347
7,788	7,893	7,966	8,110	8,225	8,334	8,406	8,492	8,538	8,717	8,801	8,857	8,886	9,016	9,120	9,199	9,269	9,332	9,389	9,474	9,537
8,529	8,690	8,690	8,809	9,067	9,096	9,171	9,236	9,422	9,498	9,606	9,685	9,712	9,746	9,773	9,845	9,942	9,972	10,020	10,056	10,107
48,675	49,451	50,127	50,867	51,746	52,255	52,634	51,918	53,114	54,029	54,764	55,415	56,001	57,293	58,701	59,993	61,246	62,416	63,378	64,448	65,779
40,460	41,482	42,145	42,846	43,369	43,794	44,447	45,226	45,711	46,301	47,009	47,656	48,207	48,894	49,847	50,621	51,309	51,898	52,497	53,249	53,980
596	594	595	595	595	595	596	601	603	606	607	607	610	616	619	625	634	636	637	644	647
16,170	16,504	16,898	17,250	17,577	17,703	18,357	18,585	18,956	19,244	19,552	19,698	20,015	20,108	20,804	21,146	21,478	21,588	21,988	22,237	22,603
2,534	2,595	2,626	2,626	2,684	2,699	2,731	2,769	2,803	2,839	2,839	2,906	2,933	2,990	3,054	3,111	3,139	3,139	3,189	3,220	3,260
102,686	105,444	107,796	110,304	112,017	113,195	114,306	115,833	117,455	118,917	120,260	121,234	122,848	123,830	124,759	125,915	126,890	127,757	129,139	129,936	130,561
29,936	30,409	30,901	31,376	31,715	32,078	32,437	33,068	33,558	34,211	34,574	34,830	35,237	35,712	36,096	36,578	36,997	37,397	37,623	38,033	38,337
8,539	8,958	8,958	8,958	9,218	9,218	9,337	9,337	9,719	9,719	9,719	10,011	10,011	10,170	10,170	10,393	10,393	10,393	10,650	10,650	10,812
8,286	8,426	8,571	8,571	8,571	8,951	9,077	9,184	9,464	9,704	9,704	10,046	10,185	10,410	10,705	10,977	11,287	11,287	11,476	11,708	11,883
36,627	37,048	37,163	37,292	37,914	38,159	38,602	38,907	38,907	39,682	39,916	40,452	40,857	41,133	41,562	41,989	42,486	42,816	43,050	43,612	44,030
90,084	90,899	91,662	92,675	93,271	93,693	94,220	94,895	95,512	96,301	96,965	100,805	101,163	101,592	102,063	102,557	103,132	103,436	103,626	103,889	104,156
44,424	45,495	46,313	47,152	47,687	48,423	49,709	50,988	52,015	52,778	53,327	54,175	54,982	55,858	56,770	57,482	57,973	58,404	58,904	59,465	60,197
1,948	1,948	2,013	2,074	2,109	2,109	2,189	2,226	2,282	2,325	2,349	2,377	2,418	2,418	2,446	2,482	2,570	2,570	2,588	2,606	2,667
53,510	53,913	54,365	54,679	54,881	55,104	55,608	56,014	56,621	56,884	57,397	57,532	57,731	58,035	58,241	63,539	63,983	64,413	64,701	64,998	65,182
19,005	19,845	19,845	20,573	21,960	22,464	22,464	23,531	23,531	24,850	25,208	25,208	25,508	25,870	26,273	27,501	27,886	28,224	28,523	28,869	29,316
11,340	11,558	11,752	11,988	12,167	12,291	12,492	12,673	12,795	12,962	13,147	13,327	13,575	13,767	14,057	14,253	14,442	14,553	14,734	14,913	15,187
12,624	13,005	13,252	13,458	13,731	14,044	14,372	14,790	15,230	15,501	15,752	16,021	16,322	16,560	16,560	16,769	17,270	17,768	17,768	18,483	18,483
479	479	479	479	479	479	485	493	505	505	515	523	525	539	539	540	540	548	548	554	561
20,860	21,618	22,725	23,222	23,964	24,140	24,628	25,412	26,488	27,673	28,589	29,263	29,889	30,777	31,966	33,255	34,625	35,555	36,484	37,160	38,171
2,229	2,317	2,365	2,418	2,457	2,422	2,439	2,481	2,520	2,554	2,577	2,625	2,646	2,679	2,706	2,745	2,816	2,861	2,880	2,901	2,941
11,425	11,662	11,989	12,134	12,355	12,619	12,976	13,261	13,654	13,905	14,101	14,345	14,611	14,866	15,117	15,379	15,543	15,634	15,752	15,883	16,025
3,935	4,014	4,089	4,149	4,197	4,231	4,286	4,386	4,492	4,545	4,651	4,685	4,749	4,795	4,876	4,953	5,019	5,043	5,079	5,132	5,178
151,472	152,719	153,104	154,154	155,092	155,764	156,628	157,185	158,844	159,608	160,445	160,918	161,545	162,068	162,530	163,336	163,893	164,164	164,497	164,796	165,346
6,472	6,625	6,795	6,943	7,026	7,130	7,252	7,364	7,493	7,624	7,689	7,800	8,024	8,140	8,353	8,672	8,800	8,940	9,062	9,105	9,250
7,271	7,401	7,696	7,786	7,888	7,998	8,113	8,225	8,350	8,495	8,610	8,688	8,830	8,951	9,090	9,285	9,460	9,649	9,805	10,030	10,164
353623	355621	362859	358844	365405	366638	367994	369460	365323	366802	373022	368777	375224	371019	372046	373097	379322	380089	380737	381505	382136
30,167	30,794	31,408	31,911	32,477	33,006	33,439	33,915	34,566	35,034	35,513	35,984	36,413	36,792	37,282	37,758	38,111	38,476	38,837	39,162	39,575
5,503	5,503	5,543	5,691	5,860	5,974	6,051	6,104	6,238	6,280	6,280	6,347	6,427	6,582	6,707	6,820	6,921	6,921	7,072	7,163	7,218
3,817	3,864	3,888	3,927	3,949	3,967	4,038	4,086	4,131	4,185	4,243	4,302	4,335	4,399	4,474	4,570	4,662	4,808	4,922	4,988	5,060
66,258	66,983	67,713	68,186	68,637	69,417	70,042	70,735	71,415	71,926	72,282	72,894	73,405	73,942	74,385	75,086	75,592	75,943	76,436	76,846	77,313
13,571	13,736	13,952	14,065	14,065	14,210	14,353	14,494	14,635	14,819	14,928	14,991	15,112	15,219	15,325	15,441	15,441	15,441	15,642	15,691	15,756
9,379	9,638	9,895	10,096	10,178	10,416	10,623	10,788	11,131	11,394	11,861	12,148	12,415	12,651	13,005	13,453	13,916	14,286	14,800	15,228	15,759
4,250	4,356	4,468	4,563	4,586	4,653	4,710	4,793	4,866	4,960	4,993	5,034	5,067	5,162	5,247	5,277	5,367	5,438	5,471	5,523	5,604
19,073	19,510	19,909	20,269	20,731	21,089	21,441	21,822	22,236	22,720	23,159	23,709	24,543	24,990	25,289	25,690	26,248	26,561	27,129	27,759	28,061
52,268	53,449	54,509	55,348	55,971	56,560	57,921	59,776	61,006	62,338	64,287	64,880	66,568	68,271	69,920	71,613	73,553	74,978	75,616	77,253	79,757
7,981	8,179	8,331	8,500	8,584	8,658	8,815	9,137	9,429	9,688	9,944	10,101	10,353	10,737	11,015	11,252	11,939	12,117	12,429	12,704	13,048
34,950	35,749	36,244	37,727	39,342	40,249	41,401	42,533	43,611	44,607	45,398	46,239	46,905	47,856	48,532	49,397	50,681	51,251	51,738	52,177	52,647
950	952	954	956	962	967	971	974	975	977	981	983	988	990	1,025	1,027	1,045	1,063	1,075	1,084	1,094
19,117	19,265	19,585	19,828	20,065	20,181	20,406	20,764	21,071	21,349	21,702	21,977	22,157	22,484	22,729	22,993	23,422	23,729	24,041	24,354	24,642
13,885	14,396	14,877	15,277	15,584	15,863	16,462	16,974	17,707	18,230	18,403	18,543	18,917	19,400	19,892	20,249	20,571	20,835	21,038	21,308	21,593
1,603	1,705	1,729	1,771	1,782	1,854	1,899	1,935	1,972	1,989	2,010	2,028	2,056	2,077	2,102	2,119	2,136	2,144	2,161	2,179	2,193
801	803	813	838	843	850	860	876	891	898	903	910	912	915	921	933	939	947	960	970	980
1568448	1592599	1618757	1634010	1658896	1675258	1694864	1716078	1734040	1757522	1783638	1798330	1823220	1837803	1857872	1886794	1915712	1933560	1951096	1968331	1988646

6/11/2020	6/12/2020	6/13/2020	6/14/2020	6/15/2020	6/16/2020	6/17/2020	6/18/2020	6/19/2020	6/20/2020	6/21/2020	6/22/2020	6/23/2020	6/24/2020	6/25/2020	6/26/2020	6/27/2020	6/28/2020	6/29/2020	6/30/2020
610	625	654	661	664	676	696	708	723	743	755	761	778	792	816	836	854	883	904	940
22,845	23,710	25,368	26,287	26,713	26,912	27,327	28,214	29,017	29,549	30,021	30,477	31,234	32,086	33,232	34,220	35,105	36,350	37,203	38,064
10,816	11,547	12,095	12,501	12,917	13,191	13,606	13,928	14,631	15,142	15,561	16,083	16,678	17,375	18,062	18,740	19,310	19,818	20,257	20,777
31,264	32,918	34,458	35,691	36,705	39,097	40,924	43,443	46,689	49,798	52,390	54,586	58,179	59,974	63,030	66,458	70,051	73,908	74,533	79,215
139,281	141,983	145,643	148,855	151,452	153,560	157,015	161,099	165,416	169,309	173,824	178,054	183,073	190,222	195,571	200,461	206,433	211,243	216,550	222,917
28,647	28,822	29,017	29,130	29,299	29,442	29,673	29,901	30,187	30,349	30,539	30,705	30,893	31,155	31,479	31,796	32,022	32,307	32,511	32,715
44,461	44,689	44,994	45,088	45,235	45,349	45,429	45,440	45,557	45,715	45,755	45,782	45,899	45,913	45,994	46,059	46,206	46,303	46,362	46,514
9,589	9,654	9,709	9,767	9,799	9,818	9,847	9,903	9,952	9,984	10,020	10,058	10,094	10,128	10,159	10,185	10,216	10,248	10,292	10,327
10,173	10,229	10,264	10,340	10,403	10,444	10,500	10,611	10,611	10,775	10,822	10,847	10,889	10,980	11,017	11,107	11,253	11,416	11,376	11,510
67,456	69,341	71,589	73,650	75,388	78,128	80,676	83,854	87,643	91,670	95,139	98,047	101,303	106,743	111,724	120,574	130,092	138,567	143,805	149,781
54,973	55,783	56,801	57,681	58,414	59,078	60,030	60,912	62,009	63,809	64,701	65,928	67,675	69,381	71,095	72,995	74,985	77,210	79,417	81,291
654	663	680	688	696	674	678	718	746	760	769	771	775	766	782	796	802	828	829	842
22,973	23,348	23,717	23,926	24,077	24,179	24,461	24,854	25,275	25,496	25,957	26,051	26,372	26,705	27,257	27,685	28,012	28,489	28,782	28,947
3,302	3,353	3,399	3,399	3,462	3,540	3,632	3,743	3,871	4,006	4,006	4,254	4,402	4,645	4,865	5,148	5,319	5,319	5,752	6,117
131,327	132,059	132,732	133,404	133,877	134,500	135,046	135,639	136,470	137,104	137,762	138,224	138,825	139,540	140,434	141,344	142,130	142,776	143,514	144,238
38,748	39,146	39,543	39,909	40,430	40,786	41,013	41,438	41,746	42,061	42,423	42,633	42,871	43,140	43,655	44,140	44,575	44,930	45,228	45,594
10,812	11,047	11,047	11,047	11,419	11,419	11,681	11,681	12,059	12,059	12,059	12,465	12,465	12,970	12,970	13,538	13,538	13,538	14,443	14,443
11,945	12,166	12,445	12,445	12,647	12,829	12,995	13,197	13,454	13,630	13,750	13,839	14,141	14,363	14,617	14,859	15,167	15,232	15,347	15,624
44,472	44,995	46,283	46,619	47,172	47,706	48,634	48,634	48,515	49,385	49,778	50,239	51,595	52,477	53,415	54,769	54,769	56,236	57,081	58,095
104,667	105,059	105,395	105,603	105,690	105,885	106,151	106,422	106,650	106,936	107,061	107,210	107,439	107,611	107,837	108,070	108,443	108,667	108,768	108,882
60,613	61,305	61,701	62,032	62,409	62,969	63,229	63,548	63,956	64,306	64,603	65,007	65,337	65,777	66,115	66,450	66,777	67,254	67,559	67,918
2,667	2,721	2,757	2,810	2,819	2,819	2,836	2,878	2,938	2,938	2,957	2,971	2,994	3,071	3,070	3,102	3,191	3,221	3,252	3,294
65,449	65,672	65,836	66,054	66,085	66,269	66,497	66,798	67,097	67,545	67,711	67,957	68,197	68,555	68,989	69,329	69,679	69,946	70,223	70,728
29,316	29,795	30,172	30,693	30,882	31,296	31,296	31,675	32,467	32,920	33,227	33,469	33,763	33,763	34,616	34,616	35,549	35,546	35,861	36,716
15,390	15,585	15,810	15,810	16,189	16,414	16,625	16,908	17,201	17,590	18,005	18,143	18,577	18,868	19,421	19,914	20,261	20,575	21,043	21,551
18,483	19,348	19,348	19,799	20,152	20,641	20,641	20,641	20,641	20,641	20,641	22,287	22,898	23,424	24,516	25,531	25,892	26,567	27,247	27,900
573	588	588	609	612	630	630	666	698	716	717	734	766	803	829	852	852	919	967	1,018
39,481	41,249	42,676	44,119	45,102	45,853	46,855	48,188	49,840	51,389	52,801	53,605	54,453	56,174	57,183	58,818	60,537	62,142	63,484	64,670
2,980	3,016	3,058	3,080	3,101	3,124	3,166	3,193	3,226	3,251	3,288	3,313	3,320	3,362	3,393	3,421	3,458	3,495	3,539	3,576
16,315	16,513	16,633	16,725	16,851	17,031	17,226	17,415	17,591	17,707	17,810	17,957	18,092	18,221	18,346	18,524	18,775	18,899	19,042	19,177
5,209	5,251	5,299	5,318	5,345	5,364	5,436	5,450	5,450	5,518	5,544	5,558	5,571	5,598	5,638	5,638	5,671	5,747	5,760	5,782
165,816	166,164	166,603	166,881	167,103	167,426	167,703	168,107	168,496	168,834	169,142	169,415	169,734	169,892	170,196	170,584	170,873	171,182	171,272	171,667
9,367	9,526	9,621	9,723	9,845	9,933	10,065	10,153	10,260	10,430	10,565	10,694	10,838	10,990	11,192	11,408	11,619	11,809	11,982	12,147
10,417	10,678	10,964	11,173	11,297	11,658	11,860	12,094	12,515	13,160	13,434	13,764	14,228	14,592	15,085	15,472	16,576	17,397	18,131	18,684
382913	378606	379459	380052	380734	386489	386990	387645	388330	389213	389737	390326	390894	391510	392292	393041	393728	394250	394667	395294
40,004	40,424	40,848	41,148	41,576	42,010	42,422	43,122	43,731	44,262	44,808	45,537	46,127	46,759	47,651	48,638	49,455	50,309	51,046	51,789
7,378	7,496	7,640	8,231	8,251	8,437	8,665	8,925	9,378	9,730	10,062	10,545	10,759	10,759	11,948	11,975	12,367	12,675	12,977	13,217
5,237	5,377	5,535	5,636	5,820	6,098	6,218	6,366	6,572	6,750	6,937	7,083	7,274	7,444	7,568	7,818	8,094	8,341	8,485	8,656
77,999	78,462	78,462	79,121	79,483	79,818	80,236	80,762	81,266	81,730	82,186	82,186	83,191	83,770	84,370	84,991	85,496	85,988	86,606	87,242
15,862	15,947	15,947	15,947	16,093	16,164	16,213	16,269	16,337	16,337	16,337	16,459	16,533	16,606	16,640	16,661	16,661	16,661	16,764	16,813
16,441	17,170	17,955	18,795	19,378	19,990	20,556	21,548	22,631	23,786	24,693	25,701	26,613	27,897	29,022	30,335	31,939	33,320	34,644	36,399
5,665	5,742	5,833	5,898	5,928	5,966	6,050	6,109	6,158	6,225	6,297	6,326	6,353	6,419	6,479	6,535	6,626	6,681	6,716	6,764
28,538	29,126	29,541	30,432	31,160	31,830	32,143	32,829	34,017	34,446	35,102	35,553	36,303	37,235	38,034	39,444	40,172	40,172	42,297	43,509
81,583	83,680	86,011	87,854	89,108	93,206	96,335	99,851	103,305	107,735	111,601	114,881	120,370	125,921	131,917	137,624	143,371	148,723	153,011	159,986
13,429	13,789	14,184	14,469	14,826	15,141	15,539	16,199	16,826	17,400	17,825	18,135	18,660	19,185	19,816	20,481	20,986	21,450	22,263	22,746
53,211	53,869	54,506	54,886	55,331	55,775	56,238	56,793	57,443	57,994	58,465	58,994	59,514	59,946	60,570	61,247	61,736	62,189	62,787	62,787
1,109	1,119	1,125	1,127	1,128	1,130	1,129	1,135	1,144	1,147	1,159	1,163	1,164	1,184	1,191	1,198	1,200	1,202	1,208	1,208
24,779	25,171	25,538	25,834	26,158	26,531	26,784	27,192	27,601	28,225	28,680	28,870	29,386	29,869	30,367	30,855	31,404	31,752	32,253	32,824
21,926	24,722	25,031	25,295	25,480	25,789	26,079	26,542	26,852	27,294	27,587	27,838	28,127	28,593	29,105	29,668	30,227	30,707	31,033	31,662
2,217	2,249	2,274	2,290	2,322	2,341	2,376	2,418	2,468	2,500	2,543	2,571	2,593	2,629	2,694	2,730	2,782	2,832	2,870	2,905
1,009	1,027	1,050	1,060	1,079	1,089	1,114	1,144	1,173	1,179	1,197	1,230	1,254	1,282	1,326	1,368	1,392	1,417	1,450	1,487
2010391	2032524	2057838	2079592	2098106	2126444	2149166	2176902	2208829	2241178	2268753	2295286	2329463	2367064	2407590	2452048	2496628	2537636	2573393	2616949

7/1/2020	7/2/2020	7/3/2020	7/4/2020	7/5/2020	7/6/2020	7/7/2020	7/8/2020	7/9/2020	7/10/2020	7/11/2020	7/12/2020	7/13/2020	7/14/2020	7/15/2020	7/16/2020	7/17/2020	7/18/2020	7/19/2020	7/20/2020	7/21/2020
978	1,017	1,063	1,111	1,138	1,166	1,184	1,226	1,272	1,323	1,385	1,479	1,539	1,579	1,631	1,693	1,733	1,795	1,874	1,949	2,041
38,981	40,111	41,865	42,862	44,909	44,909	45,821	46,991	49,205	50,546	51,947	53,587	55,590	57,308	59,113	61,143	63,153	65,234	67,011	68,950	70,426
21,197	22,075	22,622	23,209	23,814	24,253	24,512	25,246	26,052	26,803	27,864	28,367	28,939	29,733	30,297	31,114	31,762	32,533	33,228	33,927	34,665
84,092	87,425	91,858	94,553	98,089	101,441	105,094	108,614	112,671	116,892	119,930	122,467	123,824	128,097	131,354	134,613	138,523	141,265	143,624	145,183	148,683
232,657	240,195	248,235	254,745	260,155	271,684	277,774	289,468	296,499	304,297	312,344	320,804	329,162	336,508	347,634	356,178	366,164	375,363	384,692	391,538	400,769
33,029	33,352	33,612	33,866	34,065	34,257	34,664	35,116	35,525	36,191	36,591	36,913	37,242	37,686	38,155	38,726	39,344	39,788	40,142	40,566	41,059
46,572	46,646	46,717	46,717	46,717	46,976	47,033	47,108	47,209	47,287	47,287	47,287	47,510	47,530	47,636	47,750	47,893	47,893	47,893	48,055	48,096
10,365	10,390	10,435	10,447	10,482	10,515	10,569	10,642	10,679	10,743	10,801	10,847	10,906	10,946	11,026	11,076	11,115	11,194	11,261	11,339	11,427
11,739	11,731	11,923	12,128	12,348	12,469	12,525	12,610	12,751	12,857	12,978	12,879	12,969	13,050	13,114	13,337	13,429	13,429	13,519	13,746	13,792
156,288	166,303	175,718	187,090	197,076	203,376	210,594	220,476	229,367	240,710	250,984	266,119	278,667	287,789	297,876	311,640	323,002	333,201	345,612	355,899	365,244
84,237	87,709	90,493	93,319	95,516	97,064	100,470	103,890	106,727	111,211	114,401	116,926	120,569	123,963	127,834	131,275	135,183	139,872	143,123	145,575	148,988
851	870	894	915	939	946	980	1,001	1,036	1,058	1,097	1,115	1,138	1,158	1,190	1,209	1,232	1,245	1,273	1,287	1,310
29,458	30,209	30,430	31,061	31,353	31,657	32,029	32,509	33,243	33,896	34,647	35,171	35,529	35,850	36,294	37,132	37,722	38,041	38,723	39,224	39,473
6,370	6,593	6,994	7,370	7,733	8,052	8,539	8,969	9,428	9,928	10,505	10,902	11,402	11,718	12,445	13,133	13,752	14,302	14,873	15,266	15,822
145,066	145,066	146,872	147,734	148,373	148,987	149,574	150,554	151,572	152,899	154,094	155,048	155,931	156,638	157,825	159,082	160,509	161,785	162,750	163,923	164,878
45,952	46,387	46,915	47,432	48,008	48,331	48,626	49,063	49,575	50,300	51,079	51,612	52,037	52,685	53,370	54,080	54,813	55,654	56,571	57,206	57,916
14,990	14,990	15,919	15,919	15,919	16,901	16,901	17,618	17,618	18,611	18,611	18,611	20,058	20,058	20,933	20,933	21,965	21,965	21,965	23,334	23,334
15,842	16,079	16,376	16,376	16,376	17,152	17,519	17,919	18,245	18,670	19,121	19,389	19,653	20,223	20,677	21,083	21,605	22,184	23,161	23,414	24,060
60,178	61,561	63,289	63,289	65,226	66,327	68,263	70,151	71,994	74,636	76,803	78,122	79,827	82,042	84,131	86,411	88,590	88,590	91,706	94,892	96,583
109,143	109,338	109,628	109,838	109,974	110,137	110,338	110,602	110,897	111,110	111,398	111,597	111,827	112,130	112,347	112,581	112,879	113,238	113,534	113,789	114,033
68,423	68,961	69,341	69,632	69,904	70,396	70,861	71,447	71,910	72,467	73,109	73,527	74,260	75,016	75,664	76,371	77,206	78,131	78,685	79,545	80,172
3,328	3,328	3,397	3,397	3,423	3,440	3,460	3,486	3,499	3,499	3,539	3,558	3,566	3,578	3,598	3,636	3,644	3,646	3,711	3,711	3,722
71,089	71,678	71,678	72,581	72,941	73,269	73,900	74,551	75,063	75,685	76,370	76,776	77,198	77,864	78,913	79,839	80,593	81,338	81,868	82,395	83,059
37,210	37,624	37,624	38,136	38,569	39,133	39,133	40,136	40,767	41,571	41,571	42,772	43,170	43,170	44,347	45,013	45,470	46,204	46,204	47,457	47,961
21,927	22,283	22,830	23,215	23,436	23,856	24,629	25,204	25,999	26,661	27,133	27,443	27,890	28,826	29,714	30,422	31,290	32,248	33,094	33,624	34,762
28,770	28,770	30,674	30,900	31,257	31,257	32,214	33,591	34,622	34,622	36,287	36,680	37,542	37,542	39,798	40,828	40,829	42,639	43,889	45,524	47,071
1,083	1,123	1,128	1,167	1,249	1,249	1,371	1,466	1,569	1,593	1,677	1,841	1,843	1,952	2,218	2,342	2,366	2,471	2,533	2,621	2,712
66,514	64,670	70,241	70,241	72,983	74,529	75,875	77,310	79,349	81,331	83,793	85,701	87,528	89,484	91,266	93,426	95,477	97,958	99,778	101,046	102,861
3,615	3,657	3,722	3,779	3,816	3,849	3,898	3,971	4,070	4,154	4,243	4,334	4,442	4,565	4,668	4,792	4,907	5,019	5,126	5,207	5,367
19,310	19,452	19,660	19,827	19,929	20,046	20,201	20,425	20,623	20,777	20,998	21,172	21,399	21,717	21,979	22,134	22,361	22,481	22,583	22,847	23,190
5,802	5,822	5,822	5,857	5,897	5,914	5,932	5,952	5,973	5,991	6,024	6,054	6,068	6,091	6,113	6,139	6,165	6,188	6,203	6,249	6,262
171,928	172,356	172,742	173,033	173,402	173,611	173,878	174,039	174,270	174,628	174,959	175,298	175,522	175,915	176,278	176,501	176,551	176,814	176,783	176,963	177,256
12,276	12,520	12,776	13,063	13,256	13,507	13,727	14,017	14,251	14,549	14,773	15,028	15,291	15,514	15,841	16,138	16,456	16,736	16,971	17,215	17,517
19,328	19,964	20,946	21,803	22,646	23,137	24,020	24,587	25,139	26,144	26,862	27,894	28,744	29,832	30,673	32,120	33,503	34,689	35,977	36,925	37,741
395982	396869	397675	398327	398828	399411	399925	400719	401345	402110	402861	403474	404051	405017	405876	406594	407334	407972	408495	408730	409845
52,865	54,166	55,257	56,183	57,151	57,956	58,904	60,181	61,331	62,856	64,214	65,592	66,853	67,995	69,311	70,601	72,280	73,822	74,932	76,168	77,215
13,853	14,217	14,217	15,645	15,776	16,063	16,501	17,357	18,036	18,680	19,779	19,934	20,401	20,922	21,832	22,907	23,535	25,056	25,265	25,581	26,326
8,931	9,294	9,636	9,930	10,230	10,395	10,605	10,819	11,188	11,454	11,851	12,170	12,438	12,805	13,081	13,509	13,802	14,149	14,579	14,847	15,139
88,074	88,741	89,375	89,854	90,304	91,299	92,148	92,867	93,876	94,689	95,266	95,742	96,671	97,665	98,446	99,478	100,241	101,027	101,738	102,765	103,396
16,853	16,941	16,991	16,991	16,991	16,991	17,154	17,204	17,243	17,312	17,312	17,312	17,487	17,487	17,640	17,711	17,793	17,793	17,793	17,904	17,986
37,919	39,701	41,532	43,386	44,847	46,380	47,352	48,909	50,691	52,419	54,699	56,648	58,168	60,389	62,245	64,083	66,060	67,612	69,986	71,445	73,337
6,826	6,893	6,978	7,028	7,063	7,105	7,163	7,242	7,336	7,401	7,454	7,499	7,524	7,572	7,652	7,694	7,789	7,862	7,906	7,943	8,019
45,315	46,890	48,712	50,140	51,431	52,155	53,514	55,986	57,591	59,546	61,006	61,960	65,274	66,788	69,061	71,540	73,819	76,336	78,115	79,754	81,944
168,062	175,977	183,532	191,790	195,239	200,557	210,585	220,564	230,346	240,111	250,462	258,658	264,313	275,058	282,365	292,656	307,572	317,730	325,030	332,434	341,739
23,386	23,921	24,571	25,002	25,500	26,004	26,706	27,480	28,054	28,963	29,605	30,177	30,722	31,196	31,664	32,587	33,392	34,045	34,682	35,084	35,648
63,203	63,735	64,393	65,748	66,102	66,740	67,375	67,988	68,931	69,782	70,670	71,642	72,443	73,527	74,431	75,433	76,373	77,430	78,375	79,371	80,393
1,210	1,227	1,238	1,238	1,251	1,251	1,254	1,256	1,272	1,283	1,283	1,296	1,301	1,305	1,318	1,325	1,334	1,338	1,350	1,360	1,366
33,435	34,151	34,778	35,247	35,898	36,985	37,420	37,941	38,581	39,218	39,218	40,656	41,757	42,304	43,046	44,313	45,067	46,026	46,946	47,743	48,575
32,225	32,809	33,431	34,207	34,740	35,230	35,765	36,410	37,210	38,099	39,080	39,877	40,382	41,349	42,197	43,139	44,068	45,099	45,948	46,675	47,836
2,979	3,053	3,126	3,205	3,335	3,442	3,505	3,707	3,826	3,983	4,146	4,244	4,313	4,407	4,557	4,657	4,783	4,922	5,042	5,142	5,199
1,514	1,550	1,582	1,606	1,634	1,675	1,711	1,740	1,774	1,790	1,839	1,862	1,903	1,951	1,985	2,026	2,069	2,108	2,126	2,187	2,238
2671220	2720390	2781463	2832139	2877238	2923432	2973695	3038325	3097300	3163336	3225950	3286063	3344783	3405494	3472659	3544143	3618497	3685460	3748248	3805524	3868453

7/22/2020	7/23/2020	7/24/2020	7/25/2020	7/26/2020	7/27/2020	7/28/2020	7/29/2020	7/30/2020	7/31/2020	8/1/2020	8/2/2020	8/3/2020	8/4/2020	8/5/2020	8/6/2020	8/7/2020	8/8/2020	8/9/2020	8/10/2020	8/11/2020
2,132	2,192	2,249	2,338	2,524	2,622	2,729	2,797	2,878	2,990	3,136	3,280	3,341	3,394	3,449	3,484	3,536	3,613	3,711	3,775	3,821
71,886	74,344	76,136	78,130	79,294	81,270	82,530	83,782	85,906	87,867	89,349	91,444	92,661	93,847	94,819	96,592	98,484	100,353	101,334	103,020	103,851
35,246	36,259	37,249	37,981	38,623	39,447	40,181	40,968	41,759	42,511	43,173	43,810	44,597	45,381	46,293	47,028	48,039	48,811	49,383	50,028	50,411
150,609	152,944	156,301	160,041	162,014	163,827	165,934	168,273	170,798	174,010	177,022	178,467	179,497	180,505	182,203	183,647	185,053	186,107	186,923	187,523	188,737
413,576	425,616	435,334	445,400	453,659	460,550	466,550	475,305	485,502	493,588	500,130	509,162	514,901	519,427	524,722	529,980	538,416	545,787	554,160	561,911	574,411
41,698	42,314	42,980	43,789	44,336	44,565	45,314	45,796	46,204	46,809	47,267	47,716	47,968	48,394	48,988	49,436	49,893	50,324	50,660	51,039	51,441
48,223	48,232	48,776	48,776	48,776	48,983	49,077	49,540	49,670	49,810	49,810	49,810	50,062	50,110	50,225	50,245	50,320	50,320	50,320	50,567	50,684
11,529	11,571	11,649	11,717	11,780	11,858	11,945	11,999	12,057	12,126	12,205	12,274	12,313	12,398	12,443	12,518	12,589	12,653	12,753	12,807	12,896
13,924	13,924	14,202	14,175	14,406	14,476	14,602	14,689	14,689	14,877	14,877	14,949	15,055	15,296	15,365	15,365	15,502	15,575	15,634	15,634	15,699
374,920	385,091	397,470	409,585	418,844	427,698	436,867	446,251	456,105	465,030	474,621	481,668	486,426	491,773	497,181	504,768	512,421	520,846	527,036	531,188	536,981
152,302	156,588	161,401	165,188	167,953	170,843	175,052	178,323	182,286	186,352	190,012	193,177	195,435	197,948	201,713	204,895	209,004	213,427	216,596	219,025	222,588
1,339	1,389	1,449	1,516	1,576	1,603	1,647	1,750	1,871	1,989	2,067	2,110	2,311	2,442	2,603	2,741	2,919	3,138	3,270	3,408	3,523
40,000	40,635	41,271	41,671	42,199	42,554	42,782	43,280	44,039	44,582	44,981	45,492	45,845	46,045	46,659	47,362	47,865	48,286	48,789	49,074	49,329
16,322	16,736	17,264	17,827	18,177	18,694	19,222	19,679	20,246	20,721	21,114	21,344	21,675	22,234	22,707	23,399	23,922	24,495	24,671	25,100	25,595
166,476	168,100	169,699	171,125	172,666	173,897	174,973	176,366	178,138	180,118	181,757	183,224	184,522	185,993	187,752	189,705	191,808	193,998	195,380	196,699	198,248
58,673	59,602	60,598	61,520	62,372	62,907	63,678	64,299	65,253	66,154	67,122	67,857	68,433	69,255	69,975	71,015	72,254	73,287	74,328	74,992	75,862
24,104	24,104	25,109	25,109	25,109	26,172	26,172	26,870	26,870	27,812	27,812	27,812	28,876	28,876	29,717	29,717	30,638	30,638	30,638	31,730	31,730
24,540	25,147	25,931	26,764	27,079	27,601	28,126	28,727	29,386	30,151	30,723	31,185	31,508	32,197	32,741	33,254	33,796	34,758	34,982	35,254	35,793
99,354	101,650	103,734	103,734	107,574	109,917	111,038	112,773	114,481	116,280	116,280	119,747	120,846	124,461	125,943	127,246	128,746	128,746	131,399	131,961	133,125
114,320	114,647	114,985	115,274	115,637	115,926	116,182	116,684	117,098	117,612	118,040	118,458	118,657	119,203	119,643	119,874	120,291	120,711	121,040	121,315	121,707
80,836	81,766	83,054	83,748	84,876	85,524	86,285	87,177	88,346	89,365	90,274	91,144	91,854	92,426	93,005	93,806	94,581	95,503	96,258	96,843	97,384
3,737	3,758	3,757	3,814	3,832	3,838	3,866	3,888	3,910	3,937	3,958	3,970	3,975	3,992	3,997	4,015	4,026	4,042	4,051	4,050	4,069
83,730	84,431	85,072	85,622	86,661	87,173	87,958	88,974	89,781	90,574	91,332	91,761	92,374	93,175	93,893	94,656	95,470	96,191	96,726	97,306	98,213
48,721	49,490	50,291	50,291	51,803	52,281	52,947	53,692	54,464	55,188	55,947	55,947	57,162	57,779	58,640	59,185	60,101	60,898	61,516	61,839	61,839
36,063	37,700	39,352	40,709	41,927	43,050	44,823	46,750	48,834	50,323	51,258	51,840	52,887	54,080	55,321	56,383	57,379	57,379	58,927	59,954	60,935
48,053	49,663	51,097	52,304	52,304	52,957	55,804	57,579	57,579	59,881	59,881	61,125	62,199	62,199	63,444	64,400	66,646	66,646	67,173	67,649	68,293
2,909	3,037	3,247	3,342	3,381	3,381	3,667	3,780	3,954	3,965	4,081	4,193	4,314	4,429	4,593	4,744	4,888	4,952	5,017	5,017	5,289
105,001	106,893	108,995	111,092	112,713	114,338	116,087	117,850	120,194	122,148	123,878	125,219	126,532	128,161	129,288	131,267	132,812	134,766	136,218	136,844	137,895
5,493	5,614	5,736	5,876	5,986	6,141	6,227	6,301	6,469	6,602	6,660	6,785	6,933	7,057	7,177	7,327	7,508	7,596	7,713	7,885	7,970
23,486	23,818	24,174	24,395	24,618	24,889	25,157	25,422	25,766	26,211	26,391	26,702	26,956	27,178	27,489	27,821	28,104	28,245	28,432	28,696	29,030
6,295	6,318	6,375	6,415	6,436	6,441	6,500	6,513	6,544	6,583	6,613	6,634	6,660	6,693	6,719	6,742	6,779	6,818	6,831	6,840	6,861
177,645	177,887	178,345	178,858	179,363	179,812	180,295	180,766	180,970	181,660	182,029	182,350	182,614	182,970	183,327	183,701	184,061	184,429	184,773	185,031	185,475
17,828	18,163	18,475	18,788	19,042	19,502	19,791	20,136	20,388	20,600	20,796	21,016	21,130	21,340	21,566	21,773	21,965	22,115	22,315	22,444	22,643
38,877	40,140	40,140	42,037	43,067	44,055	45,151	46,021	47,170	48,312	49,306	50,437	51,422	52,423	53,068	53,919	54,757	55,644	56,456	57,198	57,745
410560	411140	412062	412562	413091	413834	414479	415209	415960	416633	417273	417730	418352	419131	419752	420523	421137	421834	422349	422824	423587
78,742	80,186	81,746	83,184	84,073	85,177	86,497	87,893	89,626	91,159	92,087	93,031	93,963	95,106	96,305	97,471	98,675	99,969	100,848	101,731	102,826
27,541	28,221	29,348	30,360	33,582	34,981	34,990	37,060	38,291	39,105	40,418	40,910	41,199	41,423	43,414	44,325	45,272	46,159	46,652	47,067	47,908
15,394	15,713	16,104	16,492	16,758	17,088	17,416	17,721	18,131	18,493	18,817	19,097	19,366	19,699	19,978	20,225	20,636	21,010	21,272	21,488	21,774
104,358	105,571	106,625	107,425	108,264	109,384	110,218	111,078	112,048	112,936	112,936	113,590	114,155	115,009	115,714	116,521	117,279	118,092	118,852	119,453	120,281
18,062	18,148	18,224	18,224	18,224	18,515	18,725	18,800	18,950	19,022	19,022	19,022	19,246	19,390	19,481	19,611	19,738	19,738	19,738	19,934	20,053
75,042	76,606	78,607	80,008	81,199	82,417	84,109	85,846	87,572	89,016	90,599	91,788	92,951	94,190	95,472	96,797	98,219	99,460	100,435	101,159	102,130
8,077	8,143	8,200	8,305	8,395	8,444	8,492	8,641	8,685	8,764	8,867	8,955	9,020	9,079	9,168	9,273	9,371	9,477	9,605	9,663	9,713
84,417	86,987	89,078	90,796	93,936	96,489	99,044	100,822	102,871	105,959	108,184	109,627	110,636	112,441	114,098	116,350	118,782	120,585	122,712	123,914	124,915
351,618	361,125	369,826	375,846	381,656	385,923	394,265	403,307	412,107	420,946	430,485	430,485	442,014	451,181	459,887	467,485	474,524	481,483	486,362	490,817	500,620
36,254	36,889	37,681	38,064	38,502	38,915	39,337	39,942	40,258	40,797	41,380	41,759	42,153	42,544	43,096	43,606	44,067	44,090	44,714	45,006	45,399
81,237	82,364	83,609	84,567	86,072	86,994	87,993	88,904	89,888	90,801	91,782	93,106	94,251	95,049	95,867	97,882	99,189	100,086	100,750	101,746	102,521
1,366	1,377	1,385	1,396	1,400	1,402	1,405	1,406	1,407	1,414	1,421	1,426	1,426	1,431	1,436	1,445	1,448	1,459	1,462	1,462	1,478
49,247	50,009	50,824	51,849	52,635	53,321	54,205	54,995	55,803	55,803	57,541	58,172	58,715	59,379	60,084	60,917	61,587	62,523	63,072	63,647	64,151
48,583	49,669	50,727	51,715	52,680	53,281	54,064	54,988	56,079	56,934	58,058	58,990	59,401	60,171	61,110	61,985	63,028	64,213	64,835	65,356	66,123
5,461	5,550	5,695	5,821	5,960	6,054	6,173	6,326	6,422	6,642	6,735	6,854	6,973	7,051	7,159	7,277	7,433	7,563	7,694	7,754	7,875
2,288	2,347	2,405	2,446	2,475	2,520	2,589	2,628	2,686	2,726	2,769	2,808	2,848	2,884	2,923	2,958	3,000	3,013	3,050	3,042	3,073
3938094	4009808	4084043	4148011	4209509	4263531	4323160	4388566	4456389	4523888	4582276	4629459	4678610	4728239	4781612	4836661	4897958	4951851	4999815	5039709	5094500

8/12/2020	8/13/2020	8/14/2020	8/15/2020	8/16/2020	8/17/2020	8/18/2020	8/19/2020	8/20/2020	8/21/2020	8/22/2020	8/23/2020
3,881	3,963	4,073	4,156	4,259	4,309	4,371	4,438	4,520	4,588	4,677	4,741
104,786	105,557	106,309	107,580	108,433	109,004	110,361	111,478	112,449	113,632	114,532	115,992
51,114	51,766	52,392	51,992	52,665	53,077	53,487	54,216	54,765	55,652	56,199	56,574
189,443	190,794	191,721	192,654	193,537	194,005	194,920	195,557	196,280	196,899	197,895	198,103
586,056	593,141	601,075	613,689	621,562	628,031	632,667	638,831	644,751	650,336	656,892	663,669
51,756	52,129	52,538	52,838	53,176	53,370	53,631	53,901	54,230	54,586	54,883	55,133
50,706	50,782	50,897	50,897	50,897	51,267	51,255	51,314	51,432	51,519	51,519	51,519
12,959	13,024	13,118	13,159	13,220	13,273	13,325	13,354	13,409	13,469	13,534	13,590
15,967	15,967	16,396	16,451	16,536	16,536	16,643	16,718	16,770	16,831	16,895	16,942
545,040	551,232	557,337	563,628	567,375	570,024	573,811	577,891	582,407	587,023	591,283	594,287
226,153	228,668	231,895	235,168	237,030	238,861	241,677	243,982	246,741	249,630	252,222	253,949
3,699	4,022	4,241	4,510	4,697	4,839	4,965	5,176	5,372	5,587	5,864	6,111
49,806	50,336	50,935	51,793	52,433	52,829	52,969	53,280	54,533	55,006	55,735	56,275
26,133	26,631	27,173	27,477	27,660	27,942	28,326	28,696	29,120	29,369	29,662	29,853
199,893	201,727	204,023	205,851	207,413	209,186	210,926	213,221	215,053	217,346	219,702	221,595
76,522	77,565	78,632	79,676	80,415	81,006	81,847	82,336	83,277	84,317	85,317	85,932
32,547	32,547	33,885	33,885	33,885	35,167	35,167	35,890	35,890	36,856	36,856	36,856
36,945	37,686	38,298	38,930	39,315	39,691	40,299	40,926	41,626	42,265	43,066	43,529
134,304	135,439	136,737	136,737	137,918	138,485	139,125	139,903	140,821	141,720	141,720	142,943
121,936	122,255	124,081	123,199	123,593	123,842	124,063	124,415	124,728	125,159	125,268	125,268
98,160	98,875	99,693	100,212	100,715	101,235	101,649	102,229	102,899	103,523	104,102	104,669
4,089	4,115	4,144	4,168	4,197	4,213	4,235	4,253	4,285	4,316	4,335	4,356
98,689	99,856	100,724	101,782	102,259	102,749	103,403	104,091	104,618	104,618	106,044	106,808
62,296	63,723	64,413	65,162	65,716	65,716	66,061	67,308	68,133	68,133	69,584	70,298
62,530	63,797	65,270	66,397	67,475	68,623	69,417	70,675	71,733	72,964	74,257	75,075
69,374	69,986	70,930	71,755	72,136	73,207	73,207	75,449	75,449	77,268	77,894	78,405
5,403	5,539	5,541	5,659	5,779	5,847	5,847	5,956	6,214	6,216	6,376	6,490
139,061	140,824	142,170	143,706	144,952	145,516	146,779	147,932	149,904	151,912	153,641	155,113
8,171	8,322	8,444	8,587	8,647	8,782	8,968	9,242	9,474	9,736	9,876	10,000
29,244	29,660	29,988	30,241	30,372	30,563	30,825	31,040	31,348	31,626	31,780	31,889
6,887	6,921	6,964	6,980	6,988	7,004	7,017	7,036	7,050	7,071	7,092	7,107
185,938	186,594	187,164	187,442	187,455	187,767	188,098	188,427	188,527	188,817	189,236	189,494
22,816	22,987	23,160	23,302	23,408	23,500	23,579	23,749	23,951	24,095	24,302	24,396
58,270	58,856	59,955	60,814	61,511	62,171	62,854	63,248	63,804	64,697	65,334	65,866
424329	424955	425315	426386	426909	427270	427897	428558	429087	429727	430408	430885
104,248	105,426	106,557	107,674	108,287	109,062	109,923	110,881	112,003	113,046	114,165	114,802
48,700	49,505	50,384	51,387	51,387	52,468	53,264	54,132	54,993	56,079	57,113	57,686
22,022	22,300	22,613	23,018	23,744	23,451	23,676	23,870	24,165	24,421	24,710	24,937
121,130	122,121	122,950	123,800	124,460	124,844	125,579	126,149	126,940	127,633	128,429	129,048
20,129	20,240	20,355	20,355	20,335	20,572	20,692	20,795	20,871	21,022	21,022	21,022
102,974	103,909	104,841	105,882	106,497	106,953	107,672	108,411	109,320	110,378	111,295	111,988
9,815	9,897	10,024	10,118	10,274	10,360	10,443	10,566	10,691	10,884	11,135	11,276
126,393	128,511	130,458	131,747	133,708	134,744	135,778	137,800	139,175	140,844	142,083	143,937
506,820	513,575	520,593	528,838	535,042	542,950	550,232	557,256	562,559	567,580	573,139	577,537
45,722	46,155	46,628	46,942	47,248	47,487	47,785	48,260	48,761	49,088	49,416	49,728
103,623	104,838	105,753	106,691	107,421	108,283	109,019	109,882	110,860	112,072	112,072	113,630
1,478	1,484	1,501	1,509	1,515	1,527	1,530	1,533	1,537	1,541	1,553	1,557
64,702	65,339	66,139	66,885	67,461	67,721	68,264	68,689	69,389	69,779	70,595	71,012
66,654	67,622	68,681	69,547	70,246	70,715	71,424	72,134	72,893	73,751	74,726	75,205
8,008	8,151	8,274	8,457	8,564	8,632	8,731	8,801	8,982	9,066	9,185	9,272
3,086	3,119	3,183	3,227	3,286	3,331	3,363	3,430	3,468	3,524	3,543	3,579
5150407	5202433	5258565	5312940	5354013	5392007	5431046	5477305	5521257	5567217	5612163	5649928

eTable 4. COVID-19 death

Date/Total Death	1/22/2020	1/23/2020	1/24/2020	1/25/2020	1/26/2020	1/27/2020	1/28/2020	1/29/2020	1/30/2020	1/31/2020	2/1/2020	2/2/2020	2/3/2020	2/4/2020	2/5/2020	2/6/2020	2/7/2020	2/8/2020	2/9/2020	2/10/2020
AK	0	0	0	0	0	0	0	0	0	0	0	0	0	0	0	0	0	0	0	0
AL	0	0	0	0	0	0	0	0	0	0	0	0	0	0	0	0	0	0	0	0
AR	0	0	0	0	0	0	0	0	0	0	0	0	0	0	0	0	0	0	0	0
AZ	0	0	0	0	0	0	0	0	0	0	0	0	0	0	0	0	0	0	0	0
CA	0	0	0	0	0	0	0	0	0	0	0	0	0	0	0	0	0	0	0	0
CO	0	0	0	0	0	0	0	0	0	0	0	0	0	0	0	0	0	0	0	0
CT	0	0	0	0	0	0	0	0	0	0	0	0	0	0	0	0	0	0	0	0
DC	0	0	0	0	0	0	0	0	0	0	0	0	0	0	0	0	0	0	0	0
DE	0	0	0	0	0	0	0	0	0	0	0	0	0	0	0	0	0	0	0	0
FL	0	0	0	0	0	0	0	0	0	0	0	0	0	0	0	0	0	0	0	0
GA	0	0	0	0	0	0	0	0	0	0	0	0	0	0	0	0	0	0	0	0
HI	0	0	0	0	0	0	0	0	0	0	0	0	0	0	0	0	0	0	0	0
IA	0	0	0	0	0	0	0	0	0	0	0	0	0	0	0	0	0	0	0	0
ID	0	0	0	0	0	0	0	0	0	0	0	0	0	0	0	0	0	0	0	0
IL	0	0	0	0	0	0	0	0	0	0	0	0	0	0	0	0	0	0	0	0
IN	0	0	0	0	0	0	0	0	0	0	0	0	0	0	0	0	0	0	0	0
KS	0	0	0	0	0	0	0	0	0	0	0	0	0	0	0	0	0	0	0	0
KY	0	0	0	0	0	0	0	0	0	0	0	0	0	0	0	0	0	0	0	0
LA	0	0	0	0	0	0	0	0	0	0	0	0	0	0	0	0	0	0	0	0
MA	0	0	0	0	0	0	0	0	0	0	0	0	0	0	0	0	0	0	0	0
MD	0	0	0	0	0	0	0	0	0	0	0	0	0	0	0	0	0	0	0	0
ME	0	0	0	0	0	0	0	0	0	0	0	0	0	0	0	0	0	0	0	0
MI	0	0	0	0	0	0	0	0	0	0	0	0	0	0	0	0	0	0	0	0
MN	0	0	0	0	0	0	0	0	0	0	0	0	0	0	0	0	0	0	0	0
MO	0	0	0	0	0	0	0	0	0	0	0	0	0	0	0	0	0	0	0	0
MS	0	0	0	0	0	0	0	0	0	0	0	0	0	0	0	0	0	0	0	0
MT	0	0	0	0	0	0	0	0	0	0	0	0	0	0	0	0	0	0	0	0
NC	0	0	0	0	0	0	0	0	0	0	0	0	0	0	0	0	0	0	0	0
ND	0	0	0	0	0	0	0	0	0	0	0	0	0	0	0	0	0	0	0	0
NE	0	0	0	0	0	0	0	0	0	0	0	0	0	0	0	0	0	0	0	0
NH	0	0	0	0	0	0	0	0	0	0	0	0	0	0	0	0	0	0	0	0
NJ	0	0	0	0	0	0	0	0	0	0	0	0	0	0	0	0	0	0	0	0
NM	0	0	0	0	0	0	0	0	0	0	0	0	0	0	0	0	0	0	0	0
NV	0	0	0	0	0	0	0	0	0	0	0	0	0	0	0	0	0	0	0	0
NY	0	0	0	0	0	0	0	0	0	0	0	0	0	0	0	0	0	0	0	0
OH	0	0	0	0	0	0	0	0	0	0	0	0	0	0	0	0	0	0	0	0
OK	0	0	0	0	0	0	0	0	0	0	0	0	0	0	0	0	0	0	0	0
OR	0	0	0	0	0	0	0	0	0	0	0	0	0	0	0	0	0	0	0	0
PA	0	0	0	0	0	0	0	0	0	0	0	0	0	0	0	0	0	0	0	0
RI	0	0	0	0	0	0	0	0	0	0	0	0	0	0	0	0	0	0	0	0
SC	0	0	0	0	0	0	0	0	0	0	0	0	0	0	0	0	0	0	0	0
SD	0	0	0	0	0	0	0	0	0	0	0	0	0	0	0	0	0	0	0	0
TN	0	0	0	0	0	0	0	0	0	0	0	0	0	0	0	0	0	0	0	0
TX	0	0	0	0	0	0	0	0	0	0	0	0	0	0	0	0	0	0	0	0
UT	0	0	0	0	0	0	0	0	0	0	0	0	0	0	0	0	0	0	0	0
VA	0	0	0	0	0	0	0	0	0	0	0	0	0	0	0	0	0	0	0	0
VT	0	0	0	0	0	0	0	0	0	0	0	0	0	0	0	0	0	0	0	0
WA	0	0	0	0	0	0	0	0	0	0	0	0	0	0	0	0	0	0	0	0
WI	0	0	0	0	0	0	0	0	0	0	0	0	0	0	0	0	0	0	0	0
WV	0	0	0	0	0	0	0	0	0	0	0	0	0	0	0	0	0	0	0	0
WY	0	0	0	0	0	0	0	0	0	0	0	0	0	0	0	0	0	0	0	0

2/11/2020	2/12/2020	2/13/2020	2/14/2020	2/15/2020	2/16/2020	2/17/2020	2/18/2020	2/19/2020	2/20/2020	2/21/2020	2/22/2020	2/23/2020	2/24/2020	2/25/2020	2/26/2020	2/27/2020	2/28/2020	2/29/2020	3/1/2020
0	0	0	0	0	0	0	0	0	0	0	0	0	0	0	0	0	0	0	0
0	0	0	0	0	0	0	0	0	0	0	0	0	0	0	0	0	0	0	0
0	0	0	0	0	0	0	0	0	0	0	0	0	0	0	0	0	0	0	0
0	0	0	0	0	0	0	0	0	0	0	0	0	0	0	0	0	0	0	0
0	0	0	0	0	0	0	0	0	0	0	0	0	0	0	0	0	0	0	0
0	0	0	0	0	0	0	0	0	0	0	0	0	0	0	0	0	0	0	0
0	0	0	0	0	0	0	0	0	0	0	0	0	0	0	0	0	0	0	0
0	0	0	0	0	0	0	0	0	0	0	0	0	0	0	0	0	0	0	0
0	0	0	0	0	0	0	0	0	0	0	0	0	0	0	0	0	0	0	0
0	0	0	0	0	0	0	0	0	0	0	0	0	0	0	0	0	0	0	0
0	0	0	0	0	0	0	0	0	0	0	0	0	0	0	0	0	0	0	0
0	0	0	0	0	0	0	0	0	0	0	0	0	0	0	0	0	0	0	0
0	0	0	0	0	0	0	0	0	0	0	0	0	0	0	0	0	0	0	0
0	0	0	0	0	0	0	0	0	0	0	0	0	0	0	0	0	0	0	0
0	0	0	0	0	0	0	0	0	0	0	0	0	0	0	0	0	0	0	0
0	0	0	0	0	0	0	0	0	0	0	0	0	0	0	0	0	0	0	0
0	0	0	0	0	0	0	0	0	0	0	0	0	0	0	0	0	0	0	0
0	0	0	0	0	0	0	0	0	0	0	0	0	0	0	0	0	0	0	0
0	0	0	0	0	0	0	0	0	0	0	0	0	0	0	0	0	0	0	0
0	0	0	0	0	0	0	0	0	0	0	0	0	0	0	0	0	0	0	0
0	0	0	0	0	0	0	0	0	0	0	0	0	0	0	0	0	0	0	0
0	0	0	0	0	0	0	0	0	0	0	0	0	0	0	0	0	0	0	0
0	0	0	0	0	0	0	0	0	0	0	0	0	0	0	0	0	0	0	0
0	0	0	0	0	0	0	0	0	0	0	0	0	0	0	0	0	0	0	0
0	0	0	0	0	0	0	0	0	0	0	0	0	0	0	0	0	0	0	0
0	0	0	0	0	0	0	0	0	0	0	0	0	0	0	0	0	0	0	0
0	0	0	0	0	0	0	0	0	0	0	0	0	0	0	0	0	0	0	0
0	0	0	0	0	0	0	0	0	0	0	0	0	0	0	0	0	0	0	0
0	0	0	0	0	0	0	0	0	0	0	0	0	0	0	0	0	0	0	0
0	0	0	0	0	0	0	0	0	0	0	0	0	0	0	0	0	0	0	0
0	0	0	0	0	0	0	0	0	0	0	0	0	0	0	0	0	0	0	0
0	0	0	0	0	0	0	0	0	0	0	0	0	0	0	0	0	0	0	0
0	0	0	0	0	0	0	0	0	0	0	0	0	0	0	0	0	0	0	0
0	0	0	0	0	0	0	0	0	0	0	0	0	0	0	0	0	0	0	0
0	0	0	0	0	0	0	0	0	0	0	0	0	0	0	0	0	0	0	0
0	0	0	0	0	0	0	0	0	0	0	0	0	0	0	0	0	0	0	0
0	0	0	0	0	0	0	0	0	0	0	0	0	0	0	0	0	0	0	0
0	0	0	0	0	0	0	0	0	0	0	0	0	0	0	0	0	0	0	0
0	0	0	0	0	0	0	0	0	0	0	0	0	0	0	0	0	0	0	0
0	0	0	0	0	0	0	0	0	0	0	0	0	0	0	0	0	0	0	0
0	0	0	0	0	0	0	0	0	0	0	0	0	0	0	0	0	0	0	0
0	0	0	0	0	0	0	0	0	0	0	0	0	0	0	0	0	0	0	0
0	0	0	0	0	0	0	0	0	0	0	0	0	0	0	0	0	0	0	0
0	0	0	0	0	0	0	0	0	0	0	0	0	0	0	0	0	0	0	0
0	0	0	0	0	0	0	0	0	0	0	0	0	0	0	0	0	0	0	0
0	0	0	0	0	0	0	0	0	0	0	0	0	0	0	0	0	0	0	0
0	0	0	0	0	0	0	0	0	0	0	0	0	0	0	0	0	0	0	0
0	0	0	0	0	0	0	0	0	0	0	0	0	0	0	0	0	0	1	1
0	0	0	0	0	0	0	0	0	0	0	0	0	0	0	0	0	0	0	0
0	0	0	0	0	0	0	0	0	0	0	0	0	0	0	0	0	0	0	0
0	0	0	0	0	0	0	0	0	0	0	0	0	0	0	0	0	0	0	0

3/2/2020	3/3/2020	3/4/2020	3/5/2020	3/6/2020	3/7/2020	3/8/2020	3/9/2020	3/10/2020	3/11/2020	3/12/2020	3/13/2020	3/14/2020	3/15/2020	3/16/2020	3/17/2020	3/18/2020	3/19/2020	3/20/2020	3/21/2020	3/22/2020
0	0	0	0	0	0	0	0	0	0	0	0	0	0	0	0	0	0	0	0	0
0	0	0	0	0	0	0	0	0	0	0	0	0	0	0	0	0	0	0	0	0
0	0	0	0	0	0	0	0	0	0	0	0	0	0	0	0	0	0	0	0	0
0	0	0	0	0	0	0	0	0	0	0	0	0	0	0	0	0	0	1	1	2
0	0	0	1	1	1	1	2	2	3	4	4	4	5	7	10	14	18	24	27	32
0	0	0	0	0	0	0	0	0	0	0	0	0	1	1	2	2	2	4	5	6
0	0	0	0	0	0	0	0	0	0	0	0	0	0	0	0	1	3	4	5	8
0	0	0	0	0	0	0	0	0	0	0	0	0	0	0	0	0	0	1	1	2
0	0	0	0	0	0	0	0	0	0	0	0	0	0	0	0	0	0	0	0	0
0	0	0	0	0	0	0	2	2	2	2	2	3	4	5	7	8	9	11	12	13
0	0	0	0	0	0	0	0	0	0	1	1	1	1	1	1	6	13	14	20	25
0	0	0	0	0	0	0	0	0	0	0	0	0	0	0	0	0	0	0	0	0
0	0	0	0	0	0	0	0	0	0	0	0	0	0	0	0	0	0	0	0	0
0	0	0	0	0	0	0	0	0	0	0	0	0	0	0	0	0	0	0	0	0
0	0	0	0	0	0	0	0	0	0	0	0	0	0	0	1	1	4	5	6	9
0	0	0	0	0	0	0	0	0	0	0	0	0	0	1	2	2	2	3	4	6
0	0	0	0	0	0	0	0	0	0	1	1	1	1	1	1	1	1	1	2	2
0	0	0	0	0	0	0	0	0	0	0	0	0	0	0	0	1	2	2	3	3
0	0	0	0	0	0	0	0	0	0	0	0	0	2	3	4	7	10	14	20	29
0	0	0	0	0	0	0	0	0	0	0	0	0	0	0	0	0	0	1	1	5
0	0	0	0	0	0	0	0	0	0	0	0	0	0	0	0	1	1	2	3	3
0	0	0	0	0	0	0	0	0	0	0	0	0	0	0	0	0	0	0	0	0
0	0	0	0	0	0	0	0	0	0	0	0	0	0	0	0	1	1	3	5	8
0	0	0	0	0	0	0	0	0	0	0	0	0	0	0	0	0	0	0	1	1
0	0	0	0	0	0	0	0	0	0	0	0	0	0	0	0	1	1	3	3	3
0	0	0	0	0	0	0	0	0	0	0	0	0	0	0	0	0	1	1	1	1
0	0	0	0	0	0	0	0	0	0	0	0	0	0	0	0	0	0	0	0	0
0	0	0	0	0	0	0	0	0	0	0	0	0	0	0	0	0	0	0	0	0
0	0	0	0	0	0	0	0	0	0	0	0	0	0	0	0	0	0	0	0	0
0	0	0	0	0	0	0	0	0	0	0	0	0	0	0	0	0	0	0	0	0
0	0	0	0	0	0	0	0	0	0	0	0	0	0	0	0	0	0	0	0	0
0	0	0	0	0	0	0	0	1	1	1	1	1	2	2	3	5	9	11	16	20
0	0	0	0	0	0	0	0	0	0	0	0	0	0	0	0	0	0	0	0	0
0	0	0	0	0	0	0	0	0	0	0	0	0	0	1	1	1	1	2	2	2
0	0	0	0	0	0	0	0	0	0	0	0	0	6	6	12	22	34	44	64	87
0	0	0	0	0	0	0	0	0	0	0	0	0	0	0	0	0	0	1	3	3
0	0	0	0	0	0	0	0	0	0	0	0	0	0	0	0	0	1	1	1	2
0	0	0	0	0	0	0	0	0	0	0	0	0	1	1	1	3	3	3	4	5
0	0	0	0	0	0	0	0	0	0	0	0	0	0	0	0	1	1	1	2	2
0	0	0	0	0	0	0	0	0	0	0	0	0	0	0	0	0	0	0	0	0
0	0	0	0	0	0	0	0	0	0	0	0	0	0	1	1	1	1	3	3	3
0	0	0	0	0	0	0	0	0	1	1	1	1	1	1	1	1	1	1	1	1
0	0	0	0	0	0	0	0	0	0	0	0	0	0	0	0	0	0	1	1	2
0	0	0	0	0	0	0	0	0	0	0	0	0	0	0	1	2	3	5	5	5
0	0	0	0	0	0	0	0	0	0	0	0	0	0	0	0	0	0	0	0	1
0	0	0	0	0	0	0	0	0	0	0	0	0	1	2	2	2	2	2	2	6
0	0	0	0	0	0	0	0	0	0	0	0	0	0	0	0	0	2	2	2	2
6	9	10	10	11	16	18	21	24	29	31	37	40	42	42	47	66	74	83	94	95
0	0	0	0	0	0	0	0	0	0	0	0	0	0	0	0	0	0	3	4	4
0	0	0	0	0	0	0	0	0	0	0	0	0	0	0	0	0	0	0	0	0
0	0	0	0	0	0	0	0	0	0	0	0	0	0	0	0	0	0	0	0	0

3/23/2020	3/24/2020	3/25/2020	3/26/2020	3/27/2020	3/28/2020	3/29/2020	3/30/2020	3/31/2020	4/1/2020	4/2/2020	4/3/2020	4/4/2020	4/5/2020	4/6/2020	4/7/2020	4/8/2020	4/9/2020	4/10/2020	4/11/2020	4/12/2020
0	1	1	1	2	2	3	3	3	3	3	3	5	6	6	6	7	7	7	8	8
0	0	0	1	3	3	4	6	13	17	17	21	26	31	32	39	48	48	58	60	61
0	2	2	3	3	5	6	7	8	10	12	12	14	16	16	18	18	21	24	27	29
2	5	6	8	13	15	17	20	24	29	32	41	52	64	65	73	80	89	97	108	115
35	44	57	64	107	119	137	151	172	172	203	270	276	319	343	374	442	492	541	609	651
7	11	19	24	31	44	47	51	69	80	97	111	126	140	150	179	193	226	250	274	290
10	12	19	21	27	33	34	36	69	85	112	132	165	189	206	277	335	380	444	494	554
2	2	3	3	4	5	9	9	9	11	12	15	21	22	24	24	27	32	38	47	50
0	0	0	2	2	5	6	7	10	11	12	14	14	14	15	16	19	23	32	33	35
17	20	23	29	46	56	60	71	85	101	144	170	195	221	254	296	323	371	419	446	461
31	38	47	60	65	79	84	103	130	154	176	198	208	219	319	351	371	413	420	432	447
0	0	0	0	0	0	0	0	1	1	2	3	4	4	5	5	5	6	8	9	9
0	0	1	1	3	3	4	6	7	9	11	11	14	22	25	26	27	29	31	34	41
0	0	0	3	4	5	6	7	9	9	9	10	10	10	13	15	18	24	25	27	27
12	16	19	26	34	47	65	73	99	141	157	210	243	274	307	380	462	528	596	677	720
7	12	14	17	24	31	32	35	49	65	78	102	116	127	139	173	203	245	300	350	350
2	2	2	3	4	5	6	8	9	10	13	17	21	23	25	27	39	44	52	56	57
4	4	5	5	8	9	9	11	18	20	31	37	40	45	59	65	73	79	90	94	97
34	46	73	83	119	137	151	185	239	273	310	370	409	477	512	582	652	702	755	806	840
9	11	15	25	35	44	48	56	89	122	154	192	216	231	260	356	433	503	599	692	756
3	4	4	5	5	5	10	18	18	31	36	42	53	91	103	124	138	171	171	206	262
0	0	0	0	1	1	3	5	5	7	7	9	10	10	10	12	14	16	17	19	19
15	24	43	60	92	111	132	184	259	337	417	478	540	617	727	845	959	1,076	1,278	1,391	1,487
1	1	2	4	4	5	10	10	17	18	22	22	24	29	34	39	50	57	57	67	70
3	5	8	8	9	10	12	13	14	18	19	19	24	34	39	53	58	77	96	109	110
1	2	5	7	8	13	16	20	22	26	29	29	35	51	59	67	76	82	82	93	98
0	0	0	1	1	1	2	4	5	5	5	5	6	6	6	6	6	6	6	6	6
0	0	1	3	3	4	6	8	9	16	19	21	24	33	33	46	65	65	74	80	81
0	0	0	0	1	1	1	3	3	3	3	3	3	3	3	4	4	5	6	7	8
0	0	0	0	2	2	2	2	3	4	5	6	8	8	8	10	14	15	17	17	17
1	1	1	1	2	2	3	3	3	4	5	7	9	9	9	13	18	21	22	23	23
27	44	62	81	108	140	161	198	267	355	537	646	846	917	1,003	1,232	1,504	1,700	1,932	2,187	2,350
0	0	1	1	1	2	2	4	5	6	7	10	11	12	12	13	16	17	19	20	26
4	4	6	10	29	14	15	17	26	32	38	43	45	46	58	71	80	85	102	112	114
156	231	332	424	546	779	895	1069	1307	1672	1957	2375	2820	3489	5940	5489	6240	7451	7844	8627	9162
6	8	10	15	19	25	29	39	55	65	81	91	102	119	142	167	193	213	231	247	248
2	3	7	7	8	15	17	17	23	30	34	38	42	46	51	67	79	80	88	94	96
5	8	10	11	12	13	13	16	18	18	21	22	26	27	29	33	38	44	48	51	52
2	7	10	16	22	34	38	49	63	74	90	102	136	150	162	240	310	338	416	494	507
0	0	0	0	0	2	3	4	8	10	12	14	17	25	27	30	35	47	49	56	63
5	7	7	9	13	15	16	18	22	26	31	34	40	44	48	51	63	67	72	80	82
1	1	1	1	1	1	1	1	1	2	2	2	2	2	4	6	6	6	6	6	6
2	2	3	3	6	6	7	13	23	23	32	36	42	44	65	72	79	94	98	100	101
8	11	12	18	23	27	34	38	41	58	70	90	105	127	140	154	177	199	226	254	271
1	1	1	2	2	2	3	5	5	7	7	7	8	8	13	13	13	15	17	18	18
6	9	13	14	14	17	25	27	34	41	46	46	52	54	63	75	109	121	121	130	149
5	7	8	8	10	12	12	12	13	16	17	17	20	22	23	23	23	23	24	25	27
110	123	132	147	175	189	189	195	195	246	261	284	310	337	371	394	420	445	475	491	508
5	5	6	8	13	13	13	14	16	24	31	37	56	68	77	92	99	111	128	137	144
0	0	0	0	0	0	1	1	1	2	2	2	2	3	4	4	4	5	5	6	8
0	0	0	0	0	0	0	0	0	0	0	0	0	0	0	0	0	0	0	0	0

4/13/2020	4/14/2020	4/15/2020	4/16/2020	4/17/2020	4/18/2020	4/19/2020	4/20/2020	4/21/2020	4/22/2020	4/23/2020	4/24/2020	4/25/2020	4/26/2020	4/27/2020	4/28/2020	4/29/2020	4/30/2020	5/1/2020	5/2/2020	5/3/2020
8	9	9	9	9	9	9	9	9	9	9	9	9	9	9	9	9	9	9	9	9
62	73	75	82	96	113	165	147	180	187	198	209	213	220	228	243	260	280	289	288	290
30	32	33	37	40	41	40	42	43	42	45	47	48	49	50	52	59	61	64	72	76
122	131	142	150	169	177	184	187	208	229	249	266	273	275	275	293	254	320	330	348	362
687	758	821	890	985	1,072	1,072	1,208	1,268	1,354	1,469	1,562	1,651	1,710	1,755	1,809	1,887	1,982	2,073	2,171	2,215
308	329	357	373	391	411	421	448	485	507	551	672	670	679	706	736	766	777	820	832	842
602	608	868	971	1,036	1,086	1,127	1,331	1,423	1,544	1,639	1,764	1,862	1,924	2,012	2,089	2,168	2,257	2,339	2,436	2,495
52	67	72	81	86	91	96	105	112	127	139	153	165	178	185	190	205	224	231	240	251
41	43	46	52	61	67	72	72	89	92	100	100	112	125	137	144	152	152	159	168	177
499	571	614	668	726	748	774	823	867	927	987	1,046	1,055	1,074	1,088	1,171	1,218	1,268	1,314	1,364	1,399
481	526	579	624	661	670	691	782	821	838	873	899	897	912	944	1,025	1,054	1,111	1,167	1,174	1,178
9	9	9	9	9	9	10	10	12	12	12	13	14	14	16	16	16	16	16	16	17
43	49	53	60	64	74	75	79	83	90	96	107	112	118	127	136	148	162	170	175	184
33	39	41	41	43	44	45	48	51	54	54	54	56	56	58	60	60	63	63	64	64
794	868	948	1,072	1,134	1,259	1,290	1,349	1,468	1,565	1,688	1,795	1,874	1,933	1,983	2,125	2,215	2,355	2,457	2,559	2,618
350	387	436	477	519	545	562	569	630	661	706	741	872	901	932	992	1,065	1,114	1,175	1,229	1,246
62	71	76	80	88	88	92	100	107	110	112	111	117	118	120	124	125	129	130	131	134
104	115	122	129	137	144	148	154	171	185	191	200	205	208	213	225	235	240	248	248	253
884	1,013	1,103	1,156	1,213	1,267	1,296	1,328	1,405	1,532	1,599	1,601	1,644	1,729	1,740	1,801	1,845	1,905	1,970	1,950	2,012
844	957	1,108	1,245	1,404	1,560	1,706	1,809	1,961	2,182	2,360	2,556	2,730	2,899	3,003	3,153	3,405	3,562	3,716	3,846	4,004
302	413	459	494	494	534	582	652	698	748	798	798	875	945	1,016	1,078	1,140	1,192	1,192	1,251	1,317
19	20	24	27	29	32	34	35	36	39	44	47	50	50	51	51	53	55	55	56	57
1,602	1,768	1,921	2,093	2,226	2,307	2,391	2,468	2,700	2,813	2,977	3,084	3,273	3,315	3,407	3,567	3,670	3,789	3,866	4,020	4,049
79	87	90	101	115	121	143	160	179	179	221	221	244	272	301	319	343	343	371	395	428
114	133	147	152	165	175	176	177	189	208	218	262	273	274	288	314	318	329	337	351	352
111	122	129	140	140	152	169	183	183	201	209	209	221	229	239	250	250	282	281	291	310
7	7	7	8	8	10	10	12	13	14	14	14	14	14	16	15	16	16	16	16	16
86	108	117	131	152	164	172	179	213	242	253	269	289	299	306	342	354	378	399	420	422
8	9	9	9	9	9	10	13	13	14	15	15	16	17	19	19	19	19	23	24	25
18	20	21	24	24	28	28	33	38	42	47	50	53	56	55	55	68	70	73	76	78
23	27	32	34	37	38	41	42	42	48	51	53	60	60	60	60	66	72	81	84	86
2,443	2,805	3,156	3,518	3,840	4,070	4,202	4,377	4,753	5,063	5,368	5,617	5,863	5,938	6,044	6,442	6,770	7,228	7,538	7,742	7,871
31	36	36	44	51	53	55	58	65	71	78	84	93	99	104	110	112	123	131	139	151
120	130	137	142	151	155	157	164	170	179	197	203	206	213	213	232	237	252	254	257	262
8843	9522	14306	14724	15759	16274	17027	18367	19103	19256	20255	20400	20985	21504	21883	22275	22759	23211	23673	23826	24560
268	324	361	389	418	451	471	509	557	610	656	690	711	728	753	799	937	975	1,002	1,021	1,038
99	108	123	131	136	139	140	143	164	170	179	188	194	195	197	207	214	222	230	238	238
53	55	58	64	70	72	74	75	78	78	83	86	87	91	92	99	101	103	104	109	109
524	584	647	707	756	836	1,112	1,204	1,564	1,622	1,421	1,492	1,537	1,550	1,597	1,716	2,195	2,292	2,354	2,418	2,444
73	80	87	114	118	137	150	155	181	186	189	202	215	226	233	239	251	266	279	296	320
87	97	107	109	116	119	120	124	135	140	150	157	166	174	177	192	203	244	256	267	275
6	6	6	7	7	7	7	7	8	9	9	10	10	11	11	11	13	17	21	21	21
109	124	134	141	141	145	147	152	157	165	170	168	176	181	184	188	192	199	204	209	210
287	318	364	393	428	453	477	495	517	543	561	593	623	648	663	690	732	782	816	847	867
18	20	21	22	23	25	28	32	34	35	38	39	41	41	44	45	46	46	46	49	50
154	195	208	231	231	258	300	324	324	372	410	436	436	458	492	522	552	581	581	616	684
28	29	30	35	35	38	38	38	40	40	43	44	46	46	47	47	47	49	50	51	52
508	546	567	583	603	624	635	652	682	692	711	723	738	749	765	786	801	814	824	830	834
154	170	182	197	205	211	220	230	242	246	257	262	266	272	281	300	308	316	327	334	339
9	10	12	13	16	18	18	24	26	29	31	32	33	34	37	38	40	44	47	50	50
1	1	2	2	2	2	2	2	6	6	7	7	7	7	7	7	7	7	7	7	7

5/4/2020	5/5/2020	5/6/2020	5/7/2020	5/8/2020	5/9/2020	5/10/2020	5/11/2020	5/12/2020	5/13/2020	5/14/2020	5/15/2020	5/16/2020	5/17/2020	5/18/2020	5/19/2020	5/20/2020	5/21/2020	5/22/2020	5/23/2020	5/24/2020
9	9	10	10	10	10	10	10	10	10	10	10	10	10	10	10	10	10	10	10	10
300	323	343	370	386	394	394	424	433	451	475	484	487	488	496	504	524	534	543	551	554
80	83	87	88	88	90	91	94	95	97	98	98	98	98	100	102	107	110	113	115	116
362	395	426	450	517	532	536	542	562	594	624	651	679	680	686	704	747	763	775	799	800
2,254	2,317	2,412	2,504	2,585	2,678	2,745	2,770	2,847	2,934	3,032	3,108	3,204	3,261	3,302	3,334	3,436	3,542	3,630	3,708	3,774
851	903	921	944	960	967	971	986	1,006	1,059	1,090	1,150	1,192	1,215	1,224	1,257	1,299	1,309	1,323	1,327	1,330
2,556	2,633	2,718	2,797	2,874	2,932	2,967	3,008	3,041	3,125	3,219	3,285	3,339	3,408	3,449	3,472	3,529	3,582	3,637	3,675	3,693
258	264	277	285	304	311	323	328	336	350	358	368	375	383	392	400	407	412	418	427	432
182	187	193	213	213	224	225	237	247	260	271	271	286	297	304	310	317	322	324	324	326
1,399	1,471	1,539	1,600	1,669	1,715	1,735	1,735	1,779	1,827	1,875	1,917	1,964	1,973	1,997	2,052	2,096	2,144	2,190	2,233	2,237
1,211	1,258	1,327	1,336	1,362	1,404	1,404	1,442	1,464	1,505	1,527	1,557	1,592	1,606	1,642	1,668	1,689	1,758	1,808	1,814	1,827
17	17	17	17	17	17	17	17	17	17	17	17	17	17	17	17	17	17	17	17	17
188	207	219	231	243	252	265	271	289	306	318	336	346	351	364	372	386	407	424	445	454
64	65	66	67	67	67	67	70	69	69	72	73	73	73	74	77	77	77	79	79	79
2,662	2,838	2,974	3,111	3,241	3,349	3,406	3,459	3,601	3,792	3,928	4,058	4,129	4,177	4,234	4,379	4,225	4,607	4,715	4,790	4,856
1,264	1,326	1,377	1,414	1,447	1,490	1,508	1,540	1,578	1,619	1,646	1,691	1,741	1,751	1,765	1,824	1,864	1,913	1,941	1,964	1,976
136	137	144	147	152	157	157	158	158	164	164	172	172	172	173	173	178	178	185	185	185
261	275	283	294	298	304	304	311	321	326	328	332	334	334	346	366	376	386	391	391	391
2,064	2,115	2,094	2,208	2,227	2,267	2,286	2,308	2,347	2,381	2,417	2,448	2,479	2,491	2,563	2,581	2,608	2,629	2,668	2,683	2,690
4,090	4,212	4,420	4,552	4,702	4,840	4,979	5,108	5,141	5,315	5,649	5,592	5,705	5,797	5,862	5,938	6,066	6,148	6,228	6,304	6,372
1,390	1,437	1,437	1,560	1,614	1,644	1,683	1,756	1,809	1,866	1,911	1,957	1,992	1,992	2,080	2,123	2,159	2,159	2,243	2,277	2,302
61	62	62	62	63	64	64	65	66	66	69	70	70	71	73	73	73	75	75	77	78
4,135	4,179	4,250	4,343	4,393	4,526	4,551	4,584	4,674	4,714	4,787	4,825	4,880	4,891	4,915	5,017	5,060	5,129	5,158	5,223	5,228
428	455	485	534	534	578	578	591	646	638	691	692	730	739	757	786	786	851	861	861	878
358	377	396	418	449	472	482	488	524	542	562	576	589	594	605	616	631	661	671	676	681
342	374	374	409	422	430	435	457	466	480	493	511	521	528	555	570	580	595	615	625	635
16	16	16	16	16	16	16	16	16	16	16	16	16	16	16	16	16	16	16	16	16
430	452	477	507	527	544	547	550	577	597	615	641	652	659	661	691	702	716	728	737	744
25	25	31	31	33	35	35	36	38	40	40	42	42	43	44	45	49	51	52	52	53
78	82	86	90	92	96	98	100	103	107	113	119	123	123	125	132	138	143	147	147	150
86	92	111	114	121	131	133	133	142	150	151	159	171	172	172	182	190	199	204	208	209
7,910	8,244	8,549	8,801	8,952	9,116	9,255	9,310	9,508	9,702	9,946	10,138	10,249	10,356	10,435	10,586	10,747	10,843	10,985	11,081	11,133
156	162	169	172	181	191	200	208	219	231	242	253	259	265	270	276	283	294	302	308	317
275	285	286	293	301	306	317	317	332	342	339	354	349	365	379	373	394	397	387	392	410
24717	25014	25779	26120	26359	26697	26923	27184	27282	27448	27617	27755	28097	28168	28302	28484	28609	28663	28802	28978	29046
1,056	1,135	1,225	1,271	1,306	1,331	1,341	1,357	1,436	1,483	1,534	1,581	1,610	1,625	1,657	1,720	1,781	1,836	1,872	1,956	1,969
238	239	253	254	254	261	272	269	269	280	283	288	283	288	293	293	293	300	300	305	310
109	113	115	121	124	127	127	130	130	134	137	137	137	137	138	140	144	145	147	147	148
2,458	3,012	3,106	3,416	3,616	3,688	3,707	3,731	3,806	3,943	4,218	4,342	4,403	4,418	4,418	4,418	4,767	4,418	5,096	5,124	5,139
341	355	370	388	399	418	422	430	444	462	468	479	489	499	506	532	538	556	579	597	608
283	296	305	316	320	330	331	346	355	362	371	380	380	385	391	399	407	416	419	425	435
21	24	29	31	31	34	34	34	39	39	43	44	44	44	44	46	46	48	50	50	50
219	226	239	237	241	242	243	251	264	273	287	290	295	298	301	305	310	314	316	330	337
884	906	948	973	1,004	1,049	1,088	1,100	1,133	1,158	1,216	1,272	1,305	1,336	1,347	1,369	1,419	1,440	1,480	1,506	1,519
56	58	58	61	66	67	67	72	75	75	77	78	79	80	85	91	92	94	96	97	98
684	713	713	812	812	827	850	891	927	955	977	1,002	1,009	1,014	1,041	1,074	1,099	1,136	1,159	1,171	1,208
52	52	52	53	53	53	53	53	53	53	53	53	53	54	54	54	54	54	54	54	54
841	862	870	891	905	921	931	945	962	975	983	992	1,000	1,001	1,002	1,031	1,037	1,044	1,050	1,057	1,061
340	353	362	374	384	398	400	409	418	421	434	445	453	453	459	467	481	487	496	507	510
50	50	51	51	52	53	54	57	58	59	62	64	65	67	68	68	69	71	72	72	72
7	7	7	7	7	7	7	7	7	7	7	7	7	8	10	10	11	12	12	12	12

5/25/2020	5/26/2020	5/27/2020	5/28/2020	5/29/2020	5/30/2020	5/31/2020	6/1/2020	6/2/2020	6/3/2020	6/4/2020	6/5/2020	6/6/2020	6/7/2020	6/8/2020	6/9/2020	6/10/2020	6/11/2020	6/12/2020	6/13/2020	6/14/2020
10	10	10	10	10	10	10	10	10	10	10	10	10	10	10	11	11	11	12	12	12
568	579	584	594	613	620	632	646	653	653	653	674	688	692	728	729	744	755	769	774	776
117	119	120	125	132	133	133	133	136	142	151	152	154	154	155	161	165	171	176	177	179
806	807	831	857	885	903	906	917	941	981	996	1,012	1,042	1,044	1,047	1,070	1,095	1,127	1,144	1,183	1,186
3,795	3,814	3,884	3,973	4,068	4,156	4,213	4,251	4,286	4,361	4,422	4,485	4,559	4,626	4,653	4,697	4,776	4,881	4,943	4,989	5,063
1,331	1,350	1,392	1,421	1,436	1,443	1,445	1,458	1,474	1,494	1,512	1,524	1,527	1,527	1,543	1,553	1,573	1,583	1,595	1,598	1,599
3,742	3,769	3,803	3,826	3,868	3,912	3,944	3,964	3,972	3,989	4,007	4,038	4,055	4,071	4,084	4,097	4,120	4,146	4,159	4,186	4,201
440	440	445	453	460	462	466	468	470	473	475	479	483	489	491	495	499	502	506	511	515
337	344	345	356	361	366	369	373	375	386	388	390	398	398	410	413	414	414	418	422	423
2,252	2,259	2,319	2,364	2,413	2,447	2,451	2,460	2,530	2,566	2,607	2,660	2,688	2,700	2,712	2,765	2,801	2,848	2,877	2,925	2,931
1,830	1,874	1,909	1,973	1,971	2,003	2,052	2,074	2,102	2,123	2,147	2,174	2,160	2,180	2,208	2,285	2,329	2,375	2,418	2,446	2,451
17	17	17	17	17	17	17	17	17	17	17	17	17	17	17	17	17	17	17	17	17
459	475	492	506	523	531	534	549	561	574	580	592	598	604	614	624	631	640	643	650	652
79	81	82	82	82	82	82	83	83	83	83	83	83	83	83	85	85	86	87	87	87
4,884	4,923	5,083	5,186	5,270	5,330	5,390	5,412	5,525	5,621	5,736	5,795	5,864	5,904	6,102	6,196	6,273	6,363	6,441	6,470	6,489
1,984	2,004	2,030	2,068	2,110	2,125	2,134	2,142	2,197	2,207	2,231	2,258	2,292	2,303	2,316	2,339	2,355	2,380	2,396	2,413	2,422
188	188	205	205	208	208	208	217	217	222	222	232	232	232	236	236	240	240	243	243	243
391	394	400	409	418	431	431	439	442	450	458	466	470	470	472	477	484	493	497	499	499
2,690	2,701	2,722	2,740	2,766	2,785	2,791	2,801	2,835	2,870	2,883	2,912	2,925	2,936	2,944	2,957	2,968	2,987	2,996	3,004	3,014
6,416	6,473	6,547	6,640	6,718	6,768	6,846	7,035	7,085	7,012	7,201	7,235	7,289	7,316	7,353	7,408	7,454	7,492	7,538	7,576	7,624
2,333	2,392	2,428	2,466	2,509	2,532	2,552	2,597	2,641	2,668	2,702	2,740	2,749	2,776	2,811	2,844	2,875	2,900	2,926	2,939	2,947
79	79	84	85	89	89	89	94	95	95	95	98	99	99	99	100	100	100	100	100	101
5,240	5,266	5,334	5,372	5,406	5,463	5,491	5,516	5,553	5,570	5,585	5,855	5,891	5,895	5,912	5,943	5,955	5,985	5,990	6,013	6,016
908	908	942	1,006	1,006	1,050	1,060	1,060	1,082	1,097	1,126	1,181	1,197	1,208	1,228	1,267	1,280	1,280	1,305	1,314	1,335
685	686	696	707	738	771	772	773	783	786	789	799	809	809	819	840	848	860	872	879	879
652	671	692	710	723	734	739	768	782	794	794	803	817	837	837	868	868	868	889	889	895
16	17	17	17	17	17	17	17	17	17	17	18	18	18	18	18	18	18	18	18	19
764	766	794	827	859	877	886	898	921	939	960	966	992	996	1,006	1,029	1,053	1,064	1,092	1,104	1,109
54	54	56	57	59	60	61	61	65	66	66	71	72	72	72	72	73	74	74	74	74
150	153	163	164	170	170	170	178	181	187	186	186	188	188	188	191	195	212	216	216	216
210	214	223	232	238	242	245	245	256	265	273	278	283	286	286	294	301	308	315	318	320
11,144	11,191	11,339	11,401	11,531	11,634	11,698	11,721	11,770	11,880	11,970	12,049	12,106	12,176	12,214	12,303	12,377	12,443	12,489	12,589	12,625
320	325	329	335	344	351	356	362	367	375	383	387	392	396	400	404	410	420	426	431	435
412	396	402	427	415	417	438	421	420	445	429	452	437	438	461	444	448	476	462	481	464
29193	29289	29392	29500	29535	29612	29737	29766	29886	29918	30011	30066	30152	30216	30280	30351	30409	30481	30511	30565	30605
1,987	2,002	2,044	2,098	2,131	2,149	2,155	2,206	2,258	2,299	2,339	2,355	2,370	2,377	2,404	2,421	2,457	2,490	2,508	2,554	2,557
312	316	317	319	328	333	333	338	343	343	347	350	354	354	359	361	360	366	367	369	359
148	148	148	151	151	153	153	154	157	159	159	161	163	164	164	169	169	171	173	174	176
5,152	5,265	5,373	5,464	5,537	5,555	5,567	5,667	5,742	5,817	5,886	5,931	5,943	5,953	6,014	6,062	6,113	6,162	6,211	6,211	6,243
608	634	655	677	693	711	718	720	732	742	756	772	772	772	799	808	812	823	833	833	833
440	446	466	470	483	487	494	500	501	518	525	538	545	546	557	568	575	588	593	599	600
50	50	54	54	59	62	62	62	62	62	64	65	65	65	65	68	69	73	74	75	75
339	344	354	357	361	366	366	369	396	403	416	423	432	433	436	450	456	461	468	472	475
1,527	1,536	1,562	1,601	1,626	1,648	1,672	1,678	1,698	1,734	1,767	1,788	1,819	1,830	1,836	1,853	1,885	1,920	1,939	1,957	1,976
101	103	106	107	110	112	113	114	113	117	119	120	121	121	126	128	131	134	139	139	139
1,236	1,281	1,338	1,358	1,370	1,375	1,392	1,407	1,428	1,445	1,453	1,460	1,472	1,477	1,496	1,514	1,520	1,534	1,541	1,546	1,552
54	54	54	55	55	55	55	55	55	55	55	55	55	55	55	55	55	55	55	55	55
1,070	1,078	1,095	1,106	1,111	1,118	1,118	1,124	1,129	1,135	1,138	1,149	1,153	1,159	1,161	1,176	1,190	1,194	1,204	1,213	1,217
514	517	539	550	568	588	592	595	607	616	626	633	645	647	646	661	671	682	695	697	698
73	74	74	74	74	75	75	76	78	78	79	84	84	84	84	84	85	86	88	88	88
12	13	14	15	15	16	17	17	17	17	17	17	17	17	17	17	18	18	18	18	18

6/15/2020	6/16/2020	6/17/2020	6/18/2020	6/19/2020	6/20/2020	6/21/2020	6/22/2020	6/23/2020	6/24/2020	6/25/2020	6/26/2020	6/27/2020	6/28/2020	6/29/2020	6/30/2020	7/1/2020	7/2/2020	7/3/2020	7/4/2020	7/5/2020
12	12	12	12	12	12	12	12	12	12	12	14	14	14	14	14	14	14	15	16	16
781	785	792	810	823	838	839	843	870	890	895	910	923	924	931	947	969	985	1,006	1,007	1,009
182	188	197	208	214	224	225	227	237	240	242	249	259	264	265	270	277	279	281	286	287
1,194	1,219	1,239	1,271	1,312	1,338	1,339	1,342	1,384	1,463	1,490	1,535	1,579	1,588	1,588	1,632	1,720	1,757	1,788	1,805	1,809
5,089	5,121	5,208	5,290	5,360	5,424	5,495	5,515	5,580	5,632	5,733	5,812	5,872	5,905	5,936	5,980	6,090	6,163	6,263	6,313	6,331
1,605	1,617	1,631	1,638	1,643	1,647	1,647	1,651	1,665	1,667	1,669	1,673	1,674	1,676	1,682	1,690	1,697	1,701	1,701	1,701	1,701
4,204	4,210	4,219	4,226	4,238	4,251	4,260	4,263	4,277	4,287	4,298	4,307	4,311	4,316	4,320	4,322	4,324	4,326	4,335	4,335	4,335
515	520	523	527	530	531	533	535	537	541	543	546	548	550	551	551	553	554	555	557	559
424	426	431	433	433	435	435	504	505	507	507	507	507	507	507	509	510	510	512	512	512
2,938	2,993	3,018	3,061	3,104	3,144	3,161	3,173	3,238	3,281	3,327	3,366	3,390	3,419	3,447	3,505	3,550	3,617	3,684	3,702	3,731
2,494	2,529	2,575	2,605	2,636	2,642	2,643	2,648	2,687	2,698	2,745	2,770	2,776	2,778	2,784	2,805	2,827	2,849	2,856	2,857	2,860
17	17	17	17	17	17	17	17	17	17	17	17	17	18	18	18	18	18	19	19	19
655	669	673	680	681	682	685	686	688	692	695	704	704	705	708	714	717	718	721	721	721
88	88	88	89	89	89	89	89	89	90	90	90	91	91	91	92	92	92	113	93	93
6,507	6,579	6,666	6,718	6,784	6,829	6,851	6,875	6,911	6,974	7,014	7,048	7,074	7,089	7,103	7,124	7,152	7,152	7,215	7,224	7,230
2,433	2,447	2,475	2,491	2,516	2,536	2,540	2,553	2,569	2,578	2,586	2,595	2,616	2,619	2,624	2,640	2,650	2,662	2,681	2,687	2,693
245	245	247	247	254	254	254	259	259	261	261	264	264	264	270	270	272	272	277	277	277
505	512	518	520	522	524	526	526	537	538	546	553	554	558	560	565	572	581	585	585	585
3,018	3,042	3,062	3,062	3,084	3,104	3,105	3,117	3,134	3,152	3,164	3,190	3,190	3,199	3,199	3,221	3,238	3,255	3,278	3,278	3,288
7,647	7,665	7,734	7,770	7,800	7,828	7,858	7,874	7,890	7,938	7,963	8,013	8,041	8,060	8,095	8,054	8,081	8,132	8,149	8,172	8,183
2,982	2,996	3,016	3,030	3,052	3,066	3,074	3,092	3,108	3,129	3,142	3,157	3,168	3,175	3,190	3,205	3,212	3,223	3,236	3,243	3,246
101	101	102	102	102	102	102	102	102	103	103	103	104	105	105	105	105	105	107	107	109
6,017	6,034	6,036	6,061	6,067	6,087	6,090	6,097	6,109	6,114	6,133	6,134	6,153	6,157	6,161	6,193	6,198	6,212	6,212	6,218	6,218
1,344	1,357	1,357	1,376	1,406	1,412	1,416	1,425	1,432	1,432	1,446	1,446	1,460	1,470	1,470	1,482	1,495	1,503	1,503	1,508	1,511
880	882	909	946	948	955	956	961	966	975	982	990	996	997	998	1,015	1,017	1,022	1,026	1,027	1,028
915	939	938	938	938	938	938	978	989	1,011	1,016	1,035	1,039	1,059	1,073	1,082	1,092	1,092	1,107	1,111	1,114
19	20	20	20	20	20	20	21	21	21	22	22	22	22	22	22	22	23	23	23	23
1,118	1,154	1,168	1,175	1,197	1,212	1,220	1,223	1,251	1,271	1,290	1,303	1,318	1,322	1,325	1,343	1,373	1,343	1,392	1,392	1,396
77	74	74	75	76	76	77	77	78	78	78	87	78	79	79	79	80	80	89	80	80
220	231	234	240	244	244	244	249	256	257	260	266	267	267	269	274	276	282	284	284	284
320	326	330	331	331	339	339	339	343	347	357	357	365	367	367	371	373	375	375	376	381
12,676	12,727	12,769	12,800	12,835	12,857	12,870	12,895	12,949	12,995	14,872	14,914	14,948	14,975	14,992	15,035	15,080	15,107	15,164	15,189	15,211
440	447	452	456	464	466	469	469	476	480	485	489	491	492	493	497	500	503	511	513	513
483	467	491	493	499	507	508	510	513	516	517	520	522	522	536	529	533	548	551	553	557
30645	30709	30750	30804	30824	30880	30927	30956	30992	31020	31066	31095	31134	31744	31769	31784	31810	31831	31855	31891	31906
2,573	2,597	2,611	2,633	2,667	2,697	2,700	2,704	2,735	2,755	2,772	2,788	2,804	2,807	2,818	2,863	2,876	2,886	2,903	2,907	2,911
371	371	376	377	379	380	381	382	382	382	375	390	393	401	401	401	406	407	407	398	416
180	182	183	187	188	189	190	192	192	195	197	202	202	202	204	207	208	209	209	213	215
6,276	6,319	6,361	6,399	6,419	6,423	6,426	6,426	6,518	6,557	6,579	6,603	6,606	6,614	6,649	6,687	6,712	6,746	6,740	6,749	6,754
851	865	876	885	894	894	894	903	906	912	920	927	927	927	946	950	956	959	960	960	960
602	607	617	621	639	644	653	659	673	683	693	694	711	716	720	739	766	784	793	813	820
75	77	78	78	81	81	81	81	83	84	87	88	91	91	91	91	93	97	97	97	97
483	493	497	509	515	524	526	531	542	556	567	577	584	584	592	604	609	620	633	637	646
1,983	2,029	2,062	2,105	2,140	2,165	2,182	2,192	2,220	2,249	2,296	2,324	2,366	2,393	2,403	2,424	2,481	2,525	2,575	2,608	2,637
144	146	149	155	156	155	158	160	163	165	168	168	170	168	171	173	175	183	182	181	185
1,570	1,583	1,586	1,602	1,607	1,611	1,620	1,645	1,661	1,675	1,700	1,724	1,732	1,740	1,763	1,763	1,786	1,816	1,845	1,853	1,853
55	55	55	56	56	56	56	56	56	56	56	56	56	56	56	56	56	56	56	56	56
1,221	1,231	1,226	1,245	1,255	1,265	1,270	1,276	1,284	1,293	1,300	1,304	1,310	1,310	1,320	1,332	1,339	1,342	1,352	1,354	1,359
700	709	718	725	736	751	751	751	757	764	773	773	784	784	784	791	793	800	803	803	803
88	88	88	88	88	88	89	90	92	92	92	92	93	93	93	93	93	93	93	94	95
18	18	18	18	20	20	20	20	20	20	20	20	20	20	20	20	20	20	20	20	20

7/6/2020	7/7/2020	7/8/2020	7/9/2020	7/10/2020	7/11/2020	7/12/2020	7/13/2020	7/14/2020	7/15/2020	7/16/2020	7/17/2020	7/18/2020	7/19/2020	7/20/2020	7/21/2020	7/22/2020	7/23/2020	7/24/2020	7/25/2020	7/26/2020
16	17	17	17	17	17	17	17	17	17	17	17	18	18	18	18	19	19	19	20	20
1,009	1,032	1,057	1,067	1,103	1,114	1,121	1,124	1,166	1,211	1,230	1,263	1,286	1,287	1,292	1,307	1,363	1,399	1,439	1,456	1,473
292	301	305	309	313	319	321	323	331	335	341	353	357	357	363	374	380	386	394	399	401
1,810	1,927	1,963	2,038	2,082	2,151	2,237	2,245	2,337	2,434	2,492	2,583	2,730	2,761	2,784	2,918	2,974	3,063	3,142	3,286	3,305
6,337	6,488	6,562	6,711	6,851	6,945	7,017	7,040	7,087	7,227	7,345	7,475	7,595	7,685	7,694	7,755	7,870	8,027	8,186	8,337	8,416
1,691	1,696	1,704	1,706	1,724	1,725	1,725	1,727	1,738	1,744	1,745	1,751	1,752	1,752	1,758	1,763	1,771	1,786	1,790	1,794	1,794
4,338	4,338	4,343	4,348	4,348	4,348	4,348	4,371	4,372	4,380	4,389	4,396	4,396	4,396	4,406	4,406	4,406	4,410	4,413	4,413	4,413
561	561	564	568	568	568	568	568	568	571	574	577	578	578	579	580	580	581	581	581	581
514	515	517	518	518	518	517	518	521	521	521	523	523	523	525	527	529	529	578	579	579
3,778	3,841	3,953	4,009	4,102	4,197	4,242	4,277	4,409	4,521	4,677	4,805	4,895	4,982	5,072	5,206	5,345	5,518	5,653	5,777	5,854
2,878	2,899	2,922	2,930	2,965	2,996	3,001	3,026	3,054	3,091	3,104	3,132	3,168	3,173	3,176	3,254	3,335	3,360	3,442	3,495	3,498
19	19	19	19	19	19	19	22	22	22	22	23	24	24	24	24	25	26	26	26	26
723	725	735	740	743	748	750	754	757	767	778	784	787	793	797	802	808	818	823	826	827
94	94	98	100	101	102	102	102	103	110	114	118	119	119	122	126	135	138	144	146	146
7,236	7,273	7,309	7,329	7,345	7,369	7,388	7,394	7,419	7,427	7,452	7,465	7,483	7,488	7,494	7,517	7,540	7,560	7,577	7,589	7,590
2,698	2,717	2,732	2,739	2,748	2,756	2,760	2,762	2,775	2,785	2,795	2,803	2,820	2,822	2,825	2,846	2,863	2,880	2,884	2,895	2,903
280	280	282	282	284	284	284	288	288	299	299	299	299	299	307	307	308	308	326	326	326
593	602	608	612	620	622	625	629	635	645	650	658	667	670	671	674	677	684	691	696	700
3,296	3,319	3,339	3,355	3,380	3,403	3,416	3,423	3,445	3,461	3,485	3,509	3,509	3,543	3,572	3,608	3,670	3,686	3,715	3,715	3,763
8,198	8,213	8,243	8,268	8,296	8,310	8,325	8,330	8,340	8,368	8,380	8,402	8,419	8,431	8,433	8,450	8,468	8,484	8,498	8,510	8,529
3,266	3,275	3,288	3,303	3,310	3,319	3,325	3,334	3,341	3,347	3,359	3,368	3,377	3,382	3,402	3,405	3,409	3,422	3,433	3,440	3,447
110	110	111	111	111	114	114	114	114	114	115	117	117	117	118	118	118	118	118	119	119
6,221	6,251	6,262	6,271	6,285	6,313	6,314	6,321	6,326	6,330	6,348	6,355	6,364	6,366	6,373	6,382	6,388	6,395	6,400	6,400	6,400
1,514	1,514	1,528	1,533	1,537	1,537	1,542	1,548	1,548	1,564	1,573	1,578	1,581	1,581	1,588	1,592	1,601	1,606	1,611	1,611	1,616
1,028	1,042	1,046	1,051	1,064	1,069	1,069	1,083	1,093	1,103	1,113	1,121	1,130	1,129	1,132	1,143	1,159	1,179	1,178	1,182	1,197
1,114	1,158	1,204	1,215	1,215	1,249	1,250	1,273	1,272	1,308	1,333	1,332	1,357	1,358	1,389	1,423	1,436	1,463	1,480	1,495	1,495
23	23	25	27	28	29	31	32	34	34	37	37	37	37	39	40	42	45	46	46	47
1,398	1,420	1,441	1,461	1,479	1,499	1,503	1,510	1,552	1,568	1,588	1,606	1,629	1,634	1,642	1,668	1,698	1,726	1,746	1,778	1,785
80	84	89	85	85	87	87	87	88	89	89	90	92	93	94	96	97	99	99	99	99
283	282	282	284	286	285	285	288	286	291	299	301	301	301	313	310	311	316	316	316	316
382	384	386	387	390	391	391	391	392	394	395	395	396	398	398	400	402	405	407	409	409
15,229	15,281	15,423	15,448	15,479	15,525	15,541	15,560	15,582	15,634	15,665	15,684	15,699	15,706	15,715	15,737	15,707	15,730	15,765	15,776	15,787
515	519	527	533	539	543	545	548	551	557	562	565	569	571	578	588	591	596	601	607	614
560	571	576	594	603	621	617	617	636	643	651	663	672	673	674	702	730	735	735	758	760
31928	31940	31968	31999	32015	32024	32069	32088	32109	32133	32139	32158	32179	32198	32203	32222	32263	32272	32289	32304	32320
2,927	2,970	2,991	3,006	3,032	3,036	3,058	3,064	3,069	3,075	3,103	3,112	3,132	3,174	3,189	3,219	3,235	3,256	3,297	3,297	3,307
415	416	424	428	432	432	441	424	424	430	432	438	451	451	451	461	473	477	483	495	496
215	220	224	230	232	232	234	237	244	247	249	254	257	260	262	269	271	273	282	286	289
6,787	6,812	6,848	6,880	6,897	6,904	6,911	6,931	6,957	6,973	6,992	7,007	7,015	7,018	7,038	7,063	7,079	7,101	7,114	7,118	7,122
960	969	971	974	976	976	976	984	984	987	988	990	990	990	995	996	997	1,001	1,002	1,002	1,002
827	846	884	905	929	951	961	972	993	998	1,070	1,096	1,135	1,155	1,164	1,221	1,285	1,334	1,385	1,465	1,491
97	98	98	101	107	109	109	109	109	111	115	116	116	118	118	118	119	121	122	122	123
653	665	685	710	723	738	741	749	767	783	796	815	838	843	847	871	888	925	938	964	967
2,655	2,715	2,813	2,918	3,013	3,112	3,192	3,235	3,322	3,432	3,561	3,735	3,865	3,958	4,020	4,151	4,348	4,521	4,717	4,885	5,038
190	199	201	209	213	215	216	220	226	234	235	243	243	243	249	258	263	267	274	273	275
1,881	1,905	1,937	1,958	1,962	1,966	1,968	1,977	1,992	2,007	2,013	2,025	2,027	2,031	2,048	2,051	2,054	2,067	2,075	2,078	2,082
56	56	56	56	56	56	56	56	56	56	56	56	56	56	56	56	56	56	56	56	56
1,370	1,384	1,394	1,409	1,424	1,424	1,438	1,399	1,404	1,421	1,427	1,434	1,444	1,447	1,453	1,465	1,468	1,482	1,495	1,494	1,501
803	812	814	816	821	828	820	827	833	834	838	840	850	851	853	866	872	885	885	898	899
95	95	95	95	95	96	96	97	97	98	99	100	100	100	100	101	102	103	103	103	103
20	20	21	21	21	21	21	21	22	22	24	24	24	24	24	25	25	25	25	25	25

7/27/2020	7/28/2020	7/29/2020	7/30/2020	7/31/2020	8/1/2020	8/2/2020	8/3/2020	8/4/2020	8/5/2020	8/6/2020	8/7/2020	8/8/2020	8/9/2020	8/10/2020	8/11/2020	8/12/2020	8/13/2020	8/14/2020	8/15/2020	8/16/2020
21	22	22	23	23	24	24	25	25	25	25	25	26	26	26	26	27	27	27	28	28
1,492	1,493	1,538	1,567	1,581	1,603	1,627	1,633	1,668	1,698	1,714	1,731	1,751	1,768	1,797	1,847	1,882	1,890	1,893	1,896	1,898
408	428	434	442	453	458	464	475	490	508	515	521	535	544	555	566	573	582	587	600	599
3,304	3,408	3,454	3,626	3,694	3,747	3,765	3,779	3,845	3,932	4,002	4,081	4,137	4,150	4,154	4,199	4,347	4,383	4,423	4,492	4,506
8,445	8,518	8,715	8,909	9,005	9,224	9,356	9,388	9,501	9,703	9,869	10,011	10,189	10,293	10,359	10,468	10,648	10,808	10,996	11,147	11,224
1,799	1,807	1,822	1,822	1,838	1,844	1,844	1,844	1,849	1,851	1,852	1,857	1,857	1,858	1,863	1,875	1,875	1,882	1,888	1,896	1,896
4,418	4,423	4,425	4,431	4,432	4,432	4,432	4,437	4,437	4,437	4,437	4,441	4,441	4,441	4,444	4,444	4,450	4,450	4,453	4,453	4,453
582	583	584	584	585	585	586	586	587	587	587	589	590	591	591	593	593	594	594	597	597
580	581	581	581	585	585	585	585	587	587	587	590	591	591	591	591	593	593	593	593	593
5,931	6,117	6,333	6,586	6,843	7,022	7,084	7,292	7,402	7,627	7,747	7,927	8,109	8,186	8,359	8,553	8,765	8,913	9,141	9,345	9,452
3,509	3,563	3,642	3,671	3,752	3,825	3,840	3,842	3,921	3,984	4,026	4,117	4,186	4,199	4,229	4,351	4,456	4,538	4,573	4,669	4,702
26	26	26	26	26	26	26	26	27	27	29	30	31	31	34	34	38	38	40	40	40
834	839	845	857	867	871	876	883	887	899	908	913	925	930	933	940	951	959	966	973	975
152	160	173	177	189	197	197	200	210	217	223	229	235	237	239	246	246	251	265	269	269
7,608	7,638	7,654	7,670	7,692	7,700	7,714	7,723	7,742	7,770	7,791	7,822	7,840	7,845	7,846	7,866	7,881	7,905	7,932	7,937	7,955
2,906	2,924	2,932	2,946	2,965	2,971	2,975	2,980	2,996	3,007	3,013	3,023	3,036	3,041	3,044	3,069	3,086	3,105	3,113	3,128	3,133
335	335	349	349	358	358	358	365	365	368	368	380	380	380	387	387	395	395	402	402	402
709	719	724	731	735	740	742	744	751	752	760	764	772	773	775	783	790	796	804	810	813
3,786	3,812	3,883	3,925	3,949	3,949	4,007	4,024	4,051	4,096	4,146	4,207	4,207	4,263	4,287	4,313	4,361	4,402	4,430	4,430	4,507
8,536	8,551	8,580	8,595	8,609	8,626	8,638	8,648	8,657	8,659	8,691	8,709	8,721	8,735	8,741	8,751	8,769	8,790	8,804	8,813	8,837
3,458	3,478	3,488	3,493	3,506	3,515	3,523	3,530	3,536	3,551	3,565	3,577	3,585	3,591	3,604	3,612	3,620	3,631	3,636	3,639	3,641
121	121	121	123	123	123	123	123	124	124	124	125	125	125	126	126	126	126	127	127	127
6,405	6,421	6,422	6,443	6,450	6,457	6,457	6,463	6,471	6,478	6,506	6,524	6,520	6,519	6,526	6,533	6,539	6,555	6,566	6,586	6,592
1,620	1,629	1,634	1,640	1,646	1,654	1,654	1,660	1,670	1,677	1,681	1,689	1,698	1,701	1,707	1,707	1,726	1,739	1,745	1,752	1,759
1,201	1,213	1,220	1,233	1,243	1,253	1,253	1,255	1,266	1,273	1,280	1,301	1,301	1,307	1,307	1,312	1,323	1,325	1,335	1,346	1,367
1,501	1,563	1,611	1,611	1,693	1,693	1,711	1,753	1,753	1,804	1,825	1,874	1,874	1,896	1,912	1,944	1,989	2,011	2,043	2,080	2,084
47	52	55	58	60	61	61	64	65	66	70	74	75	75	75	79	79	81	81	82	82
1,790	1,820	1,865	1,903	1,924	1,964	1,969	1,982	2,010	2,050	2,092	2,134	2,160	2,168	2,172	2,204	2,249	2,287	2,313	2,343	2,347
100	102	103	103	103	105	105	107	108	109	110	112	112	113	118	120	120	121	121	125	126
317	321	324	328	332	332	332	332	332	335	340	345	345	345	348	351	356	360	361	361	361
409	409	411	415	415	416	417	417	418	418	419	419	419	419	419	419	420	422	423	423	423
15,804	15,825	15,798	15,809	15,819	15,830	15,836	15,846	15,857	15,842	15,849	15,860	15,869	15,874	15,878	15,890	15,885	15,893	15,903	15,876	15,903
619	626	632	635	642	651	654	655	658	667	669	675	681	685	690	693	695	697	703	711	714
765	785	806	827	856	858	858	874	889	917	927	947	977	985	991	1,009	1,024	1,058	1,073	1,097	1,100
32329	32338	32355	32368	32377	32395	32410	32419	32429	32431	32438	32449	32455	32465	32472	32487	32497	32509	32512	32526	32535
3,344	3,382	3,422	3,442	3,489	3,515	3,529	3,539	3,570	3,596	3,618	3,652	3,668	3,669	3,673	3,708	3,734	3,755	3,784	3,824	3,826
496	503	523	536	541	549	550	551	566	583	593	599	603	603	605	618	627	637	644	657	657
289	303	311	316	322	325	326	328	333	338	339	348	355	356	357	368	375	383	385	386	393
7,146	7,162	7,176	7,189	7,204	7,204	7,209	7,209	7,232	7,244	7,282	7,297	7,313	7,314	7,317	7,352	7,385	7,409	7,445	7,465	7,468
1,004	1,005	1,007	1,007	1,007	1,007	1,007	1,010	1,011	1,012	1,014	1,014	1,014	1,014	1,015	1,016	1,018	1,019	1,021	1,021	1,021
1,506	1,565	1,615	1,667	1,712	1,751	1,777	1,793	1,847	1,894	1,943	1,962	2,007	2,031	2,049	2,098	2,144	2,186	2,204	2,260	2,269
123	123	129	129	130	134	135	135	136	137	141	144	146	146	146	146	147	148	150	152	153
978	999	1,020	1,033	1,060	1,067	1,073	1,092	1,117	1,144	1,186	1,206	1,215	1,223	1,233	1,271	1,289	1,313	1,326	1,345	1,366
5,713	5,877	6,190	6,274	6,569	6,837	6,837	7,016	7,261	7,497	7,803	8,096	8,343	8,459	8,490	8,710	9,034	9,289	9,602	9,840	9,983
283	292	293	303	307	311	311	319	324	328	331	336	336	337	347	350	353	357	361	362	363
2,095	2,125	2,141	2,174	2,215	2,218	2,218	2,244	2,274	2,299	2,317	2,322	2,326	2,327	2,344	2,352	2,364	2,370	2,381	2,382	2,385
56	56	56	57	57	57	57	57	57	57	58	58	58	58	58	58	58	58	58	58	58
1,518	1,548	1,554	1,564	1,564	1,592	1,596	1,600	1,619	1,624	1,653	1,672	1,688	1,688	1,697	1,716	1,724	1,736	1,755	1,766	1,781
900	913	918	926	941	954	955	956	968	977	985	997	1,003	1,005	1,005	1,013	1,018	1,025	1,032	1,045	1,046
106	111	112	115	116	116	117	117	124	124	124	127	131	139	141	147	153	153	157	160	160
25	26	26	26	26	26	26	27	27	27	27	28	28	28	28	29	29	30	30	30	30

8/17/2020	8/18/2020	8/19/2020	8/20/2020	8/21/2020	8/22/2020	8/23/2020
28	29	29	29	30	31	32
1,925	1,936	1,944	1,974	1,996	2,011	2,017
603	619	631	641	663	674	687
4,506	4,529	4,634	4,684	4,688	4,756	4,771
11,242	11,342	11,523	11,686	11,821	11,988	12,134
1,896	1,899	1,900	1,903	1,910	1,918	1,918
4,456	4,456	4,457	4,458	4,460	4,460	4,460
597	599	600	601	602	604	604
593	595	595	600	600	600	604
9,539	9,758	9,932	10,049	10,168	10,274	10,325
4,727	4,794	4,849	4,904	4,998	5,092	5,132
40	41	43	45	46	47	47
987	989	1,005	1,016	1,021	1,030	1,036
273	282	291	298	304	306	307
7,967	7,993	8,017	8,044	8,066	8,083	8,089
3,135	3,165	3,180	3,191	3,208	3,218	3,220
405	405	411	411	419	419	419
818	830	842	856	864	872	881
4,526	4,554	4,609	4,637	4,687	4,687	4,746
8,842	8,848	8,876	8,888	8,901	8,921	8,921
3,650	3,661	3,669	3,674	3,685	3,691	3,694
127	127	128	129	130	131	131
6,592	6,608	6,618	6,634	6,634	6,655	6,659
1,758	1,767	1,792	1,800	1,799	1,813	1,817
1,396	1,402	1,414	1,417	1,419	1,425	1,426
2,128	2,128	2,190	2,190	2,237	2,240	2,248
84	84	84	89	89	90	91
2,348	2,396	2,431	2,465	2,494	2,521	2,531
128	130	130	132	135	136	137
362	368	371	373	376	376	378
423	424	427	428	428	429	429
15,916	15,925	15,926	15,932	15,941	15,943	15,946
718	723	729	734	739	743	745
1,105	1,130	1,162	1,200	1,215	1,227	1,227
32548	32553	32553	32559	32567	32582	32592
3,832	3,871	3,907	3,929	3,955	3,975	3,978
665	682	699	709	715	725	726
396	397	408	412	414	417	417
7,468	7,499	7,523	7,523	7,558	7,576	7,578
1,023	1,024	1,027	1,028	1,030	1,030	1,030
2,288	2,343	2,360	2,401	2,459	2,493	2,504
153	154	155	157	159	160	161
1,387	1,426	1,452	1,488	1,549	1,563	1,567
10,034	10,250	10,559	10,793	11,051	11,266	11,370
365	371	377	382	383	385	385
2,397	2,410	2,427	2,436	2,443	2,443	2,471
58	58	58	58	58	58	58
1,785	1,809	1,822	1,837	1,850	1,857	1,863
1,046	1,059	1,067	1,074	1,075	1,089	1,089
160	164	166	166	170	176	178
33	34	34	34	37	37	37

eTable 5. Facts behind face mask: cost and effectiveness

	**Issues**	**How to resolve**
Health Economic and Cost	Not everyone will be able to afford purchasing one-time-use disposable masks.<br>Worsened by global shortage of commercial supplies because production is based on society demands during non-pandemic.<br>Possible waste of natural resources and environments if universal masking in any situation in all states?	Homemade masks and face covering alternatives may not be as effective as commercially available masks. Schools and companies should provide free disposable masks in indoor entrances during ILI season if they can provide free toilet papers and hand towels. Financial supports from Tax deduction & governments.<br>Stockpiles at home and FEMA.<br>Reusable standardized cloth masks with daily washing because of the cost and availability.
Compliance / Adherence	Feasibility and Adaptations. Very difficult for healthy people including children younger than 2 years old for a long-time use.<br>Not everyone can wear because of health issues[#]	Culture difference: traditionally, Western societies consider only those infected should wear masks because of potential spreading the virus. In Asia, wearing a mask is not just mainly for protecting people from getting infected, but also minimizing the possibility of potential infection in you from spreading to other people. Education people that masks protect you and community.
Effectiveness	Works well if you know how to proper use face masks that meet the standards or approved by the FDA and NIOSH (e.g. even N95 need fit test for professional)	Education on the proper use, disposal of used face masks, daily washing for cloth mask, and hand hygiene (example: washing hands before touching mask; washing hands before and after disposing of mask, etc.).
Adverse effects	**Ignoring social distancing and hand hygiene.**<br>Symptoms**:** Skin breakdown, acne, impaired cognition, anxiety, headaches, trigger skin sensitivity, eczema, acne, shortness of breath, and **mask mouth** (bad breath, dental problems, Yeast and bacterial infections).<br>Society security and safety issues	Most symptomatic side effects go away once mask is removed, otherwise seek professional care.

ILI: influenza-like illness; FEMA: The Federal Emergency Management Agency. NIOSH: The National Institute for Occupational Safety and Health. #: reference in CDC3 in supplemental eTable 1.
